# Human FSH Glycoform α-Subunit Asparagine^52^ Glycans: Major Glycan Structural Consistency, Minor Glycan Variation in Abundance

**DOI:** 10.3389/fendo.2022.767661

**Published:** 2022-10-18

**Authors:** Viktor Y. Butnev, Jeffrey V. May, Alan R. Brown, Tarak Sharma, Vladimir Y. Butnev, William K. White, David J. Harvey, George R. Bousfield

**Affiliations:** ^1^ Department of Biological Sciences, Wichita State University, Wichita, KS, United States; ^2^ Department of Biochemistry, University of Oxford, Oxford, United Kingdom

**Keywords:** FSH, glycoform, oligosaccharide, mass spectrometry, clearance

## Abstract

Follicle-stimulating hormone (FSH), an α/β heterodimeric glycoprotein hormone, consists of functionally significant variants resulting from the presence or absence of either one of two FSHβ subunit N-glycans. The two most abundant variants are fully-glycosylated FSH24 (based on 24 kDa FSHβ band in Western blots) and hypo-glycosylated FSH21 (21 kDa band, lacks βAsn^24^ glycans). Due to its ability to bind more rapidly to the FSH receptor and occupy more FSH binding sites than FSH24, hypo-glycosylated FSH21 exhibits greater biological activity. Endoglycosidase F1-deglycosylated FSH bound to the complete extracellular domain of the FSH receptor crystallized as a trimeric complex. It was noted that a single biantennary glycan attached to FSHα Asn^52^ might preemptively fill the central pocket in this complex and prevent the other two FSH ligands from binding the remaining ligand-binding sites. As the most active FSH21 preparations possessed more rapidly migrating α-subunit bands in Western blots, we hypothesized that Asn^52^ glycans in these preparations were small enough to enable greater FSH21 receptor occupancy in the putative FSHR trimer model. Highly purified hFSH oligosaccharides derived from each FSH subunit, were characterized by electrospray ionization-ion mobility-collision-induced dissociation (ESI-IM-CID) mass spectrometry. FSHβ glycans typically possessed core-linked fucose and were roughly one third bi-antennary, one third tri-antennary and one third tetra-antennary. FSHα oligosaccharides largely lacked core fucose and were bi- or tri-antennary. Those αAsn^52^ glycans exhibiting tetra-antennary glycan *m/z* values were found to be tri-antennary, with lactosamine repeats accounting for the additional mass. Selective αAsn^52^ deglycosylation of representative pituitary hFSH glycoform Superdex 75 gel filtration fractions followed by ESI-IM-CID mass spectrometry revealed tri-antennary glycans predominated even in the lowest molecular weight FSH glycoforms. Accordingly, the differences in binding capacity of the same receptor preparation to different FSH glycoforms are likely the organization of the FSH receptor in cell membranes, rather than the αAsn^52^ oligosaccharide.

## Introduction

Follicle-stimulating hormone (FSH) is a heterodimeric glycoprotein hormone. Both FSHα and FSHβ subunits possess 2 potential Asn(N)-linked glycosylation sites (1). Human pituitary and urinary FSH preparations are complex mixtures of glycosylation variants that differ in the structures of N-linked oligosaccharides (so-called microheterogeneity) (2–4) as well as in the number of oligosaccharides attached to the 4 N-glycosylation sites (macroheterogeneity) (2). Both α-subunit glycosylation sites, Asn^52^ and Asn^78^, are always glycosylated in pituitary and urinary FSH preparations, while partial glycosylation of both FSHβ-subunit sites, Asn^7^ and Asn^24^, has been reported (1). FSH macroheterogeneity, the absence of one of the 2 FSHβ N-glycans, was initially detected by Western blotting, then confirmed by automated Edman degradation and MALDI-MS (5). Fully- and partially-glycosylated FSHβ subunit bands exhibit molecular weights of 24, 21, and 18 kDa in FSHβ-specific Western blots. We use FSHβ molecular weights to designate the corresponding FSH glycoform heterodimers, FSH24 (both Asn^7^ and Asn^24^ glycans present), FSH21 (Asn^24^ glycan missing), and FSH18 (Asn^7^ glycan missing), respectively. Hypo-glycosylated FSH preparations are designated FSH21/18, because most of these preparations contain both glycoforms and the first purified hypo-glycosylated FSH preparation was a 60:40 FSH21:FSH18 mixture (6). The electrophoretic mobilities of FSHα bands from different glycoform preparations vary. Nevertheless, FSHα typically migrates as a single band (6), rather than as mixtures of glycosylated, partially- and fully-deglycosylated bands. Thus, α-subunit glycosylation heterogeneity is essentially microheterogeneity, which can be evaluated by sequential release of Asn^52^, then Asn^78^ oligosaccharides (7).

FSH glycosylation macro- and micro-heterogeneity, have been observed to change in different physiological states, as indicated by altered isoelectric patterns [reviewed in (8)]. FSH microheterogeneity results from as many as 100 structural variants of complex-type glycans differing by composition and number of branches, the presence or absence of core fucose or bisecting GlcNAc residues, and terminal sialylation patterns (2). Sialylation increases structural variety in several ways including, altering the number of negative charges (-1 for each Neu5Ac residue), position on partially sialylated, multi-antennary glycans, and linkage to underlying galactosyl residues, either α2-3 or α2-6 (9).

Glycosylation microheterogenity is better characterized for pharmaceutical preparations of recombinant hFSH than for pituitary or urinary hFSH. Proteinase K digestion of reduced, carboxymethylated pituitary hFSH revealed only 24 glycan structures (10, 11) as compared with 68-84 liberated by PNGaseF and characterized by nanoESL-MS (2). Reduction, carboxamidomethylation, chymotryptic digestion, followed by LC-MS appears to provide almost quantitative oligosaccharide characterization of recombinant hFSH produced by Chinese hamster ovarian (CHO) cells (12–14). For example, a recent study reported 28 glycan structures following glycopeptide analysis of several GonalF and Bemfola lots (12). In our hands, PNGaseF released a total of 35 glycan structures from recombinant hFSH preparations, GonalF and Follistim (Bousfield & Harvey, unpublished data from our laboratory). CHO cell-expressed glycoproteins exhibit reduced microheterogenity due to limited glycosyltransferase expression. As CHO cells do not express GlcNAc transferase III, no bisecting GlcNAc residues are present (15). CHO cells express α3-sialyltransferases, but not α6-sialyltransferases (15), therefore, all Neu5Ac residues are linked α2-3. Neu5Ac linked α2-3 prevents FSH binding to the asialo-glycoprotein receptor in the liver (16). In addition, CHO cell-produced recombinant hFSH preparations exhibit limited microheterogeneity at 3 of 4 glycosylation sites (12). Bi-antennary glycans with 1 or 2 Neu5Ac residues comprise the majority of the glycans at βAsn^24^, and both FSHα glycosylation sites. Core fucose is absent in those derived from the FSHα subunit and present in the βAsn^24^ glycans, but only in a fraction of those attached to βAsn7, where most of the microheterogeneity occurs. Both core-fucosylated and non-fucosylated versions of 11 glycans, along with 7 exclusively non-fucosylated glycans are attached to βAsn^7^. In contrast, pituitary and urinary hFSH glycans include many of the structural variations absent in recombinant hFSH expressed in CHO cells.

The combined macro- and micro-heterogeneity of FSH glycosylation create differential charge patterns responsible for FSH isoforms reported in physiological fluids and tissue extracts (8). Altered biological activity exhibited by FSH isoforms led to the hypothesis that carbohydrate modulates hormone activity (17). Chemical deglycosylation of FSH preparations attended by retention of receptor-binding activity, but loss of ability to stimulate cAMP and steroidogenesis supported this hypothesis and suggested the mechanism involved efficiency of signal transduction (18, 19). Elimination of glycosylation sites by mutagenesis localized FSH biologic activity primarily to α-subunit Asn^52^ (20–22). Conflicting results were obtained following FSHβ glycosylation site mutation. Either FSH activity increased or decreased in the absence of one of these glycans (21, 23, 24).

Naturally occurring FSH macroheterogeneity results from a variation in N-glycosylation efficiency of oligosaccharyl transferase in processing the pre-FSHβ subunit. This manifests as high pituitary FSH21 abundance in young women that becomes equivalent to that of FSH24 in perimenopausal age pituitary FSH, and reaches low abundance in postmenopausal pituitary FSH equivalent to that observed in postmenopausal urinary FSH (2). Although the original glycosylation modulation hypothesis focused on FSH biological activity (17), macroheterogeneity also impacts FSH receptor (FSHR) binding. Hypo-glycosylated FSH21/18 preparations bind FSHRs more rapidly than FSH24, exhibit a higher affinity for FSHR, and occupy more FSH binding sites than FSH24 under identical experimental conditions (6, 25). Greater FSH21/18 receptor-binding activity is associated with greater biological activity both *in vivo* and *in vitro*. In human granulosa cell-like KGN tumor cells, HEK293 cells transfected with human FSHR, and primary cultures of porcine granulosa cells, FSH21/18 exhibited greater biological activity than FSH24 (26–28). FSH21/18 was also more active stimulating cultured murine ovarian follicles (29). FSH21/18 and FSH24 displayed differential patterns of gene expression in *Fshb*-null mouse ovaries (30) and immature mouse ovaries (31) following 2-hr treatment *in vivo*. In non-gonadal tissues, FSH24 has been reported to be more active than FSH21/18 at promoting osteoclast differentiation (32). While our focus in recent years has been on FSH glycosylation macroheterogeneity, microheterogeneity should not be completely ignored. Structural studies on the FSH receptor, suggested the structure of the αAsn^52^ oligosaccharides in hypo-glycosylated FSH might contribute to increased FSHR binding.

FSHR cloning suggested a monomeric GPCR structure with large extracellular domain (33), dismissing previous FSH crosslinking data that had suggested several components comprised the FSHR (34, 35). Nevertheless, evidence that the FSHR was associated with a larger complex than that indicated by its predicted primary structure appeared a few years later (36). FSHR dimers or oligomers were subsequently observed in co-immunopurification and fluorescence resonance energy transfer experiments (37) and independently confirmed by ultracentrifugation and Western blot analysis of the resulting fractions (38). While these studies supported the existence of FSHR dimers or oligomers, the extent of oligomerization was not determined. Negative cooperativity associated with binding of all glycoprotein hormones to their cognate receptors (39) revealed only a portion of the bound thyroid-stimulating hormone (TSH), luteinizing hormone (LH), or FSH tracers was dissociated after 3-24 hr incubation in the presence of excess unlabeled hormone. Thus, the majority of these receptor populations appeared to be monomers (40), as additional binding site(s) provided by receptor oligomerization are needed for excess cold ligand binding to initiate neighboring receptor conformational changes that dislodges bound tracer ligand. Otherwise, in the absence of unlabeled competitor, ^125^I-FSH remains bound to its receptor (39, 41). FSHR oligomerization characterized using a super-resolution microscopic technique employing photoactivatable dyes and localization microscopy (PD-PALM) revealed that in FSHR-transfected HEK293 cells 70% of FSHRs were monomeric (42). This increased to 80% after either 2-min incubation with hypo-glycosylated FSH preparations, eFSH and hFSH21/18, or after 5 min with hFSH24, and returned to 70% by 15 min (42). These results conflict with crystal structures of recombinant FSH and the high affinity FSHR binding site appearing to dimerize in the crystals, as well as in solution (43) and the entire FSHR extracellular domain and FSH appearing as trimers, supported by binding studies (44, 45). PD-PALM analysis of a biased FSH agonist revealed increased FSHR oligomerization to 50% by 15 min after hormone addition (42). As receptor binding experimental incubation times range from 1-24 hr, perhaps longer exposure to deglycosylated FSH promotes bulk receptor association. Cryogenic electron microscopy (cryo-EM) structures of the LH/CG receptor and TSH receptor provided insight into activation of the monomeric receptor population based on single particle analysis of monodisperse solubilized receptors (46, 47).

The crystal structure of deglycosylated FSH bound to the entire FSHR extracellular domain (FSHR_ECD_) trimeric structure resulting from interactions between the receptor hinge regions not present in the dimeric recombinant FSHR high affinity hormone binding domain complexes (FSHR_HB_) reported earlier (43, 44). Recombinant FSHR_ECD_ trimers formed a central pocket within which only a single FSHα Asn^52^ glycan could be accommodated (45). In the trimer model a bi-antennary glycan was predicted to preclude simultaneous binding of a second FSH ligand to FSHR trimers. The three FSH ligands observed in the actual crystal structure were effectively deglycosylated by endoglycosidase F1 digestion reducing their oligosaccharides to single GlcNAc residues (44). Evidence in support of a trimeric FSHR model was provided when elimination of the αAsn^52^ glycosylation site increased FSH binding to CHO cells expressing the FSHR 3-fold. Furthermore, co-incubation with an allosteric FSHR modulator increased fully-glycosylated FSH binding 3-fold and altered the ratio of β-arrestin-FSHR binding from 1:3 to 1:1 (45). PD-PALM studies on the LH/CGR indicated a variety of oligomeric forms ranging from dimers to greater than 9 receptors (48). Molecular modeling based on patterns of oligomerized receptors suggested a variety of receptor-receptor interactions *via* the transmembrane domains. A similar pattern of FSHR oligomerization has been observed using the same PD-PALM approach during the first 15 min of FSH binding (42).

This project was stimulated by the potential for a bi-antennary αAsn^52^ glycan to fill the central pocket formed by the FSH receptor trimer model, thereby precluding simultaneous binding by additional FSH ligands (44, 45, 49). Following 3-hr incubation, ^125^I-FSH21/18 tracer saturates at a level 2- to 3-fold higher than FSH24 tracer, depending on the receptor preparation (6, 25). As some hypo-glycosylated hFSH preparations possessed faster migrating α-subunit bands than observed in this pituitary FSH preparation (6), the size and abundance of small oligosaccharides could influence FSHR binding. Because single FSHα bands merely shift their mobility, we characterized N-glycan populations at both glycosylation sites and found bi- and tri-antennary oligosaccharides predominated in both positions of a pituitary FSH preparation possessing both FSH24 and FSH21. To determine if small αAsn^52^ glycan populations increased in abundance with decreasing FSH size, we selectively removed αAsn^52^ glycans from a series of 25-50 µg pituitary FSH glycoform fractions from which FSH24 and FSH21 preparations are derived. Mass spectrometry revealed that tri-antennary oligosaccharides were the most abundant glycans attached to all hFSHα subunits at Asn^52^ regardless of FSH glycoform size. Analysis of αAsn^52^ glycans from a 2-mg FSHα sample revealed low abundance glycans in the mass range typical of tetra-antennary glycans were actually tri-antennary with lactosamine repeats. As immunoaffinity chromatography separated 21kDa-FSHβ from 24kDa-FSHβ, we also characterized βAsn^7^ glycans as well as total FSHβ glycans, expanding our knowledge of pituitary FSH microheterogeneity to both subunits and 3 of 4 FSH glycosylation sites.

## Materials and Methods

### Materials

Human pituitary glands were obtained from the National Hormone and Pituitary Program *via* Dr. James A. Dias, the University at Albany, Albany, NY. The highly purified pituitary hFSH preparations AFP4161A and AFP7298A were purchased from the National Hormone and Pituitary Program and Dr. A.F. Parlow. AFP4161A FSH subunits were dissociated by overnight incubation in 6 M GuHCl and FSHα purified by reverse-phase HPLC followed by Sephadex G-100 chromatography (50). Anti-human α-subunit monoclonal antibody 4882 was the generous gift of SPD Development Co., Ltd. (Bedford, UK). FSH ELISA kits were purchased from Immuno-Biological Laboratories, Inc. (IBL America), Minneapolis, MN. Recombinant hFSH glycoform preparations GH_3_-FSH21 and GH_3_-FSH24 were purified in our laboratory (25).

### FSH Subunit Glycoform Isolation From Purified Pituitary hFSH

A 0.5 mg sample of highly purified hFSH (AFP7298A) was dissolved in 100 µL 6 M guanidine-HCl in 0.01% TFA containing 30% acetonitrile, pH 4, and incubated at 37°C overnight. The dissociated subunit solution was diluted with 20 ml 0.05 M sodium phosphate buffer, pH 7.5, and applied to a 2-mL anti-α subunit monoclonal antibody (MAb) 17-6.E5.A4 immunoaffinity column and an 18-mL MAb 15-1.E3.E5 (AB_281484) anti-FSHβ subunit immunoaffinity column linked in series. Following sample loading and washing with the same buffer, the columns were separated. The FSHα subunit fraction was eluted from the 17-6.E5.A4 antibody column with 0.1 M glycine-HCl, pH 2.7, containing 0.5 M NaCl. Fully-glycosylated, 24kDa-FSHβ was eluted from the 15-1.E3.E5 column with 0.1 M glycine-HCl, pH 2.7, containing 0.5 M NaCl, while hypo-glycosylated, 21kDa-FSHβ was subsequently eluted with 3 M guanidine-HCl.

### Oligosaccharide Isolation From FSH Subunits

#### Sequential Oligosaccharide Release From FSHα

Oligosaccharides were sequentially released from FSHα subunit preparations by PNGaseF digestion of native (Asn^52^ glycans released), then reduced, carboxymethylated FSHα (Asn^78^ glycans released), as previously described (7). Oligosaccharides were separated from partially or completely deglycosylated FSHα by ultrafiltration in Millipore (Billerica, MA) Amicon Ultra-4, 10,000 MW cutoff, cartridges and recovered from the filtrate fraction by evaporation in a Thermo Fisher Scientific (Waltham, MA) Savant SpeedVac.

#### Oligosaccharide Release From FSHβ Glycoforms

Samples of 24kDa-FSHβ and 21kDa-FSHβ were reduced and carboxymethylated (51), the buffer exchanged with 0.2 M ammonium bicarbonate, pH 8.5, by ultrafiltration, and subjected to overnight PNGaseF digestion at 37°C (9). Oligosaccharides were separated from deglycosylated protein using the Acquity UPLC system employing a Phenomenex (Torrance, CA) reverse-phase Kinetex C8 column. The column was equilibrated at 50°C with 0.01% TFA containing 5% acetonitrile at a flow rate of 0.7 mL/min. Oligosaccharides emerged in the void volume peak, which was collected manually, and carbohydrate recovered by evaporation in a Speed Vac.

### Characterization of FSH αAsn^52^ Oligosaccharides as a Function of FSH Size

#### Pituitary FSH Glycoform Isolation

FSH was isolated from dried human pituitaries by extraction in water maintained at pH 5.5 with HCl, followed by extraction in 0.1M saturated ammonium sulfate, pH 4.1. Solubilized FSH was captured from the combined extracts by immunoaffinity chromatography using MAbs 15-1.E3.E5 and 4882, with individual MAb chromatogram development. FSH glycoform fractions were obtained by triple-Superdex 75 chromatography, as previously reported (25). The amount of FSH in each fraction was estimated by size exclusion chromatography (SEC) using a Waters (Milford, MA) 1.7 µm particle size, BEH200 UPLC SEC column. Isocratic 0.2 M ammonium bicarbonate/20% acetonitrile chromatograms were developed with a Waters H-class Acquity UPLC system. Western blot analysis was performed on 1 µg samples (6). FSH receptor-binding was performed with 10 µg samples after serial dilution (52).

#### Selective Release of αAsn^52^ Oligosaccharides From Dissociated FSH Glycoform Samples

Eight, 25- or 50-µg FSH glycoform samples were dissociated into subunits by overnight incubation at 37°C in 100 µL 6 M guanidine-HCl in 0.01% trifluoracetic acid, pH 4, containing 30% acetonitrile. Dissociated subunits were transferred to 0.2 M ammonium bicarbonate buffer, pH 8.5, by ultrafiltration in Amicon Ultra-4, 10,000 MW cutoff, centrifugal ultrafiltration cartridges. A 1 µL aliquot containing 2.5 mU Prozyme (Hayward, CA) PNGaseF was diluted in 650 µL 0.2 M ammonium bicarbonate buffer, pH 8.5, and 5 or 10 µL aliquots added to each 25 or 50 µg FSH sample, respectively, and incubated overnight at 37°C. Western blot analysis of two before and two after 1-µg samples was performed with antibodies specific for FSHα (HT13) and FSHβ subunits (15-1.E3.E5) to confirm selective deglycosylation of FSHα. Oligosaccharides were separated from residual glycoprotein by reverse-phase UPLC using a Phenomenex (Torrance, CA) Kinetex C4 column. Oligosaccharides were recovered from the filtrate by evaporation in a SpeedVac. Oligosaccharide mass spectrometry was performed as described below.

### Mass Spectrometry

#### Sample Preparation for Mass Spectrometry

Native and *Arthrobacter ureafaciens* sialidase-deglycosylated oligosaccharide samples were dissolved in 5 µL water. After applying 1 µL samples to a Nafion membrane for about 1 hour, treated oligosaccharide samples were diluted with 2 µL water and 3 µL methanol. A 0.2 µL aliquot of 0.1 M ammonium phosphate was then added. Each sample was centrifuged at 10,000 rpm for 1 min, then infused into a Waters (Milford, MA) ESI, Synapt G2 ion mobility mass spectrometer with Waters long, thin-wall capillaries. Negative ion MS, MS/MS and ion mobility data were collected using Waters (Milford, MA) MassLynx 4.1 and interpreted as described earlier (53, 54). Negative ion CID provides detailed information on structural features of N-glycans such as the location of fucose residues (differentiation between core and antenna fucose by the mass of the 2,4A ion from the reducing-terminal GlcNAc residue) and the presence of bisecting GlcNAc residues (abundant D-221 ion) as detailed previously (54).

#### Sialic Acid Linkage Analysis of Selectively Released αAsn^52^ Oligosaccharides From Purified FSHα

FSH subunits from hFSH preparation AFP4161A were purified by reverse-phase HPLC (50). FSHα Asn^52^ glycans were released by selective PNGaseF digestion of a 2 mg FSHα sample as described above. Chemical desialylation of a 1 µg glycan sample involved incubation in 2 µL 1% acetic acid for 30 min at 80°C followed by ESI-MS evaluation. Over 30 neutral glycan ions were identified and 17 proposed structures confirmed by collision induced dissociation (55–57). Another 1 µg glycan sample was purified with Nafion, dried, then heated in 20 µL with 4-(4,6-dimethoxy-1,3,5-trazin-2-yl)-4-methyl-morpholinium chloride (DMT-MM) for 1.25 hr to derivatize sialic acids and stabilize them toward MALDI-TOF-MS. The derivatized glycans were dried by evaporation, dissolved in 1 µL water, purified on Nafion for 10 min, and the oligosaccharide derivatives characterized by MALDI-TOF-MS from DHB (58).

#### MS Data Analysis

For each FSH subunit glycan sample, an ESI spectrum was collected, along with mobility-extracted singly, doubly and triply charged ion spectra (**Supplement Figures S1**-**S4**). The results of these analyses were recorded in separate ion tables (see **Supplement Tables S1**-**S12**). Many of the glycans gave several different ions (e.g. different charge states, sodium salts or [M+H]^-^ and [M+H_2_PO_4_]^-^ ions). These ions were brought together in **Supplement Table S13**. Glycan heterogeneity is illustrated in **Supplement Table S14**. For quantitative evaluation, peak heights of the monoisotopic ion and up to four of the ^13^C peaks were summed to give a measurement for each ion species. Then, the different ions from each glycan were summed to give the value for that glycan and results listed in **Table 1**. Because the quantitative figure for each glycan is the sum of several ions that are formed with unknown but different ionization efficiencies, the numbers do not represent the absolute amounts of each glycan but can be used to draw comparisons between the samples.

**Table 1:** Composition and abundance of FSH glycans (all ions).

Glycan	Glycan mass	Composition						Quantitation (% total)				Structure of neutral glycan
		Hex	HexNAc	Fuc	Neu5Ac	HSO_3_	H_2_PO_4_	β24	β21	αN78	αN52	
1	910.3	3	2	0	0	0	0	–	–	–		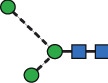
2	1234.4	5	2	0	0	0	0	–	–	0.07	–	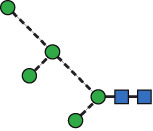
3	1314.4				0	0	1	–	–	–		
4	1476.5	6	2	0	0	0	1	–	–	–		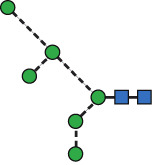
5	1113.3	3	3	0	0	0	0	–	–	–		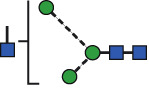
6	1259.5	3	3	1	0	0	0	–	0.09	0.12	0.05	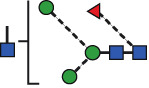
7	1275.5	4	3	0	0	0	0	–	–	0.09	–	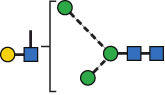
8	1566.5				1	0	0	–	–	0.61	0.25	
9	1421.5			1	0	0	0	–	0.07	0.03	–	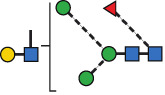
10	1712.6				1	0	0	0.14	–	–	–	
11	1316.5	3	4	0	0	0	0	–	–	–		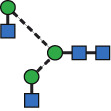
12	1607.6				1	0	0	–	–	0.15	0.08	
13	1396.4				0	1	0	0.01	0.06	0.22	0.14	
14	1462.5			1	0	0	0	0.01	–	0.03	–	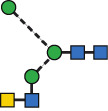
15	1542.5				0	1	0	0.01	0.05	0.10	0.06	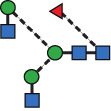
16	1478.5	4	4	0	0	0	0	–	–	–		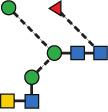
17	1769.6				1	0	0	–	–	0.11	0.07	
18	1558.5				0	1	0	–	0.06	0.10	0.10	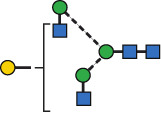
19	1624.6			1	0	0	0	–	–	0.02	0.02	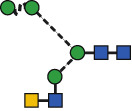
20	1915.6				1	0	0	0.24	0.38	–	–	
21	1931.7	5	4	0	1	0	0	–	–	0.12	0.13	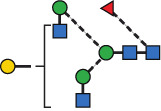
22	2222.8				2	0	0	0.45	0.58	20.28	13.63	
23	1720.5				0	1	0	–	–	0.04	0.04	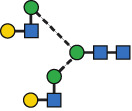
24	2011.6				1	1	0	–	–	–		
25	2077.7			1	1	0	0	2.03	1.84	0.24	0.22	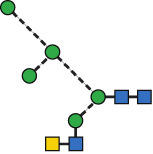
26	2368.8				2	0	0	13.36	11.17	1.02	0.96	
27	2157.7				1	1	0	0.67	0.43	–	–	
28		3	5	0	0	0	0	–	–	–		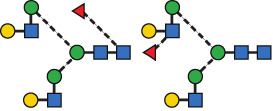
29	1810.7				1	0	0	–	–	0.02	0.01	
30	1599.5				0	1	0	–	–	0.06	0.04	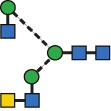
31	1745.6			1	0	1	0	–	–	0.06	0.04	
32	1681.6	4	5	0	0	0	0	–	–	–		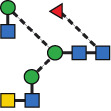
33	1972.7				1	0	0	–	–	0.12	0.09	
34	2263.8				2	0	0	0.18	0.38	19.75	9.89	
35	1923.6				0	1	0	–	–	0.06	0.06	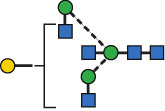
36	2052.7				1	1	0	0.12	0.92	3.69	3.06	
37	2118.8			1	1	0	0	0.55	0.76	0.26	0.10	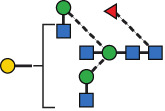
38	2409.9				2	0	0	0.85	0.73	–	–	
39	1907.6				0	1	0	0.01	0.01	0.03	0.02	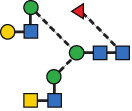
40	2198.7				1	1	0	1.29	1.84	0.17	0.23	
41	1843.7	5	5	0	0	0	0	–	–	–		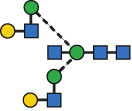
42	2134.8				1	0	0	–	–	0.03	0.05	
43	2425.8				2	0	0	0.34	0.63	10.14	9.63	
44	2214.7				1	1	0	–	–	–		

A similar approach was taken for FSH glycoform αAsn^52^ oligosaccharide samples. The ESI, single, doubly, and triply charged spectra can be found in the supplement as **Figures S5**-**S8** and **Tables S15**-**S17**. Composition and quantitative data were assembled into **Table S18**.

### FSHR-Binding Assay

Ten µg samples from 25 FSH-containing column fraction tubes were each added to a 12 x 75 mm polystyrene tube containing 990 µl RLA buffer, then serially diluted 1:10 four times. FSH receptor-binding activities were measured in a FSH radioligand assay using Chinese hamster ovarian (CHO) cells expressing hFSH receptors and ^125^I-hFSH24 as tracer (6). Based on the results of this experiment, six representative fractions were selected, serially diluted and evaluated in the radioligand assay. The assay of these 6 samples was repeated twice. Initial serial dilutions were based on SEC quantification, but were later corrected for protein recovery by amino acid analysis.

### FSH Serum and Tissue Accumulation Studies

All mouse procedures were approved by the Wichita State University IACUC. CD-1 mice were obtained from Charles River. All FSH injections were intra-peritoneal (IP), as our collaborators use this mode of administration for *in vivo* studies of FSH glycoform preparations (30, 31). Up to six 20-µl blood samples were collected from the retro-orbital plexus, the blood allowed to clot and 10 µl serum collected following centrifugation. FSH concentrations were measured either by using an IBL America ELISA kit, which measures all hFSH glycoforms equivalently, or by counting ^125^I in a Perkin-Elmer Wallac (Turku, Finland) Wizard^2^ model 2470 automatic gamma counter. Unlabeled hormone IP injections consisted of 10 µg pituitary hFSH (AFP7298A) and samples were collected at 20, 40, 60, 120, 180, and 240 min. Following IP injection of 1 µg ^125^I-labeled pituitary hFSH, samples were collected at 10 min intervals. At 70 min, the mice were euthanized, tissues removed and weighed in tared 12 x 75 mm polypropylene tubes and counted. Data are reported as cpm/mg tissue and total ^125^I-FSH uptake.

## Results

### FSH Subunit Glycoform Isolation

**Figure 1:**
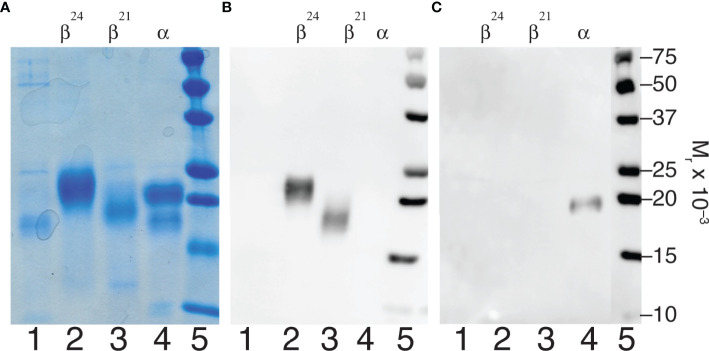
FSH subunit purification. Characterization of subunits derived from highly purified human pituitary FSH (AFP7298A). **(A)** SDS-PAGE of 5 µg subunit samples followed by Coomassie Blue staining. **(B)** Anti-FSHβ Western blot of 1 µg subunit samples using 15-1.E3.E5 as primary antibody. **(C)** Western blot of 1 µg subunit samples using anti-hCGα monoclonal antibody, HT13, as primary antibody. In both cases sheep anti-mouse IgG-HRP complex was the secondary antibody. Samples were loaded in the same order in each experiment. Lane 1, unbound material; lane 2, 24kDa-FSHβ; lane 3, 21kDa-FSHβ; lane 4, FSHα; lane 5, BioRad molecular weight markers, as indicated.

Dissociated pituitary hFSH subunits were purified using anti-FSHβ MAb 15-1.E3.E5 and anti-human α-subunit MAb 17-6.E5.A4 immunoaffinity columns. The former separated fully- from hypo-glycosylated FSHβ variants and the latter produced FSHα (**Figure 1**). The pH 2.7 buffer released 24kDa-FSHβ from the anti-FSHβ antibody column, while 3 M GuHCl released 21kDa-FSHβ. The anti-α antibody column captured the FSHα subunit, which was released with pH 2.7 buffer. SDS-PAGE indicated 24kDa-FSHβ was the most abundant component of the pH 2.7 fraction and 21kDa-FSHβ was the most abundant component of the 3 M GuHCl fraction (**Figure 1A**, lanes 2 and 3). The FSHα subunit preparation included three bands that suggested nicking of the αL2 cystine knot loop (**Figure 1A**, lane 4). This nick produces two fragment bands following reduction of disulfide bonds; a 17.5 kDa C-terminal glycoprotein band that migrates just ahead of the intact 22 kDa intact α-subunit band and a 10 kDa N-terminal peptide fragment band (59). Low level mouse antibody contamination was indicated by faint 50 kDa heavy chain and 25 kDa light chain bands in Coomassie Blue stained SDS gels (**Figure 1A**, lanes 1 and 3). Neither IgG band was detectable in the Western blot employing rabbit anti-mouse-HRP in any subunit preparation (**Figure 1B**, lanes 2 and 3 and **Figure 1C**, lane 4), indicating very low antibody contamination. The anti-α, HT13 Western blot did not detect the 17.5 kDa, C-terminal α-subunit glycopeptide fragment band (**Figure 1C**, lane 4), which possesses the primary epitope for this antibody (60). Only the intact, 22 kDa FSHα band was detected. However, the intensity of the band was low, suggesting the 17.5 kDa band was below the limits of detection (**Figure 1A**, lane 1). Alternatively, the αL2 loop nick may have affected antibody binding. A subsequent SDS-PAGE analysis of this FSHα preparation made 3 weeks later revealed reduced intensity of the intact α-subunit band staining, and increased intensities of both low MW bands (data not shown). Moreover, Edman degradation of the unbound fraction, which primarily possessed the 17.5 kDa band, revealed internal αL2 loop sequences that were consistent with proteolytic degradation (61).

### Site-Specific Analysis of Human Pituitary FSH Oligosaccharide Populations

**Figure 2:**
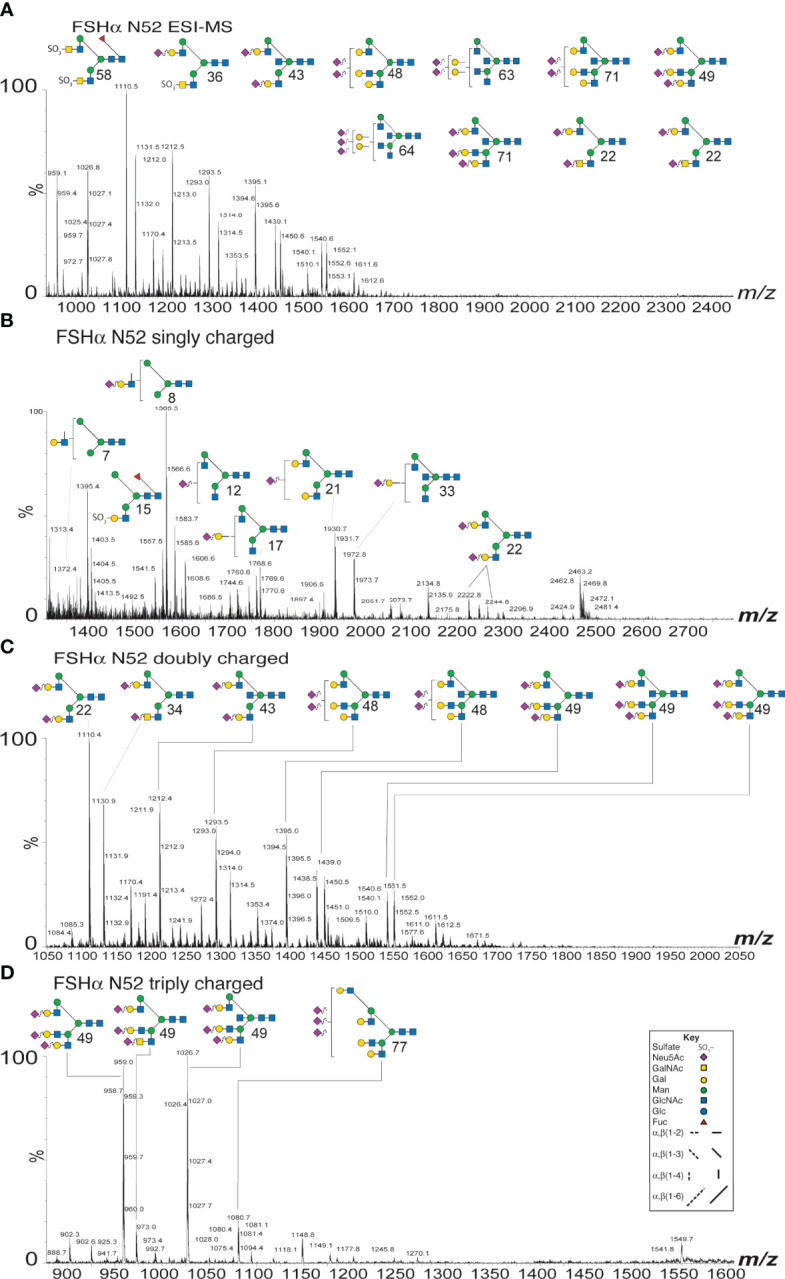
ESI-MS and ion-mobility MS spectra for FSH αAsn^52^ glycans. **(A)** Nano-ESI-MS spectrum. **(B)** Singly charged ion mobility MS spectrum. **(C)** Doubly charged ion mobility MS spectrum. **(D)** Triply charged ion mobility MS spectrum. Glycan structures displayed using the CFG/Oxford system, which identifies monosaccharides with colored symbols and linkages without the use of labels (see key). The glycan numbers correspond to **Table 1**.

Purification of both FSH subunits provided the opportunity to expand our knowledge of FSH microheterogeneity. Individual FSHα glycosylation site glycan populations were obtained by sequential PNGaseF digestion of native and reduced, carboxymethylated FSHα. PNGaseF digestion of reduced, carboxymethylated 21kDa-FSHβ liberated Asn^7^ glycans, while. PNGaseF digestion of reduced, carboxymethylated 24kDa-FSHβ released a mixture glycans from both Asn^7^ and Asn^24^. As our attention was initially focused on αAsn^52^ glycans, spectra for this glycan population are illustrated in **Figure 2**. The ESI-MS spectrum revealed the bi-antennary (such as structures 22, 36, 43, and 58, see **Table 1** for glycan identification) and tri-antennary glycans (48, 49, 63, 64, and 71). Low abundance hybrid type glycans, 7, 8, 12, 15, and 17 were observed in the singly charged spectrum. The doubly charged spectrum comprised largely bi-antennary (22, 34, and 43) and tri-antennary (48 and 49) glycans. The triply charged spectrum featured fully-sialylated tri-antennary glycan ions (49).

The ESI-MS spectra were similar for both of the FSHβ glycoforms (**Supplement Figures S1A, B**) and these both differed from the Asn^78^ and Asn^52^ spectra, which were similar to each other (**Supplement Figures S1C, D**). Core fucose was found in almost all FSHβ glycans and was absent in most FSHα glycans. Ion mobility MS revealed very low abundance glycans, which were likely obscured in total glycan spectra provided by ESI-CID alone (**Supplement Figures S2**-**S4** and **Tables S1**-**S12**). Singly charged spectra (**Supplement Figure S2**) revealed oligomannose to tri-antennary glycans in the FSHβ spectra (10-11 glycan ions, Supplement **Tables S1**, **S2**) and oligomannose to bi-antennary glycans in FSHα spectra (19-22 glycan ions, **Supplement Tables S3**, **S4**). Doubly charged glycans in the FSHβ spectra (73 glycan ions, **Supplement Tables S5**, **S6**) ranged from bi-antennary to tetra-antennary while FSHα glycans (19-32 glycan ions, **Supplement Tables S7**, **S8**) ranged from bi-antennary to tri-antennary (**Supplement Figure S3**). Triply charged glycans (25 glycan ions in FSHβ spectra and 4-9 glycan ions in FSHα spectra, (**Supplement Figure S4** and **Tables S9**-**S12**) ranged from tri-antennary to tetra-antennary types in all spectra. Composition and relative abundance data derived from these analyses, as well as subsequent FSH glycoform αAsn^52^ analyses are compiled in **Table 1**.

**Figure 3:**
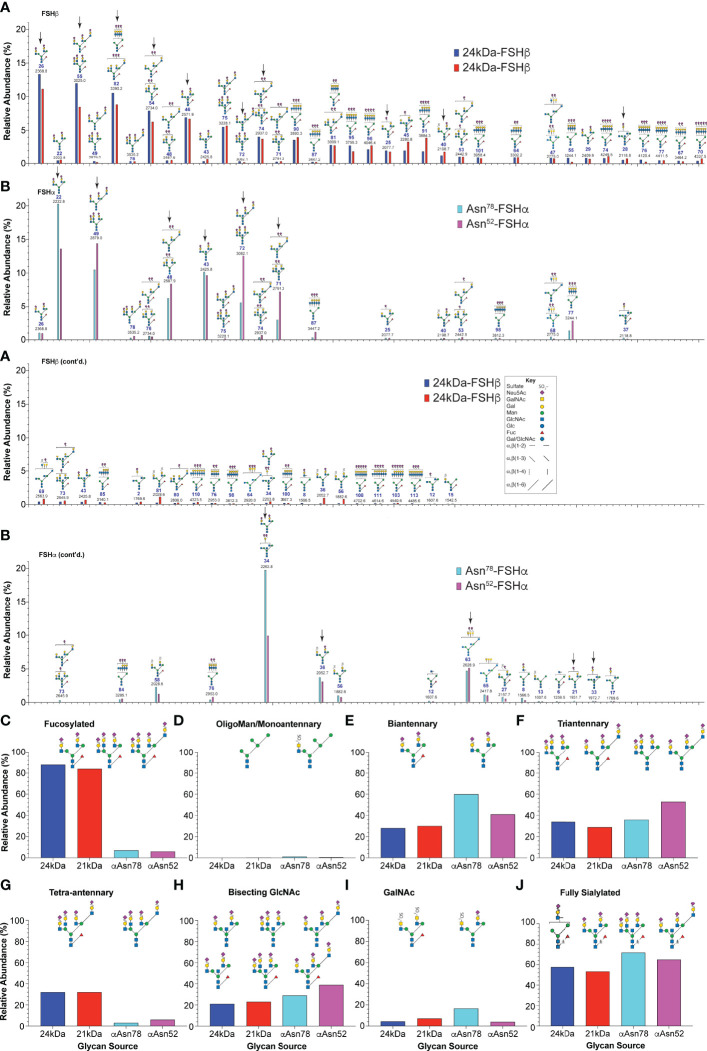
Human pituitary FSH glycan populations. **(A)** Glycans derived from 24kDa- (blue) and 21kDa-FSHβ (red) roughly in order of core fucosylated 24kDa-FSHβ glycan abundance, interrupted by otherwise identical structures lacking core fucose. **(B)** Glycans derived from αAsn^78^ (cyan) and αAsn^52^ (magenta) shown in the same order as in panel **(A)** Glycan abundances are presented as % of total glycans observed using the values in **Table 1**. Glycan structures are displayed using the hybrid Oxford Glycobiology Institute/Consortium for Functional Glycoscience system (1, 62). The former identifies linkages without the use of labels, while the latter uses a proposed symbol and color scheme for individual monosaccharide residues (shown in the Key). Arrows identify those glycans observed in site-specific glycopeptide studies (10, 11) **(C)** Relative abundance of glycans possessing core fucose residues. **(D)** Relative abundance of oligomannose or hybrid type glycans. **(E)** Relative abundance of biantennary glycans, as defined by the presence of the branch-initiating GlcNAc. **(F)** Relative abundance of tri-antennary glycans. **(G)** Relative abundance of tetra-antennary glycans. **(H)** Relative abundance of glycans possessing a bisecting GlcNAc residue. **(I)** Relative abundance of glycans possessing a GalNAc residue. **(J)** Glycans possessing enough Neu5Ac to terminate all branches.

Data extracted from the ion mobility-MS spectra identified 81 glycan species representing as many as 103 glycans. The greater number of proposed structures than glycan species detected resulted either from ambiguity when only compositions were inferred from the data or when more than one structure was found in fragmentation data obtained for 9% of the glycan ions. The relative amounts for 67 of the more abundant glycans are compared in **Figure 3**. Structural heterogeneity was greater for FSHβ glycans than for FSHα glycans. While 33 glycan structures accounted for 90% of the FSHβ glycan abundance (**Figure 3A**), only 13 FSHα glycan structures accounted for 90% of α-subunit glycan abundance (**Figure 3B**). The three most abundant FSHβ glycans were bi-antennary, tri-antennary, and tetra-antennary (**Table 1**, structures 26, 55, and 82, respectively). Structure 26 was found in low abundance at both FSHα glycosylation sites, however, a related structure 22, which is virtually the same as 26, lacking only core fucose, was the most abundant glycan released from Asn^78^ and second most abundant Asn^52^ glycan. Structure 55 was absent from both FSHα glycosylation sites, however, a nearly identical, non-fucosylated, tri-antennary structure 49 was second and third most abundant Asn^52^ and Asn^78^ glycan, respectively. Tetra-antennary structure 82 was also missing from FSHα glycans. The fourth most abundant FSHβ glycan, partially sialylated, di-Neu5Ac, tri-antennary, core-fucosylated structure 54, was high in abundance in FSHβ and low abundance in FSHα. The otherwise identical, non-fucosylated structure 48 was abundant in both FSHα glycan populations and low in abundance in FSHβ. Glycans possessing a bisecting GlcNAc residue exhibited a similar pattern of relative abundance with fucosylated, fully sialylated bi-antennary structure 46, tri-antennary structure 75 and disialylated tri-antennary structure 74 highly abundant in FSHβ, yet absent (35) or low in abundance in FSHα, while the non-fucosylated counterparts (structures 43, 72, and 71, respectively) exhibited the opposite pattern of relative abundance, high abundance in FSHα, but low in FSHβ. One FSHα glycan stood out, structure 34, which was bi-antennary with one GalNAc and one Gal residue, each capped with Neu5Ac. This structure accounted for 20% of αAsn^78^ and 10% of αAsn^52^ glycans (**Figure 3B**) and probably accounted for bi-antennary glycan abundance being greater than tri-antennary abundance in the αAsn^78^ population (**Figure 3E**). The alternative structure with a bisecting GlcNAc residue and a single branch incorporating Gal would have had to accommodate two Neu5Ac residues.

With regard to glycan structure types, over 83-85% of FSHβ glycans were core fucosylated, while less than 6-7% of FSHα glycans possessed core fucose (**Figure 3C**). FSH glycans included 0.5-1.1% oligomannose or hybrid type structures on the α-subunit at both glycosylation sites and 0.15-0.27% in the FSHβ glycans (**Figure 3D**). The patterns of glycan branching also differed between subunits, with 24kDa- and 21kDa-FSHβ possessing 28% or 30% bi-antennary glycans, respectively, while 60% and 41% of these glycans decorated FSHα sites Asn^78^ and Asn^52^, respectively (**Figure 3E**). Tri-antennary glycans were more abundant in 24kDa-FSHβ and at αAsn^52^ (34% and 53%, respectively) than in 21kDa-FSHβ (29%) and at αAsn^78^ (36%, **Figure 3F**). Tetra-antennary glycans were largely restricted to FSHβ, with 32% on both 24kDa- and 21kDa-FSHβ as compared with only 3% or 6% on αAsn^78^ and αAsn^52^, respectively (**Figure 3G**). Moreover, as will be shown below, glycans the size of tetra-antennary oligosaccharides derived from αAsn^52^ on a different human pituitary FSHα preparation were tri-antennary, with lactosamine repeats providing the additional mass. Bisecting GlcNAc residues were found in 21-23% of FSHβ glycans, 29% of αAsn^78^ and 39% of αAsn^52^ glycans (**Figure 3H**). GalNAc residues substituting for Gal, particularly in α1-3Man complex branches were found in 4% of 24kDa-FSHβ, 7% of 21kDa-FSHβ, 17% of αAsn^78^ and 14% of αAsn^52^ glycans (**Figure 3I**). The GalNAc abundance was greater than the 3%, 6%, 9%, and 7% sulfate abundance, respectively, consistent with significant sialylation of GalNAc residues. The four most abundant FSHβ glycans, 26, 55, 82, and 54 were more abundant in the fully-glycosylated 24kDa-FSHβ, while glycans 45, 91, 58, 36, and 56 were much more abundant in 21kDa-FSHβ. The remainder exhibited essentially the same relative abundance. For FSHα glycans, 22 and 34 were more abundant in the Asn^78^ glycan population, while glycans 49, 48, 72, 71, 87, and 77 were more abundant in the Asn^52^ population.

### FSH Glycoform Fractionation

**Figure 4:**
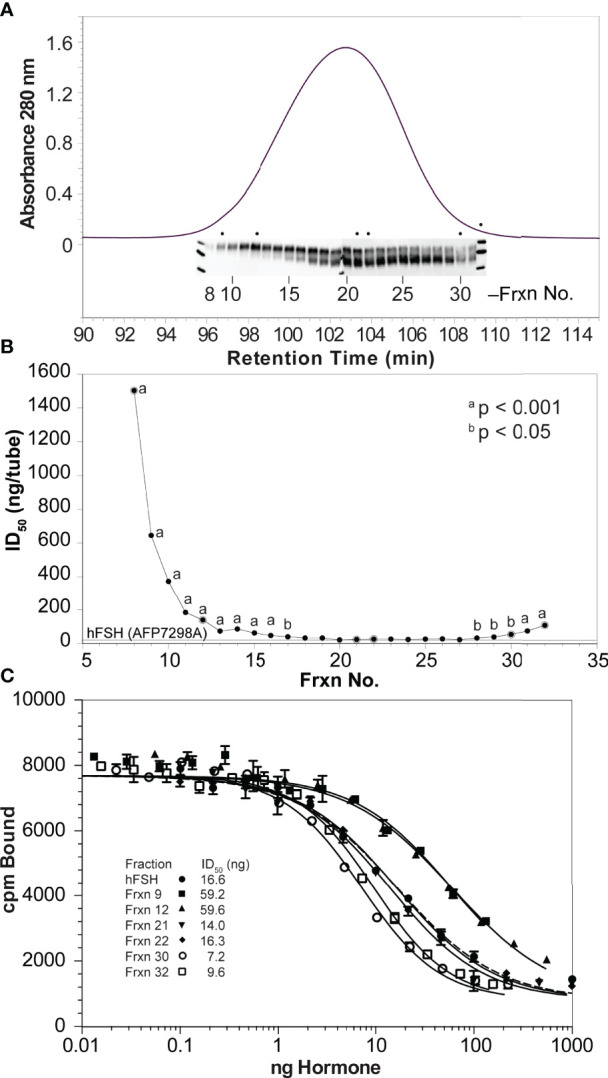
High resolution gel filtration chromatography of immunopurified hFSH. **(A)** The chromatogram shows the results of fractionating 10 mg immunopurified hFSH using three, 1 x 30 cm Superdex 75 columns in series. The inset shows the 24k-FSHβ and 21k-FSHβ bands in FSHβ Western blots performed on samples applied to two 15% polyacrylamide mini-gels. The dots indicate extensively analyzed fractions 9, 12, 21, 22, 30, and 32. **(B)** To screen the FSH fractions, ten µg samples of each were serially diluted 1:10 four times and FSH receptor binding activities compared. A general trend of increasing FSHR binding activity was noted going from fraction 8 to fraction 32. **(C)** Six fractions were selected to represent FSH24, the FSH24/FSH21 mixture, and FSH21. Ten-µg samples (based on SEC determination) were serially diluted and tested for FSH binding in the FSH radioligand assay.

High resolution Superdex 75 gel filtration remains the most effective method for naturally occurring FSH glycoform separation. The chromatogram for immunoaffinity-purified human pituitary hFSH consisted of a single protein peak (**Figure 4A**). This peak was subdivided into 25 fractions and the protein in each fraction quantified by UPLC size exclusion chromatography (not shown). Western blot analysis of 1 µg samples from 24 fractions (each blot accommodated 12 samples) indicated FSH24 was present in fractions 8-13 while predominantly FSH18/21 was found in fractions 26-31. Fractions 14-25 were mixtures of all 3 glycoforms. Although the sample loads, based on peak area, were the same in all cases, intensities of the FSHβ immunoreactive bands corresponding to fractions 8-10 and 30-31 were significantly lower than those for the rest of the fractions.

Radioligand assay of serially 1:10-diluted samples of each fraction confirmed the presence of binding competent FSH heterodimer (**Figure 4B**). The significantly greater ID_50_ values for fractions 8-15 and 30-32 were consistent with reduced FSHβ immunoactivity in the Western blots of these fractions, although the range was reduced for the second group of fractions, reflecting greater receptor-binding activities. Representative Superdex 75 fractions 9, 12, 21, 22, 30, and 32 were selected for additional characterization. The protein content of each of these samples was established by amino acid analysis of 5 µg samples. RLA based on adjusted protein content revealed a 7-fold range of average ID_50_ values between the two most active fractions, 30 and 32, as compared with the two least active fractions, 9 and 12 (**Figure 4C** and **Table 2**).

**Table 2:** FSH receptor-binding activities of hFSH glycoform fractions.

FSH Frxn.	ID_50_ (ng)	FSH Relative Potency (%)	FSH Relative Potency (IU/mg)	FSH21-FSH24-fold difference
hFSH*	16.6	100	8560	vs Frxn. 9
Fraction 9	59.2	28	2400	1
Fraction 12	59.6	28	2384	1.0
Fraction 21	14.0	119	10150	4.2
Fraction 22	16.3	101	9416	3.9
Fraction 30	7.2	229	19602	8.2
Fraction 32	9.6	173	14802	6.2

*hFSH reference preparation AFP9872A, 8560 IU/mg.

### Selective FSHα Asn^52^ Glycan Removal With PNGaseF

**Figure 5:**
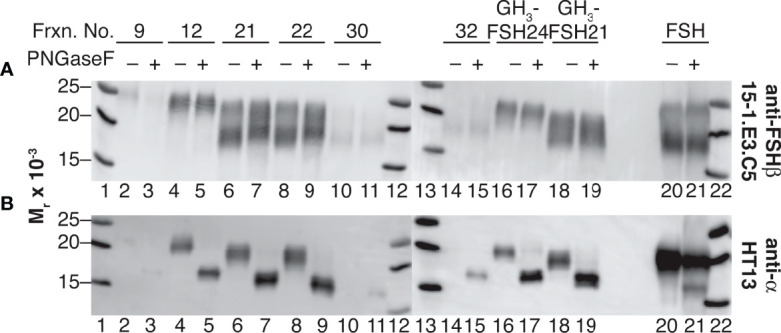
Selective αAsn^52^ deglycosylation of dissociated hFSH samples by PNGaseF digestion. Subunit mobilities were assessed by subunit-specific Western blots of 1 µg samples of FSH dissociated into subunits and transferred to 0.2 M ammonium bicarbonate buffer by ultrafiltration (see Methods). **(A)** FSHβ probed with anti-FSHβ monoclonal antibody 15-1.E3.E5 diluted 1:500. **(B)** FSHα probed with anti-α subunit monoclonal antibody HT13, diluted 1:5000. Lane 1, MW marker; lane 2, fraction 9 subunits; lane 3, fraction 9 subunits following PNGaseF digestion; lane 4, fraction 12 subunits; lane 5, fraction 12 subunits + PNGaseF; lane 6 subunits, fraction 21 subunits; lane 7, fraction 21 subunits + PNGaseF; lane 8, fraction 22 subunits; lane 9, fraction 22 subunits + PNGaseF; lane 10, fraction 30 subunits; lane 11, fraction 30 subunits + PNGaseF; lane 12, MW marker, lane 13, MW marker; lane 14, fraction 32 subunits; lane 15, fraction 32 subunits + PNGaseF; lane 16, recombinant GH_3_-FSH24 subunits; lane 17, GH_3_-FSH24 subunits + PNGaseF; lane 18,GH_3_-FSH21 subunits; lane 19, GH_3_-FSH21 subunits + PNGaseF; lane 20, intact hFSH; lane 23, intact hFSH + PNGaseF; lane 24, BioRad MW marker.

Western blot analysis of PNGaseF-digested, dissociated hFSH glycoform fractions 9, 12, 21, 22, 30, and 32 is shown in **Figure 5**. Consistent with earlier studies involving LH preparations (7), PNGaseF digestion did not affect FSHβ glycosylation in either dissociated or intact FSH (**Figure 5A**). In contrast, the mobilities of the dissociated FSHα subunits increased following PNGaseF digestion (**Figure 5B**). To evaluate the generality of this procedure for FSH preparations used in our studies, samples of recombinant GH_3_-hFSH glycoforms were dissociated and the subunits with 6 M GuHCl and the subunits subjected to the same mild PNGaseF deglycosylation procedure. These preparations exhibited similar patterns of FSHβ glycan resistance and selective PNGaseF deglycosylation (lanes 16-19). Limited sensitivity of αAsn^52^ N-glycans to PNGaseF digestion was exhibited by the intact hFSH sample, in which most of the FSHα remained fully glycosylated (lane 21). This demonstrated the need for subunit dissociation prior to PNGaseF digestion. As the protein amounts were based on SEC quantitation, reduced immunoactivity was observed for fractions 9, 30, and 32. Nevertheless, unaltered FSHβ and altered FSHα subunit band mobilities were observed in these samples as in the more abundant samples.

### FSHα Asn^52^ Glycan Size Trends as a Function of FSH Glycoform Size

**Figure 6:**
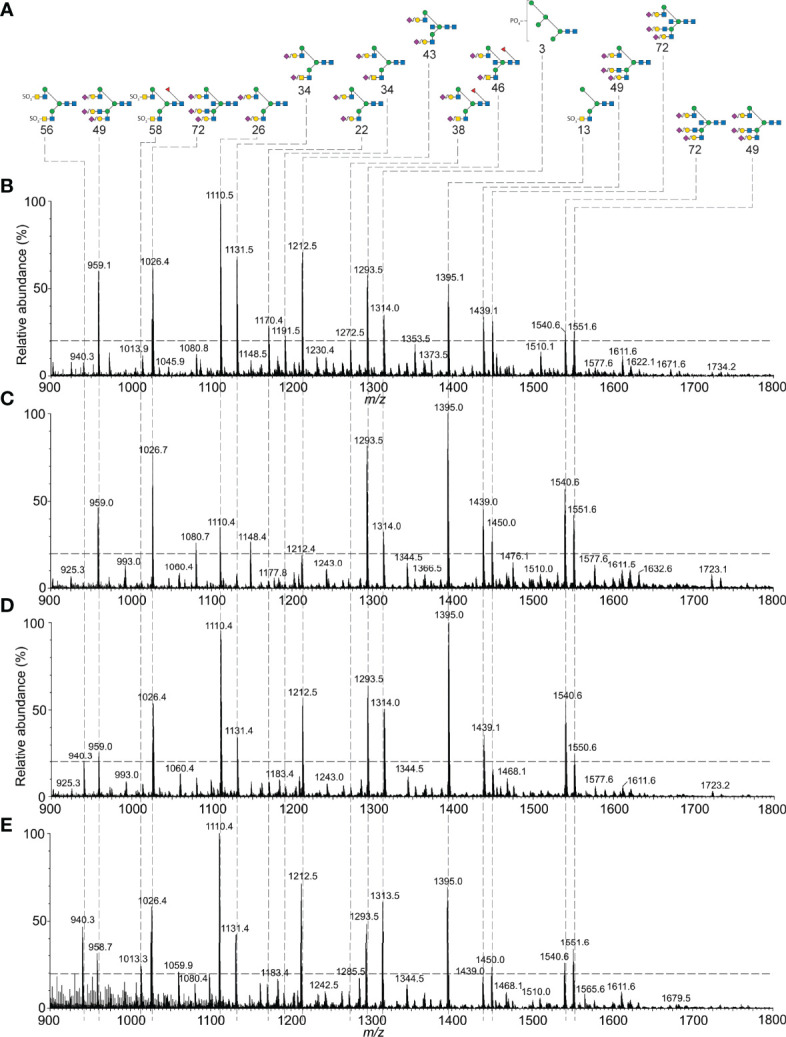
Mass spectrometry of hFSH glycoform aAsn52 glycans. Oligosaccharides were released from dissociated subunits by PNGaseF digestion (**Figure 5**). Nano-ESI-ion mobility-MS was used to characterize representative glycan samples. **(A)** FSHa Asn52 glycan structure diagrams. **(B)** FSHa Asn52 glycans isolated from FSHa in **Figure 1**. Nano-ESI spectrum from **Supplement Figure S1D**. **(C)** FSHa Asn52 glycans from pituitary FSH24 glycoform fraction 12 (Figure S5B). **(D)** FSHa Asn52 glycans from FSH24/21/18 fraction 21 (**Figure S5C**). **(E)** FSHa Asn52 glycans from FSH18/21 fraction 30 (**Figure S5E**).

**Figure 7:**
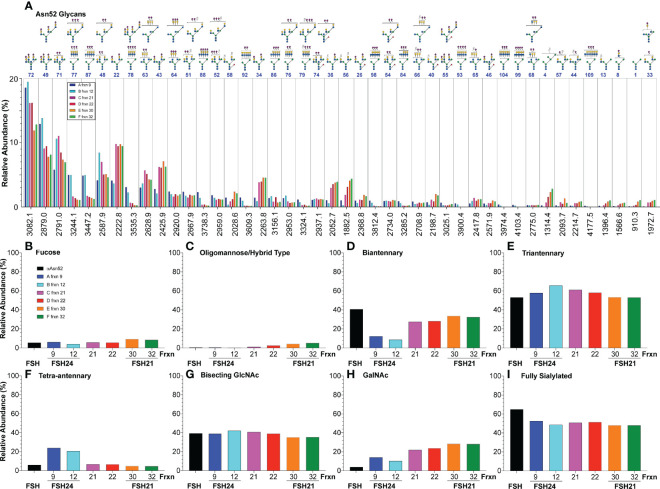
Relative abundance of hFSH glycoform αAsn^52^ oligosaccharide populations. FSH indicates FSHα Asn^52^ glycan data (black bars) from **Table 1** for comparison of products of conventional pituitary FSH purification with immunoaffinity purification. Fractions 9, 12, 21, 22, 30, and 32 are those recovered from the Superdex 75 chromatogram in **Figure 4A**. **(A)** Forty-four of 82 glycans ranked in order of fraction 9 glycan abundance. **(B)** Fraction of FSHα Asn^52^ glycans possessing core fucose. **(C)** Oligomannose or hybrid type glycan abundance. **(D)** Biantennary glycan abundance. **(E)** Triantennary glycan abundance. **(F)** Tetra-antennary glycan abundance. **(G)** Glycans possessing bisecting GlcNAc residue. **(H)** Glycans possessing GalNAc residue substitution for Gal in complex antennae. **(I)** Glycans possessing sufficient Neu5Ac to cap all complex branches.

We characterized αAsn^52^ N-glycans from all six glycoform fractions (**Supplement Figures S6**-**S8** and **Tables S15**-**S18**). Representative spectra shown in **Figure 6** indicate very similar glycan populations in the largest and smallest FSH gel filtration fractions. Quantitative results for 44 of 65 glycans exhibiting a relative abundance >1% are plotted in **Figure 7A**. Three patterns of abundance were noted. Tri-antennary glycans 72, 49, 71, and 48, exhibited the highest abundance in fraction 9 and progressively decreased to the lowest abundance in fraction 30 or 32. Bi-antennary glycans, such as 22, 43, 34, 36, and 56, were lowest in abundance in fractions 9 and 12 and highest in the rest. The largest glycans, 77, 87, 78, 88, 92, 86, 76, 79, 98, 93, 104, 99 and 109, with *m/z* values suggesting tetra-antennary were most abundant in fractions 9 and 12, but low in the 4 remaining fractions. Glycan relative abundance was essentially the same in all fractions for structures 64, 51, 52, 74, and 54. Overall, tri-antennary were the most abundant type in all FSH glycoform samples analyzed (**Figure 7E**), while the tetra-antennary were only enriched in FSH24 fractions 9 and 12 (**Figure 7F**). Glycans found to be more abundant in fractions 21, 22, 30, and 32 were typically bi-antennary, such as structures 22, 43, 34, 36, and 56 (**Figure 7A**). However, tri-antennary glycans were the most abundant type of αAsn^52^ glycan found in all FSH samples evaluated (**Figure 7E**). As these glycans were all derived from the FSHα subunit, core fucose glycan abundance was low (**Figure 7B**). Almost 40% of these glycans possessed a bisecting GlcNAc residue and there was no size-associated difference in their distribution. GalNAc substitution for Gal was observed in most FSH glycoform fractions with a trend toward increasing abundance with decreasing molecular size of FSH (**Figure 7H**). About 50% of immunopurified FSH glycans were fully-sialylated. Fully-sialylated glycans were more abundant in the pituitary hFSH preparation AFP7298A, consistent with anion exchange chromatography enrichment of negatively charged glycans (63).

### Sialic Acid Linkages Associated With αAsn^52^ Glycans

ESI-MS/MS was used to define the desialylated glycans recovered from FSH preparation AFP4161 FSHα subunit Asn^52^ glycosylation site (**Supplement Figure S9** and **Table S19**). Structures found during analysis of FSH preparation AFP7298A αAsn^52^ were largely confirmed (**Supplement Figures S9A–M**). However, for glycans with masses consistent with tetra-antennary oligosaccharides, tri-antennary oligosaccharides with lactosamine repeats were encountered instead (**Supplement Figures S9N–O**).

**Figure 8:**
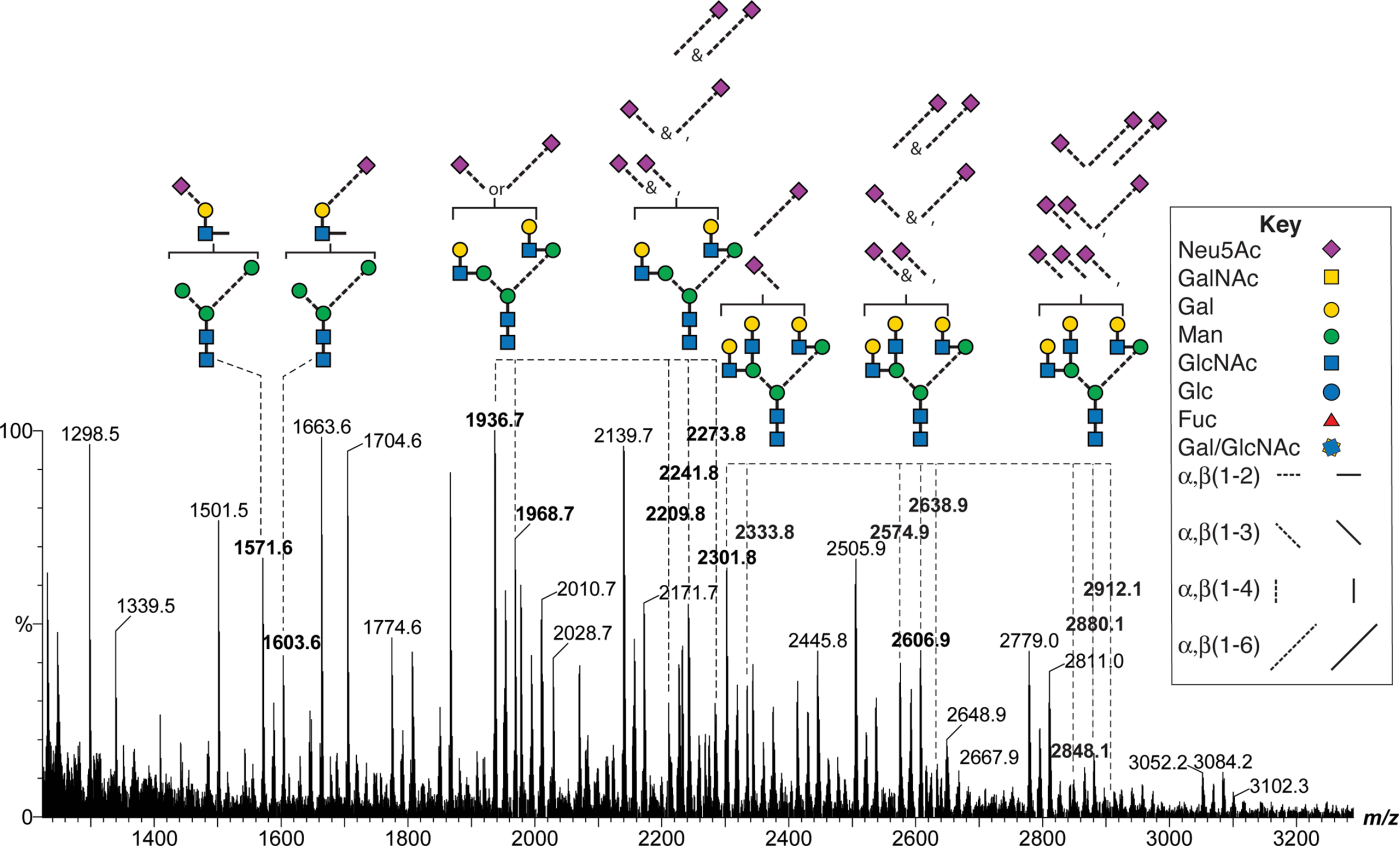
MALDI-MS analysis of DMT-MM-derivatized hFSH αAsn^52^ oligosaccharides. Representative mono-, di-, and tri-antennary glycans showing patterns of α2-3 and α2-6 linked Neu5Ac residues for the mono-, di- and tri-sialylated variants of the neutral glycan structures. The *m/z* values for selected oligosaccharide ions are shown in bold.

**Figure 9:**
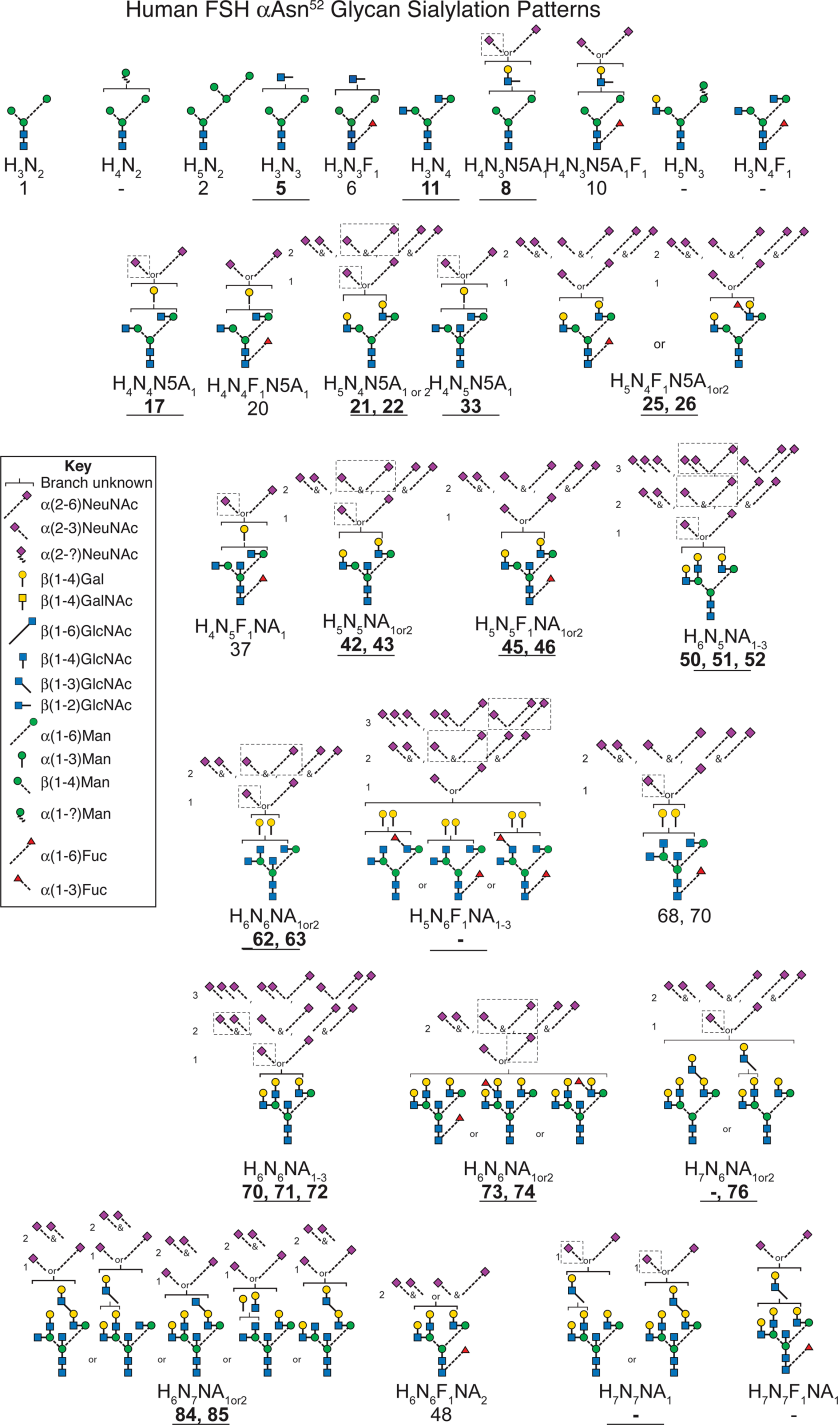
Patterns of sialic acid heterogeneity in the FSHα Asn^52^ glycan population. The neutral core glycan structure(s) consistent with composition and CID-MS (bold text, underlined, see **Supplement Figures S9A, O**). Sialic acid distributions based on data derived from **Figure 7** and **Table S19**. Major sialylation patterns indicated by dashed boxes. Glycan masses from **Tables 1** and **3**. Glycan structures identified according to **Table 1**. A dash indicates no corresponding glycan in the table.

Neu5Ac is connected to FSH glycans by both α2-3 and α2-6 linkages (64). An earlier study of FSHα Asn^52^ glycans derived from a different hFSH preparation, AFP4161, included modification with DMT-MM. This stabilized Neu5Ac to MS analysis and a 32 mass unit difference distinguished α2-3 from α2-6 linkages (**Figure 8** and **Supplement Table S19**). Glycans with single complex branches yielded two sialylated ion species, one linked α2-3 and the other linked α2-6. Bi-antennary glycans yielded 5 ions, two for the mono-sialylated variants and three for the combinations of di-sialylated variants: both α2-3 linked Neu5Ac, one α2-3 and one α2-6 linked Neu5Ac, or both α2-6 linked Neu5Ac. Tri-antennary glycans yielded 8 variants, the mono- and di-sialylated patterns described for bi-antennary glycans along with three additional tri-sialylated glycan variations, three α2-3 linked Neu5Ac residues, two α2-3 and one α2-6 linked Neu5Ac residues, and one α2-3 linked with two α2-6 linked Neu5Ac residues. No tri-antennary glycans were observed possessing three α2-6 linked Neu5Ac residues (**Figure 9**). It was notable that no tetra-antennary glycans were confirmed by fragmentation of 44% of the pituitary hFSH glycans. Rather, lactosamine repeats were observed with the repeats on branches other than the textbook Man6-GlcNAc6 branch (65). This contrasts with earlier studies involving glycans from all 4 FSH N-glycosylation sites, which largely detected tetra-antennary glycans in the tetra-antennary glycan mass population. Pituitary FSH glycans were concluded to be tetra-antennary, however, the abundance of the glycans with tetra-antennary mass is low in FSHα. Accordingly, these may not have been observed in studies involving total FSH glycans, which would be dominated by FSHβ tetra-antennary glycans. Whether tri-antennary glycans with lactosamine repeats are restricted to αAsn^52^ is a question for future studies.

### Pituitary FSH in Serum and Tissue

**Figure 10:**
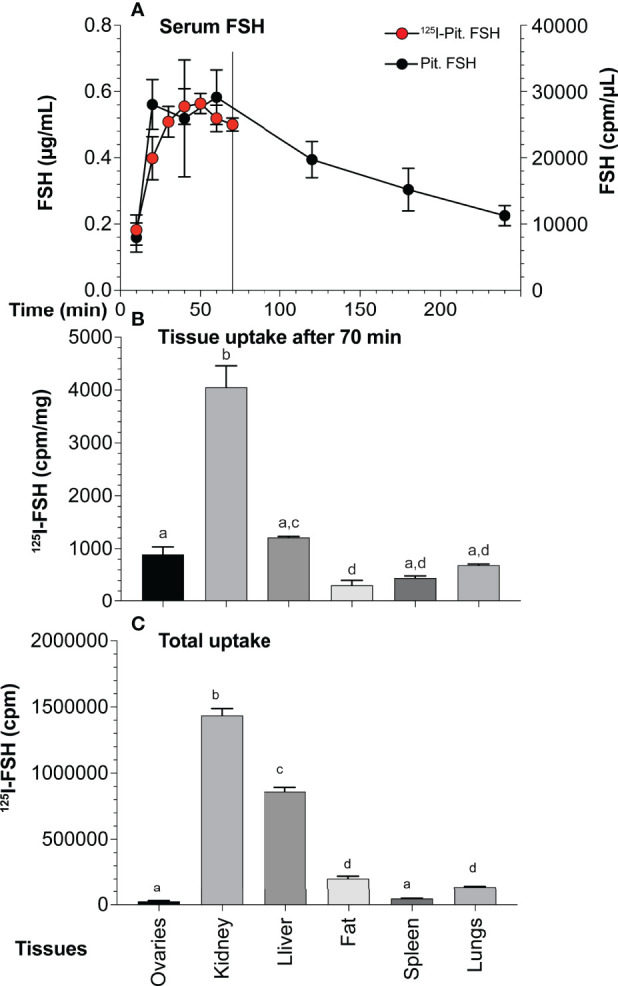
Pituitary FSH uptake and tissue accumulation. **(A)** FSH appearance and clearance from mouse serum following IP injection of 10 µg unlabeled pituitary FSH over 6 hr (solid circles, mean ± SD, n = 5) or 5 ng ^125^I-pituitary FSH over 70 min (red circles, mean ± SD, n = 3). The points are mean values of three mice The line indicates 70 min point at which mice were euthanized for tissue recovery. **(B)** Normalized tissue uptake of ^125^I-pituitary FSH 70 min following IP injection of 1 µg pituitary FSH tracer. Significant differences (p < 0.05) indicated by different letters; a ovaries, b kidney, c liver, and d fat. **(C)** Total ^125^I-FSH uptake in each tissue showing impact of size.

The pattern of pituitary FSH uptake and clearance was determined following IP injection of 10 µg unlabeled pituitary hFSH. Serum sample ELISA revealed FSH concentrations reached a maximum value at 20 min and remained elevated for another 40 min before beginning to decline (**Figure 10A**). Serum ^125^I-pituitary FSH following IP injection of 1 µg tracer rose more gradually, reaching peak accumulation at 40-50 min, then decreasing. The differences in serum uptake between the labeled and unlabeled FSH may represent dilution errors needed to measure serum FSH in the ELISA vs direct measurement of ^125^I in serum samples. The use of 10-fold more hormone in the unlabeled experiment likely contributed to the faster rise in serum hFSH.

Seventy min after injection ^125^I-FSH accumulation, regardless of whether total cpm taken up or normalized to cpm per mg tissue, was highest in the kidney (**Figures 10B, C**), consistent with its major role in FSH clearance (66). Liver uptake was second highest, followed by fat and lung uptake. Total ovarian uptake was relatively low due to its small size. When normalized for tissue mass, the kidney remained the site of highest FSH tracer uptake. Liver uptake was second, but not significantly higher than that of the ovary. Both were higher than fat and spleen. Accumulation in lungs was not significantly different than in the ovaries, although less than that in the liver.

## Discussion

Mass spectrometry of FSH proteinase K glycopeptides provided the first glycosylation site-specific characterization of pituitary FSH (10) and FSH isoform (11) oligosaccharides. Eleven oligosaccharides were identified at each of two glycosylation sites, αAsn^52^ and βAsn^24^, (**Table 3**), while only 4 were identified at βAsn^7^, and two at αAsn^78^. The deficiency in Asn^78^ glycans was largely due to the absence of the most abundant glycopeptide, -Asn^78^-His-Thr-. Mass spectrometry of PNGaseF-released oligosaccharides separated from several denatured FSH preparations revealed >30 to almost 100 glycan structures (2, 9). While this made it obvious the glycopeptide study had underestimated glycan heterogeneity in pituitary FSH, the extent was unknown. The sequential PNGaseF digestion protocol developed for LH and CG α-subunits (52) released FSHα glycans from Asn^52^, then Asn^78^. Both Asn^7^ and Asn^24^ glycan populations were resistant to PNGaseF digestion in native, FSHβ (**Figure 5**), while PNGaseF released both from reduced and alkylated 24kDa-FSHβ. As only 21kDa-FSHβ was detected in the hypo-glycosylated FSHβ preparation, the Asn^7^ glycan population was defined for this variant. Nano-ESI-ion mobility-MS analysis revealed populations of 45-61 glycans at each glycosylation site, suggesting glycopeptide analysis had only detected more abundant glycans. Indeed, all Asn^7^, 8 of 11 Asn^52^, and 10 of 11 Asn^24^ glycans encountered in the glycopeptide studies exhibited relative abundances of >1% when characterized as oligosaccharides (**Figure 3**, arrows). However, not all the major glycans in the oligosaccharide populations, such as structures 75, 90, 81, 95, and 96, were detected in the glycopeptide analysis, while fairly rare glycans, such as structures 21 and 33, were observed. Overall, oligosaccharide mass spectrometry identified four times as many FSH glycans as glycopeptide mass spectrometry. This most likely reflects the suppressive effect of the peptide moieties on ionization. Gel filtration partially eliminated peptide inhibition that prevented analysis of unfractionated proteinase K digests (10). However, no Asn^78^-His-Thr glycopeptides were detected during analysis of FSH, LH, TSH, or hCG glycopeptide preparations, despite it being the most abundant product of proteinase K digestion of the αAsn^78^ glycosylation site (10, 11, 68).

**Table 3:** Oligosaccharides detected at FSH glycosylation sites in site-specific glycosylation studies.

αAsn^52^	αAsn^78^	βAsn^7^	βAsn^24^	FSH Source
11	2	4	11	Pituitary (10, 11)
5-6	4-5	17	6-7	CHO cell (13)
7	6	21	7	CHO cell (13)
5-6	6-7	26-27	13	CHO cell (12)
7	8	25	13	CHO cell (12)
10	20	16	25	Urinary (14)
18	15	26	22	CHO cell (14)
1	1	ND	5	Serum (67)
0-1	0-2	1-3	6-8	rec-hFSH serum (67)
45	49	61	–	Pituitary (**Table 1**)

ND, not detected.

Both FSHα glycosylation sites possess the same glycan populations, differing only in the relative abundance of particular glycan types. For example, Asn^78^ possesses a greater abundance of bi-antennary glycans, while Asn^52^ possesses a greater abundance of tri-antennary glycans. As Asn^52^ glycans are close to both FSHβ glycans, the more extensive branching may reflect greater exposure to GlcNAc transferase IV, which adds a β1-4-linked GlcNAc residue to the Man3 branch, while Asn^78^ glycans may experience reduced exposure to this transferase due to their location at the opposite end of the molecule. The glycan populations at both FSHβ glycosylation sites are also similar to each other in that the order of abundance was almost identical for the top 9 structures, although structures 55 and 82 exchanged positions with each other in the order of 21kDa-FSHβ glycan abundance as did structures 54 and 46. Many of the glycans exhibited the same relative abundance despite representing Asn^7^ only or both glycosylation sites.

Restriction of core fucosylation primarily to β-subunit oligosaccharides has been reported for other glycoprotein hormones (69–71). Asn^78^ glycans appear less accessible in folded FSHα as indicated by limited PNGaseF sensitivity in folded α-subunit (7). One could argue these glycans are less accessible to Golgi FUT8 because only folded proteins enter this compartment. However, Asn^52^ glycans become much less accessible in the heterodimer despite being located on an enzyme accessible loop (**Figure 5**). By the same token, FSHβ Asn^7^ and Asn^24^ glycans are PNGaseF resistant in both native hormone as well as isolated subunit. All insect cell-expressed FSH glycans were susceptible to endoglycosidase F-1 digestion (43, 44), indicating the region of the first glycosidic bond was enzyme accessible and suggesting the nearby reducing terminal GlcNAc C6 hydroxyl group could be accessible to fucosyltransferase.

In pituitary hFSH, tri-antennary and bi-antennary glycans predominated at the αAsn^52^ glycosylation site (**Figures 3E, F**), but this α-subunit preparation was derived from largely FSH24. High-resolution gel filtration had little effect on the size distribution of the two most abundant oligosaccharide types in the Asn^52^ glycan population (**Figure 7**). These results support the hypothesis that FSHα oligosaccharides have limited impact on FSH heterodimer size (5). FSHβ oligosaccharides increase the width of the elliptical FSH heterodimer substantially, while αAsn^52^ glycan size extends FSH length, which is ~75 Å for the peptide moiety alone, making it large enough to influence ultrafiltration in the kidney (72). Consistent with its major role in FSH clearance, the kidney accumulated the greatest amount of tissue-associated ^125^I-FSH (**Figure 10**). The liver accounted for the second highest tissue accumulation, probably due to its large size and the asialoglycoprotein receptor binding α2-6-Neu5Ac-terminated oligosaccharides (73), which are abundant in hFSH (64). Oligosaccharides with a single α2-6-linked Neu5Ac tended to be the most abundant pattern for FSH αAsn^52^ oligosaccharides. If that pattern is typical of FSHβ glycosylation, then clearance by the liver is probably low, since single accessible Gal residue glycans exhibit the lowest affinity for the asialoglycoprotein receptor (74).

We reported very small, 4-residue, oligosaccharides attached to horse pituitary LHα Asn^56^ (homologous to human αAsn^52^) (75). As hypo-glycosylated FSHα subunits exhibited greater electrophoretic mobility during SDS-PAGE (6) as well as exhibiting 2- to 3-fold greater binding to FSHR than FSH24 in saturation binding experiments (25), it was reasonable to entertain the hypothesis that small hypo-glycosylated FSHα Asn^52^ glycans were responsible for the increased receptor-binding, possibly because more than one FSH with such small glycans could simultaneously fit in the putative FSHR trimers (76). However, no such small glycans were encountered in any of the three FSHα Asn^52^ glycan samples. Tri-antennary glycans proved the most abundant αAsn^52^ oligosaccharide type not only in the predominantly FSH24 pituitary hFSH, but also in the lowest molecular weight fractions of hypo-glycosylated pituitary FSH. Furthermore, even in the largest FSHα Asn^52^ glycan sample, lactosamine repeat-bearing tri-antennary glycans were revealed, rather than very small oligosaccharides. Thus, mass spectrometry data for FSHα Asn^52^ glycans fail to support the small glycan hypothesis.

**Figure 11:**
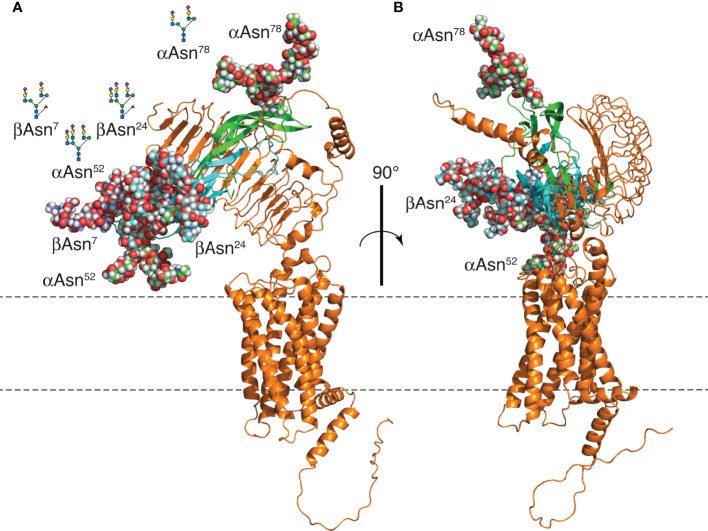
FSH24/FSHR model. **(A)** Models of FSH24 and FSHR associated based on the crystal structure of FSH-FSHR_ECD_ (4AY9). The FSHα subunit cartoon is shown in green and FSHβ in cyan. Oligosaccharides are rendered as spheres. FSHβ Asn^7^ glycan is colored dark blue to distinguish it from the cyan-colored Asn^24^ glycan. Each glycan represents the most abundant glycan structure at αAsn^52^, αAsn^78^, and βAsn^7^ (**Table 2**) with a tri-antennary glycan chosen for βAsn^24^. Three glycans are clustered at one end, while the αAsn^78^ glycan is at the opposite end. **(B)** Figure rotated 90° to emphasize the FSH peptide moiety is sandwiched between the FSH glycans and the FSHR. Oligosaccharide models created and attached to an FSH model extracted from pdb file 4AY9 using the web-based Glycam glycoprotein builder tool [Woods Group. (2005-2016) GLYCAM Web. Complex Carbohydrate Research Center, University of Georgia, Athens, GA. (http://glycam.org)].

Three FSH N-glycans affecting biological activity, αAsn^52^, βAsn^7^, and βAsn^24^ (6, 21, 24, 45), are clustered at one end of the FSH molecule (**Figure 11**), while the αAsn^78^ glycan, not implicated in FSHR activation (21, 24), is located at the opposite end. Elimination of the αAsn^52^ glycan site or either one of the FSHβ N-glycan sites, Asn^7^ or Asn^24^, resulted in increased FSHR occupancy in saturation binding studies (25, 45). Crystallographic studies of endoglycosidase F1-deglycosylated FSH/FSHR extracellular domain (ECD) lacking or including the hinge region, rationalized the requirement for an intact heterodimer to engage the receptor (43, 44) as well as the limited effect of FSH carbohydrate on FSHR affinity (19, 21, 24, 77) by exclusively protein-protein interactions between FSH and FSHR (43, 44). Oligosaccharide models added to FSHR-bound FSH using the Glycam web tools, show these are on the back side of FSH and off to each side of the receptor-binding site (**Figure 11**). Cryogenic electron microscopic (cryo-EM) structures for the related LH and CG receptor (LH/CGR) and TSH receptor (TSHR) revealed a rigid body extracellular domain rotation of 45° or 38°, respectively, between the inactive and active conformations (46, 47). These studies suggest the ligands for these receptors navigate a gap between the receptor ECD and the cell membrane. As the cluster of activity-related FSH N-glycans would face the cell membrane in order for FSH to engage the FSHR in these models, steric hindrance may affect receptor binding, with the αAsn^52^ oligosaccharide likely closest to the cell surface. Limited access to FSHR is consistent with the approximately 1-hr lag in FSH24 binding. However, FSH21/18, which possesses two of three glycans in the activity-related carbohydrate cluster, binds FSHR with little to no lag (6). Perhaps loss of one of these glycans permits greater flexibility, thereby reducing steric hindrance.

A mechanism involving FSH ligands engaging a sterically hindered FSHR binding site is more plausible than one invoking FSHR oligomerization. The report of FSHR_ECD_-FSH complex structures as potential dimers (43) was followed a few years later by evidence for intact FSHR oligomers at the cell surface (37). However, a study linking glycoprotein hormone oligomerization to negative cooperativity indicated most of these receptors were monomeric (39). PD-PALM studies involving both LH and FSH receptors have confirmed the largely monomeric nature of these receptors (42, 48). In addition to defining the pattern of FSHR oligomerization, FSH glycoforms altered oligomerization patterns and a biased FSH agonist increased FSHR oligomerization from 30% to 50% in 15 min. Perhaps part of the bias of crystallographic studies toward dimeric or trimeric FSH/FSHR structures stems from the need to deglycosylate FSH in order to obtain diffractable crystals. These inactive FSHR ligands promote receptor oligomerization. Cryo-EM studies are biased towards the monomeric receptor, as single particle studies involve monomeric receptors. The latter approach does have the advantage of permitting the inclusion of glycosylated glycoprotein hormones and might provide insight into FSHR activation by FSH glycoforms bearing variable patterns of 3-4 N-glycans.

## Data Availability Statement

The datasets presented in this study can be found in online repositories. The names of the repository/repositories and accession number(s) can be found in the article/**Supplementary Material**.

## Ethics Statement

The animal study was reviewed and approved by Wichita State University IACUC.

## Author Contributions

GB purified oligosaccharides, wrote the manuscript, edited the manuscript. JM cultured cells used in binding assays, edited manuscript. AB prepared monoclonal antibodies used in Western blots, edited manuscript. TS performed FSH binding assays, edited manuscript. VladimirYB iodinated tracers, performed confirming binding assays, edited manuscript. ViktorYB purified hFSH, purified hFSH subunit glycoforms, performed Western blots, edited manuscript. WW performed Superdex 75 and SEC chromatography’s, edited manuscript. DH performed all mass spectrometry experiments, prepared all MS tables, edited manuscript. All authors contributed to the article and approved the submitted version.

## Funding

Research reported in this publication was supported by the National Institute On Aging of the National Institutes of Health under Award Number P01AG029531 with additional support from NIH grants G20 RR031092, and P20 GM103418. The content is solely the responsibility of the authors and does not necessarily represent the official views of the National Institutes of Health.

## Conflict of Interest

The authors declare that the research was conducted in the absence of any commercial or financial relationships that could be construed as a potential conflict of interest.

## Publisher’s Note

All claims expressed in this article are solely those of the authors and do not necessarily represent those of their affiliated organizations, or those of the publisher, the editors and the reviewers. Any product that may be evaluated in this article, or claim that may be made by its manufacturer, is not guaranteed or endorsed by the publisher.

## References

[1] Bousfield GR Harvey DJ. Follicle-Stimulating Hormone Glycobiology. Endocrinology (2019) 160:1515–35. doi: 10.1210/en.2019-00001 PMC6534497 31127275

[2] Bousfield GR Butnev VY Rueda-Santos MA Brown A Smalter Hall A Harvey DJ. Macro and Micro Heterogeneity in Pituitary and Urinary Follicle-Stimulating Hormone Glycosylation. J Glycomics Lipidomics (2014) 4:125. doi: 10.4172/2153-0637.1000125 PMC4338580 25722940

[3] Renwick AGC Mizuochi T Kochibe N Kobata A. The Asparagine-Linked Sugar Chains of Human Follicle-Stimulating Hormone. J Biochem (1987) 101:1209–21. doi: 10.1093/oxfordjournals.jbchem.a121985 3115970

[4] Green ED Baenziger JU. Asparagine-Linked Oligosaccharides on Lutropin, Follitropin, and Thyrotropin: I. Structural Elucidation of the Sulfated and Sialylated Oligosaccharides on Bovine, Ovine and Human Pituitary Glycoprotein Hormones. J Biol Chem (1988) 263:25–35. doi: 10.1016/S0021-9258(19)57351-3 3121609

[5] Walton WJ Nguyen VT Butnev VY Singh V Moore WT Bousfield GR. Characterization of Human Follicle-Stimulating Hormone Isoforms Reveals a Non-Glycosylated β-Subunit in Addition to the Conventional Glycosylated β-Subunit. J Clin Endocrinol Metab (2001) 86:3675–85. doi: 10.1210/jcem.86.8.7712 11502795

[6] Bousfield GR Butnev VY Butnev VY Hiromasa Y Harvey DJ May JV. Hypo-Glycosylated Human Follicle-Stimulating Hormone (Hfsh21/18) Is Much More Active *In Vitro* Than Fully-Glycosylated hFSH (Hfsh24). Mol Cell Endocrinol (2014) 382:989–97. doi: 10.1016/j.mce.2013.11.008 PMC3908837 24291635

[7] Gotschall RR Bousfield GR. Oligosaccharide Mapping Reveals Hormone-Specific Glycosylation Patterns on Equine Gonadotropin α-Subunit Asn^56^ . Endocrinology (1996) 137:2543–57. doi: 10.1210/endo.137.6.8641208 8641208

[8] Ulloa-Aguirre A Midgley AR Jr. Beitins IZ Padmanbhan V. Follicle-Stimulating Isohormones: Characterization and Physiological Relevance. Endocr Rev (1995) 16:765–87. doi: 10.1210/edrv-16-6-765 8747835

[9] Bousfield GR Butnev VY White WK Smalter Hall A Harvey DJ. Comparison of Follicle-Stimulating Hormone Glycosylation Microheterogeneity by Quantitative Negative Mode Nano-Electrospray Mass Spectrometry of Peptide-N-Glycanase-Released Oligosaccharides. J Glycomics Lipidomics (2015) 5:129. doi: 10.4172/2153-0637.1000129 25960929 PMC4423619

[10] Dalpathado DS Irungu JA Go EP Butnev VY Norton K Bousfield GR. Comparative Glycomics of the Glycoprotein Hormone Follicle-Stimulating Hormone (FSH): Glycopeptide Analysis of Isolates From Two Mammalian Species. Biochemistry (2006) 45:8665–73. doi: 10.1021/bi060435k 16834341

[11] Bousfield GR Butnev VY Bidart JM Dalpathado D Irungu J Desaire H. Chromatofocusing Fails to Separate hFSH Isoforms on the Basis of Glycan Structure. Biochemistry (2008) 47:1708–20. doi: 10.1021/bi701764w 18197704

[12] Mastrangeli R Satwekar A Cutillo F Ciampolillo C Palinsky W Longobardi S. *In-Vivo* Biological Activity and Glycosylation Analysis of a Biosimilar Recombinant Human Follicle-Stimulating Hormone Product (Bemfola) Compared With Its Reference Medicinal Product (GONAL-F). PLoS One (2017) 12:e0184139. doi: 10.1371/journal.pone.0184139 28880909 PMC5589168

[13] Grass J Pabst M Chang M Wozny M Altmann F. Analysis of Recombinant Human Follicle-Stimulating Hormone (FSH) by Mass Spectrometric Approaches. Anal Bioanal Chem (2011) 400:2427–38. doi: 10.1007/s00216-011-4923-5 21461863

[14] Wang H Chen X Zhang X Zhang W Yan L Yin H. Comparative Assessment of Glycosylation of a Recombinant Human FSH and a Highly Purified FSH Extracted From Human Urine. J Proteome Res (2016) 15:923–32. doi: 10.1021/acs.jproteome.5b00921 26812091

[15] Singh A Kildegaard HF Andersen MR. An Online Compendium of CHO RNA-Seq Data Allows Identification of CHO Cell Line-Specific Transcriptomic Signatures. Biotechnol J (2018) 13:e1800070. doi: 10.1002/biot.201800070 29762913

[16] Sterer LM Park EI Townsend RR Baenziger JU. The Asialoglycoprotein Receptor Regulates Levels of Plasma Glycoproteins Terminating With Sialic Acid Alpha2,6-Galactose. J Biol Chem (2009) 284:3777–83. doi: 10.1074/jbc.M808689200 PMC2635048 19075021

[17] Bogdanove EM Nolin JM Campbell GT. Qualitative and Quantitative Gonad-Pituitary Feedback. Recent Prog Horm Res (1975) 31:567–626. doi: 10.1016/B978-0-12-571131-9.50019-0 812161

[18] Manjunath P Sairam MR Sairam J. Studies on Pituitary Follitropin. X. Biochemical, Receptor Binding and Immunological Properties of Deglycosylated Ovine Hormone. Mol Cell Endocrinol (1982) 28:125–38. doi: 10.1016/0303-7207(82)90026-0 6182043

[19] Calvo FO Keutmann HT Bergert ER Ryan RJ. Deglycosylated Human Follitropin: Characterization and Effects on Adenosine Cyclic 3’,5’-Phosphate Production in Porcine Granulosa Cells. Biochemistry (1986) 25:3938–43. doi: 10.1021/bi00361a030 3017410

[20] Galway AB Hsueh AJW Keene JL Yamoto M Fauser BCJM Boime I. *In Vitro* and *In Vivo* Bioactivity of Recombinant Human Follicle-Stimulating Hormone and Paritally Deglycosylated Variants Secreted by Transfected Eukaryotic Cell Lines. Endocrinology (1990) 127:93–100. doi: 10.1210/endo-127-1-93 2141816

[21] Bishop LA Robertson DM Cahir N Schofield PR. Specific Roles for the Asparagine–Linked Carbohydrate Residues of Recombinant Human Follicle Stimulating Hormone in Receptor Binding and Signal Transduction. J Mol Endocrinol (1994) 8:722–31. doi: 10.1210/mend.8.6.7935488 7935488

[22] Valove FM Finch C Anasti JN Froehlich J Flack MR. Receptor Binding and Signal Transduction Are Dissociable Functions Requiring Different Sites on Follicle-Stimulating Hormone. Endocrinology (1994) 135:2657–61. doi: 10.1210/endo.135.6.7988456 7988456

[23] Keene JL Nishimori K Galway AB Matzuk MM Hsueh AJW Boime I. Recombinant Deglycosylated Human FSH Is an Antagonist of Human FSH Action in Cultured Rat Granulosa Cells. Endocrine (1994) 2:175–80.

[24] Flack MR Froehlich J Bennet AP Anasti J Nisula BC. Site-Directed Mutagenesis Defines the Individual Roles of the Glycosylation Sites on Follicle-Stimulating Hormone. J Biol Chem (1994) 269:14015–20. doi: 10.1016/S0021-9258(17)36748-0 8188681

[25] Butnev VY Butnev VY May JV Shuai B Tran P White WK. Production, Purification, and Characterization of Recombinant hFSH Glycoforms for Functional Studies. Mol Cell Endocrinol (2015) 405:41–52. doi: 10.1016/j.mce.2015.01.026 PMC4378652 25661536

[26] Jiang C Hou X Wang C May JF Butnev VY Bousfield GR. Hypo-Glycosylated hFSH has Greater Bioactivity Than Fully-Glycosylated Recombinant hFSH in Human Granulosa Cells. J Clin Endocrinol Metab (2015) 100:E852–60. doi: 10.1210/jc.2015-1317 PMC4454802 25915568

[27] Zarinan T Butnev VY Gutierrez-Sagal R Maravillas-Montero JL Marinez-Luis I Mejia-Dominguez NR. *In Vitro* Impact of FSH Glycosylation Variants on FSH Receptor-Stimulated Signal Transduction and Functional Selectivity. J Endocr Soc (2020) 4:1–23. doi: 10.1210/jendso/bvaa019 PMC7175721 32342021

[28] Liang A Plewes MR Hua G Hou X Blum HR Przygrodzka E. Bioactivity of Recombinant hFSH Glycosylation Variants in Primary Cultures of Porcine Granulosa Cells. Mol Cell Endocrinol (2020) 514:110911. doi: 10.1016/j.mce.2020.110911 32553947 PMC7418035

[29] Simon LE Liu Z Bousfield GR Kumar TR Duncan FE. Recombinant FSH Glycoforms Are Bioactive in Mouse Preantral Ovarian Follicles. Reproduction (2019) 158:517–27. doi: 10.1530/REP-19-0392 PMC7047736 31600726

[30] Wang H May J Shuai B May JV Bousfield GR Kumar TR. Evaluation of *In Vivo* Bioactivities of Recombiant Hypo-(FSH^21/18^) and Fully- (FSH^24^) Glycosylated Human FSH Glycoforms in *Fshb* Null Mice. Mol Cell Endocrinol (2016) 437:224–36. doi: 10.1016/j.mce.2016.08.031 PMC5048586 27561202

[31] Hua G George J Clark KL Jonas KC Johnson GP Southekal S. Hypo-Glycosylated hFSH Drives Ovarian Follicular Development More Efficiently Than Fully-Glycosylated hFSH: Enhanced Transcription and PI3K and MAPK Signaling. Hum Reprod (2021) 36:1891–906. doi: 10.1093/humrep/deab135 PMC8213452 34059912

[32] Gera S Sant D Haider S Korkmaz F Kuo TC Mathew M. First-In-Class Humanized FSH Blocking Antibody Targets Bone and Fat. Proc Natl Acad Sci (2020) 117:28971–9. doi: 10.1073/pnas.2014588117 PMC7682550 33127753

[33] Sprengel R Braun T Nikolics K Segaloff DL Seeburg PH. The Testicular Receptor for Follicle Stimulating Hormone: Structure and Functional Expression of Cloned cDNA. Mol Endocrinol (1990) 4:525–30. doi: 10.1210/mend-4-4-525 2126341

[34] Branca AA Sluss PM Smith RA Reichert LEJ. The Subunit Structure of the Follitropin Receptor. Chemical Cross-Linking of the Solubilized Follitropin-Receptor Complex. J Biol Chem (1985) 260:9988–93. doi: 10.1016/S0021-9258(17)39200-1 2991288

[35] Shin J Ji TH. Composition of Cross-Linked 125I-Follitropin-Receptor Complexes. J Biol Chem (1985) 260:12822–7. doi: 10.1016/S0021-9258(17)38951-2 2413032

[36] Dattatreyamurty B Smith RA Zhang SB Santa-Coloma TA Reichert LEJ. The Size of the Mature Membrane Receptor for Follicle-Stimulating Hormone Is Larger Than That Predicted From Its cDNA. J Mol Endocrinol (1992) 9:115–21. doi: 10.1677/jme.0.0090115 1418382

[37] Thomas RM Nechamen CA Mazurkeiwicz JE Muda M Palmer S Dias JA. Follice-Stimulating Hormone Receptor Forms Oligomers and Shows Evidence of Carboxyl-Terminal Proteolytic Processing. Endocrinology (2007) 148:1987–95. doi: 10.1210/en.2006-1672 PMC3113408 17272391

[38] Guan R Wu XP Feng X Zhang M Hebert TW Segaloff DL. Structural Determinants Underlying Constitutive Dimerization of Unoccupied Human Follitropin Receptors. Cell Signal (2010) 22:247–56. doi: 10.1016/j.cellsig.2009.09.023 PMC2787667 19800402

[39] Urizar E Montanelli L Loy T Bonomi M Swillens S Gales C. Glycoprotein Hormone Receptors: Link Between Receptor Homodimerization and Negative Cooperativity. EMBO J (2005) 24:1954–64. doi: 10.1038/sj.emboj.7600686 PMC1142614 15889138

[40] Zoenen M Urizar E Swillens S Vassart G Costagliola S. Evidence for Activity-Regulated Hormone-Binding Cooperativity Across Glycoprotein Hormone Receptor Hormomers. Nat Commun (2012) 3:1007. doi: 10.1038/ncomms1991 22893131

[41] Cheng K-W. Properties of Follicle-Stimulating-Hormone Receptor in Cell Membranes of Bovine Testes. Biochem J (1975) 149:123–32. doi: 10.1042/bj1490123 PMC1165599 242318

[42] Agwuegbo UT Colley E Albert AP Butnev VY Bousfield GR Jonas KC. Differential FSH Glycosylation Modulates FSHR Oligomerization and Subsequent cAMP Signaling. Front Endocrinol (2021) 12:765727. doi: 10.3389/fendo.2021.765727 PMC8678890 34925235

[43] Fan QR Hendrickson WA. Structure of Human Follicle-Stimulating Hormone in Complex With Its Receptor. Nature (2005) 433:269–77. doi: 10.1038/nature03206 PMC5514322 15662415

[44] Jiang X Liu H Chen X Chen PH Fischer D Sriraman V. Structure of Follicle-Stimulating Hormone in Complex With the Entire Ectodomain of Its Receptor. Proc Natl Acad Sci (2012) 109:12491–6. doi: 10.1073/pnas.1206643109 PMC3411987 22802634

[45] Jiang X Fischer D Chen X McKenna SD Liu H Sriraman V. Evidence for Follicle-Stimulating Hormone Receptor as a Functional Trimer. J Biol Chem (2014) 289:14273–82. doi: 10.1074/jbc.M114.549592 PMC4022893 24692546

[46] Duan J Xu P Cheng X Mao C Croll T He X. Structures of Full-Length Glycoprotein Hormone Receptor Signalling Complexes. Nature (2021) 598:688–92. doi: 10.1038/s41586-021-03924-2 34552239

[47] Faust B Singh I Zhang K Hoppe N Pinto AFM Muftuoglu Y. Autoantibody and Hormone Activation of the Thyrotropin G Protein-Coupled Receptor. bioRxiv (2022). doi: 10.1101/2022.01.06.475289

[48] Jonas KC Fanelli F Huhtaniemi IT Hanyaloglu AC. Single Molecule Analysis of Functionally Asymmetric G Protein-Coupled Receptor (GPCR) Oligomers Reveals Diverse Spatial and Structural Assemblies. J Biol Chem (2015) 290:3875–92. doi: 10.1074/jbc.M114.622498 PMC4326798 25516594

[49] Jiang X Dias JA He X. Structural Biology of Glycoprotein Hormones and Their Receptors: Insights to Signaling. Mol Cell Endocrinol (2013) 382:424–51. doi: 10.1016/j.mce.2013.08.021 24001578

[50] Shome B Parlow AF Liu WK Nahm HS Wen T Ward DN. A Reevaluation of the Amino Acid Sequence of Human Follitropin β-Subunit. J Prot Chem (1988) 7:325–39. doi: 10.1007/BF01024882 3151250

[51] Bousfield GR Ward DN. Purification of Lutropin and Follitropin in High Yield From Horse Pituitary Glands. J Biol Chem (1984) 259:1911–21. doi: 10.1016/S0021-9258(17)43494-6 6420415

[52] Butnev VY Gotschall RR Baker VL Moore WT Bousfield GR. Negative Influence of O-Linked Oligosaccharides of High Molecular Weight Equine Chorionic Gonadotropin on Its Luteinizing Hormone and Follicle-Stimulating Hormone Receptor-Binding Activities. Endocrinology (1996) 137:2530–42. doi: 10.1210/endo.137.6.8641207 8641207

[53] Harvey DJ Crispin M Scanlan C Singer BB Lucka L Chang VT. Differentiation Between Isomeric Triantennary N-Linked Glycans by Negative Ion Tandem Mass Spectrometry and Confirmation of Glycans Containing Galactose Attached to the Bisecting (β1-4-GlcNAc) Residue in N-Glycans From IgG. Rapid Commun Mass Spectrom (2008) 22:1047–52. doi: 10.1002/rcm.3470 18327885

[54] Harvey DJ Royle L Radcliffe CM Rudd PM Dwek RA. Structural and Quantitative Analysis of N-Linked Glycans by Matrix-Assisted Laser Desorption Ionization and Negative Ion Nanospray Mass Spectrometry. Anal Biochem (2008) 376:44–60. doi: 10.1016/j.ab.2008.01.025 18294950

[55] Harvey DJ. Fragmentation of Negative Ions From Carbohydrates: Part 3. Fragmentation of Hybrid and Complex N-Linked Glycans. J Am Soc Mass Spectrom (2005) 16:647–59. doi: 10.1016/j.jasms.2005.01.006 15862766

[56] Harvey DJ. Fragmentation of Negative Ions From Carbohydrates: Part 2. Fragmentation of High-Mannose N-Linked Glycans. J Am Soc Mass Spectrom (2005) 16:631–46. doi: 10.1016/j.jasms.2005.01.005 15862765

[57] Harvey DJ. Fragmentation of Negative Ions From Carbohydrates: Part 1. Use of Nitrate and Other Anionic Adducts for the Production of Negative Ion Electrospray Spectra From N-Linked Carbohydrates. J Am Soc Mass Spectrom (2005) 16:622–30. doi: 10.1016/j.jasms.2005.01.004 15862764

[58] Wheeler SF Domann P Harvey DJ. Derivatizstion of Sialic Acids for Stabilization in Matrix-Assisted Laser Desorption/Ionization Mass Spectrometry and Concomitant Differentiation of α(2-3)- and α(2-6)-Isomers. Rapid Commun Mass Spectrom (2009) 23:303–12. doi: 10.1002/rcm.3867 19089860

[59] Bousfield GR Ward DN. Evidence for Two Folding Domains in Glycoprotein Hormone α Subunits. Endocrinology (1994) 135:624–35. doi: 10.1210/endo.135.2.7518386 7518386

[60] Bidart J-M Troalen F Bousfield GR Bohuon C Bellet D. Monoclonal Antibodies Directed to Human and Equine Chorionic Gonadotropins as Probes for the Topographic Analysis of Epitopes on the Human α-Subunit. Endocrinology (1989) 124:923–9. doi: 10.1210/endo-124-2-923 2463907

[61] Ward DN Bousfield GR Gordon WL H. Sugino. Chemistry of the Peptide Components of Glycoprotein Hormones. In: Keel BA Grotjan HE Jr., editors. Microheterogeneity of Glycoprotein Hormones. Boca Raton: CRC Press. (1989). p. 1–21.

[62] Varki A Cummings RD Aebi M Packer NH Seeberger PH Esko JD. Symbol Nomenclature for Graphical Representation of Glycans. Glycobiology (2015) 25:1323–4. doi: 10.1093/glycob/cwv091 PMC4643639 26543186

[63] Reichert LE Jr. Parlow AF. Partial Purification and Separation of Human Pituitary Gonadotrophins. Endocinology (1964) 74:236–43. doi: 10.1210/endo-74-2-236 14122530

[64] Green ED Baenziger JU. Asparagine-Linked Oligosaccharides on Lutropin, Follitropin, and Thyrotropin II. Distributions of Sulfated and Sialylated Oligosaccharides on Bovine, Ovine, and Human Pituitary Glycoprotein Hormones. J Biol Chem (1988) 263:36–44. doi: 10.1016/S0021-9258(19)57352-5 3121612

[65] Stanley P Cummings RD. Structures Common to Different Glycans. In: Varki A Cummings RD Esko JD Stanley P Hart GH Aebi M Darvill AG Kinoshita T Packer NH Prestegard JH Schnaar RL Seeberger PH, editors. Essentials of Glycobiology. Cold Spring Harbor, N.Y.: Cold Spring Harbor Laboratory Press (2017). p. 161–78.

[66] Emmanouel DS Stavropoulos T Katz AI. Role of the Kidney in Metabolism of Gonadotropins in Rats. Am J Physiol (1984) 247:E786–92. doi: 10.1152/ajpendo.1984.247.6.E786 6439050

[67] Illiano A Pinto G Melchiorre C Carpentieri A Faraco V Amoresano A. Protein Glycosylation Investigated by Mass Spectrometry: An Overview. Cells (2020) 9:1986. doi: 10.3390/cells9091986 PMC7564411 32872358

[68] Irungu JA Dalpathado DS Go EP Jiang H Ha H-V Bousfield GR. A Method for Characterizing Sulfated Glycoproteins in a Glycosylation Site-Specific Fashion, Using Ion-Pairing and Tandem Mass Spectrometry. Anal Chem (2006) 78:1181–90. doi: 10.1021/ac051554t 16478110

[69] Weisshaar G Hiyama J Renwick AGC. Site-Specific N-Glycosylation of Human Chorionic Gonadotropin—Structural Analysis of Glycopeptides by One- and Two-Dimensional 1H NMR Spectroscopy. Glycobiology (1991) 1:393–404. doi: 10.1093/glycob/1.4.393 1820200

[70] Weisshaar G Hiyama J Renwick AGC Nimtz M. NMR Investigations of the N–linked Oligosaccharides at Individual Glycosylation Sites of Human Lutropin. Eur J Biochem (1991) 195:257–68. doi: 10.1111/j.1432-1033.1991.tb15702.x 1991473

[71] Hiyama J Weisshaar G Renwick AGC. The Asparagine-Linked Oligosaccharides at Individual Glycosylation Sites in Human Thyrotropin. Glycobiology (1992) 2:401–9. doi: 10.1093/glycob/2.5.401 1457969

[72] Maack T Park CH Camargo MJF. Renal Filtration, Transport, and Metabolism of Proteins. In: Seldin DW Giebisch G, editors. The Kidney: Physiology and Pathophysiology. New York: Raven Press, Ltd. (1992). p. 3005–38.

[73] Park EI Mazella SM Baenziger JU. Rapid Clearance of Sialylated Glycoproteins by the Asialoglycoprotein Receptor. J Biol Chem (2003) 278:4597–602. doi: 10.1074/jbc.M210612200 12464602

[74] Cummings RD McEver RP. C-Type Lectins. In: Varki A Cummings RD Esko JD Stanley P Hart GH Aebi M Darvill AG Kinoshita T Packer NH Prestgard JH Schnaar RL Seeberger PH, editors. Essentials of Glycobiology, vol. pp . Cold Spring Harbor, NY: Cold Spring Harbor Laboratory Press (2017). p. 435–52.

[75] Bousfield GR Butnev VY Butnev VY Nguyen VT Gray CM Dias JA. Differential Effects of a Asparagine^56^ Oligosaccharide Structure on Equine Lutropin and Follitropin Hybrid Conformation and Receptor-Binding Activity. Biochemistry (2004) 43:10817–33. doi: 10.1021/bi049857p 15311943

[76] Jiang X Dias JA He X. Structural Biology of Glycoprotein Hormones and Their Receptors: Insights to Signaling. Mol Cell Endocrinol (2013) 108:7172–6. doi: 10.1016/j.mce.2013.08.021 24001578

[77] Sairam MR Manjunath P. Studies on Pituitary Follitropin. XI. Induction of Hormonal Antagonistic Activity by Chemical Deglycosylation. Mol Cell Endocrinol (1982) 28:139–50. doi: 10.1016/0303-7207(82)90027-2 6290293

