# Supplementary material for: Human FSH Glycoform α-Subunit Asparagine52 Glycans: Major Glycan Structural Consistency, Minor Glycan Variation in Abundance
Source: Front Endocrinol (Lausanne). 2022 Oct 18;13:767661. doi: 10.3389/fendo.2022.767661 (PMC9623679; doi:10.3389/fendo.2022.767661)
Supplement: Supplementary file 1 [file DataSheet_2.pdf]

Supplement to: Human FSH Glycoform α-Subunit Asparagine^{52} Glycans: Major Glycan Structural Consistency, Minor Glycan Variation in Abundance

Viktor Y. Butnev[1], Jeffrey V. May[1], Alan R. Brown[1], Tarak Sharma[1], Vladimir Y. Butnev, William K. White[1], David J. Harvey[2], and George R. Bousfield[1]

[1]Department of Biological Sciences, Wichita State University, Wichita, KS and [2]Department of Biochemistry, University of Oxford, Oxford, UK

Human pituitary FSH microheterogeneity was evaluated at three of four N-glycosylation sites by various mass spectrometry procedures as described in the text. Summary tables and figures are also found in the text. Mass spectra and glycan ion tables associated with singly, doubly, and triply charged glycan spectra in the paper are included in this supplement. The initial study evaluated glycan populations at individual N-glycosylation sites in a highly purified hFSH preparation. Analysis of hFSHα Asn^{52} oligosaccharides was undertaken to determine whether the hypo-glycosylated glycoforms possessed glycans small enough to permit more than one FSH molecule to bind the putative FSHR trimers [1; 2; 3]. Collision induced fragmentation indicated αAsn^{52} tetra-antennary glycan masses were associated with tri-antennary glycans possessing lactosamine repeats. Sialic acid linkage was evaluated for αAsn^{52} glycans, as well.

Methods

Oligosaccharides were obtained from individual N-glycosylation sites on FSH α and β subunit preparations and analyzed by mass spectrometry as described in the accompanying paper. Individual mass spectra can be found in this document, while associated data tables are for provided in 3 Excel files. Each file represents a set of experiments: FSH subunit and site-specific glycans, FSH glycoform fraction αAsn^{52} glycans,

and another FSHα Asn^{52} glycan sample. Three supplement summary tables in this document use the glycan identities established in Table 1 to compile various glycan ions, glycan composition, and relative abundance for each glycosylation site or glycoform preparation.

Results

ESI-MS spectra in Fig. S1 reveal glycan populations from each subunit resemble each other, while differing from those derived from the complementary subunit. Several glycan ions encountered are identified by structure diagram numbers used in summary Tables 1 and S13.

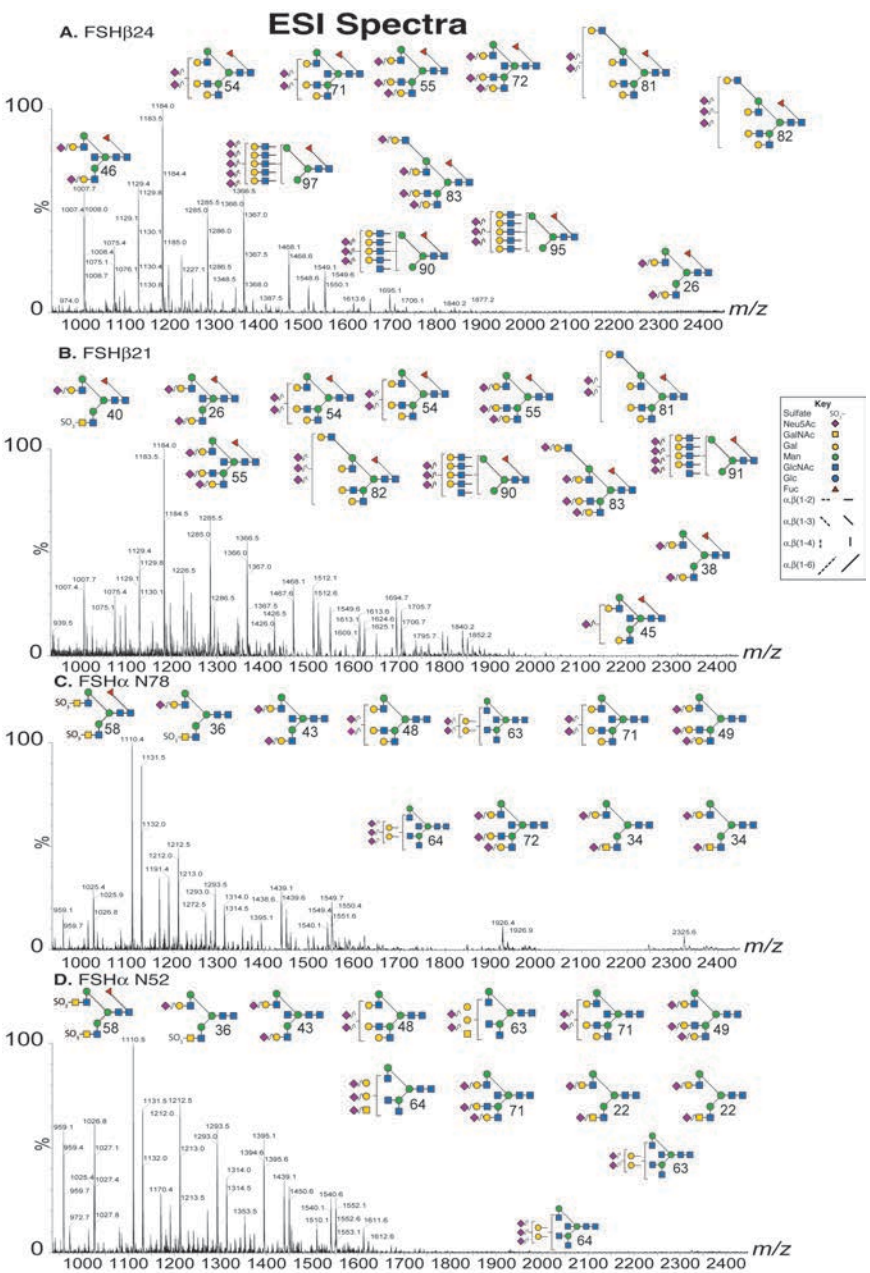

Figure S1. ESI-MS spectra for FSH subunit glycans. A. 24kDa-FSHβ glycans. B. 21kDa-FSHβ Asn[7] glycans. C. FSHα Asn[78] glycans. D. FSHα Asn[52] glycans. Glycan structures displayed using the CFG/Oxford system, which identifies monosaccharides with colored symbols and linkages without the use of labels (see key).

Singly charged FSH glycan ions are shown below in Fig. S2. Glycan ions are listed in Excel Tables S1-S4 (each table is a separate tab). Observed and calculated *m/z* values are listed along with monosaccharide composition. The Roman numerals indicate neutral oligosaccharide structures, which are used to organize glycan data in summary Table S13. Doubly charged glycan ions from the spectra in Fig. 4 are listed in Excel Tables S5-S8. Triply charged FSH glycan ions from Fig. 5 are listed in Excel Tables S9-S12. Fragmented ions are indicated by the use of bold text in these tables.

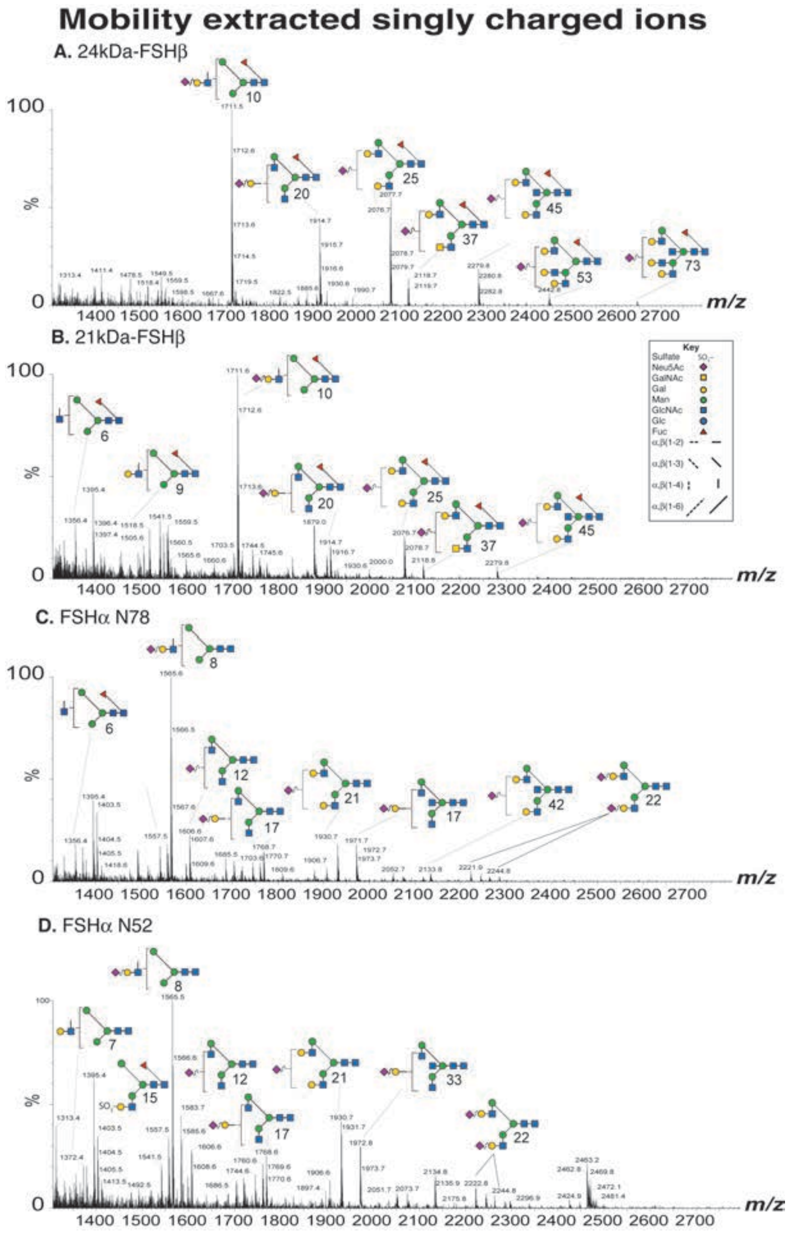

Figure S2. Ion mobility mass spectra for single-charge FSH glycans. A. Glycans derived from 24kDa-FSHβ, Asn[7] and Asn[24]. B. Glycans derived from 21kDa-hFSHβ, Asn[7] only. C. Glycans derived from hFSHα Asn[78]. D. Glycans derived from hFSHα Asn[52]. Glycan structures displayed using the CFG/Oxford system (see key). Glycan ions are tabulated in supplement Excel Tables S1, S2, S3, and S4, respectively.

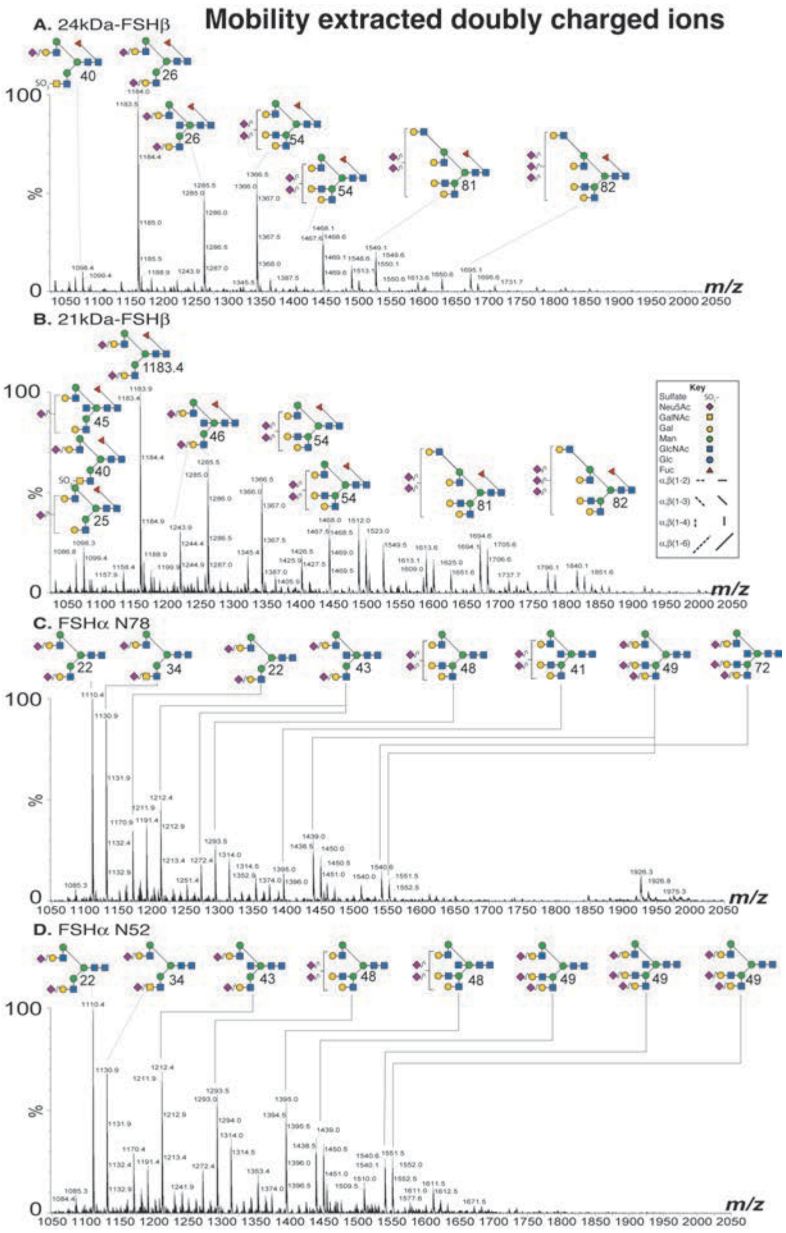

Figure S3. Ion mobility mass spectra for double charged FSH glycans. A. Glycans derived from 24kDa-FSHβ, both Asn[7] and Asn[24]. B. Glycans derived from 21kDa-FSHβ, Asn[7] only. C. Glycans derived from hFSHα Asn[78]. D. Glycans derived from hFSHα Asn[52]. Glycan ions are tabulated in supplement Excel Tables S5, S6, S7, and S8, respectively.

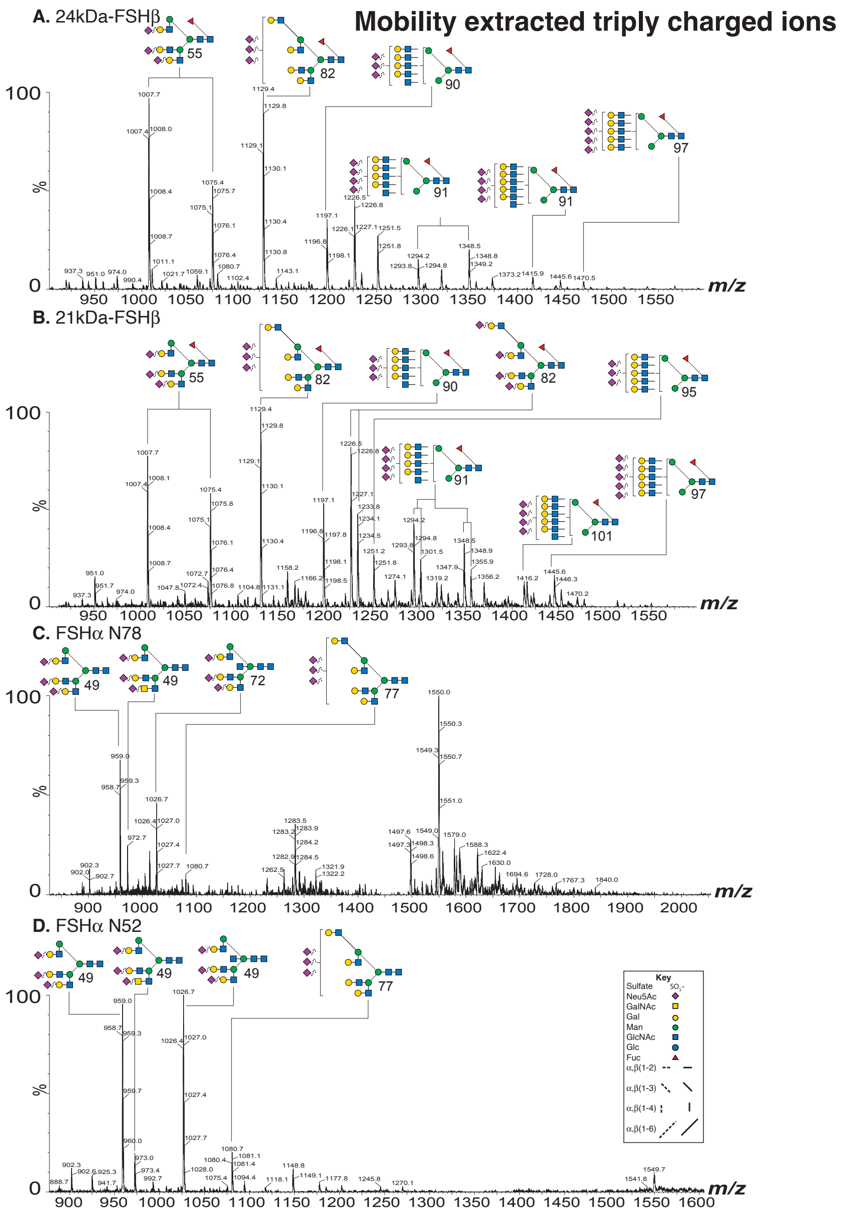

Figure S4. Ion mobility mass spectra for triply charged FSH glycans. A. Glycans derived from 24kDa-FSHβ, Asn^{7} and Asn^{24}. B. Glycans derived from 21kDa-FSHβ, Asn^{7} only. C. Glycans derived from hFSHα Asn^{52}. D. Glycans derived from hFSHα Asn^{78}. Glycan ions are tabulated in supplement Excel Tables S9, S10, S11, and S12, respectively.

Glycans from both FSHβ N-glycosylation sites in 24kDa-FSHβ, the Asn[7] glycosylation site in 21kDa-FSHβ, Asn[78] and Asn[52] glycosylation sites in FSHα are summarized in Table S13. The numbers in column 1 identify glycans according to the system employed in Table 1. Roman numerals identify neutral glycan structures to keep tables compact.

Table S13. Composition and abundance of hFSH glycans (all ions, all glycosylation sites).

Glycan	*m/z*	Ion[a]	z	Glycan mass	Composition					Quantitation (% total)				Neutral glycan	
					Hex	HexNAc	Fuc	Neu5Ac	SO_3	β24	β21	αN78	αN52	Number	Mass
2	1331.4	a	1	1234.4	5	2	0	0	0	-	-	0.07	-	**I**	1234.4
6	1356.4	a	1	1259.5	3	3	1	0	0	-	0.09	0.12	0.05	**II**	1259.5
7	1372.4	a	1	1275.5	4	3	0	0	0	-	-	0.09	-	**III**	1275.5
8	1565.6	b	1	1566.5				1	0	-	-	0.61	0.25		
9	1518.4	a	1	1421.5			1	0	0	-	0.07	0.03	-	**VI**	1421.5
10	1711.6	b	1	1712.6				1	0	0.14	-	-	-		
12	1606.6	b	1	1607.6	3	4	0	1	0	-	-	0.15	0.08	**IV**	1316.5
13	1395.4	b	1	1396.4				0	1	0.01	0.06	0.22	0.14	**V**	
14	1559.5	a	1	1462.5			1	0	0	0.01	-	0.03	-	**VII**	1462.5
15	1541.5	b	1	1542.5				0	1	0.01	0.05	0.10	0.06	**VIII**	
17	1768.6	b	1	1769.6	4	4	0	1	0	-	-	0.11	0.07	**IX**	1478.5
18	1557.5	b	1	1558.5				0	1	-	0.06	0.10	0.10	**X**	
19	1721.6	a	1	1624.6			1	0	0	-	-	0.02	0.02	**XII**	1624.6
20	1914.7	b	1	1915.6				1	0	0.24	0.38	-	-		
	1005.8	e	2												
21	1930.7	b	1	1931.7	5	4	0	1	0	-	-	0.12	0.13	**XIII**	1640.6
22	2221.8	b	1	2222.8				2	0	0.45	0.58	20.28	13.63		
	2243.8	b(Na)	2												
	1110.4	c	2												
	1170.4	e	2												
23	1719.5	b	1	1720.5				0	1	-	-	0.04	0.04	**XIV**	
25	2076.7	b	1	2077.7			1	1	0	2.03	1.84	0.24	0.22	**XIX**	1786.7
	1055.9	f	2												
	1086.9	e	2												
26	2367.8	b	1	2368.8				2	0	13.36	11.17	1.02	0.96		
	1183.4	c	2												
	1232.4	f	2												
	1243.4	e	2												
27	1077.8	c	2	2157.7				1	1	0.67	0.43	-	-	**?**	
29	1809.6	b	1	1810.7	3	5	0	1	0	-	-	0.02	0.01	**XI**	1519.6
30	1598.5	b	1	1599.5				0	1	-	-	0.06	0.04		
31	1744.5	b	1	1745.6			1	0	1	-	-	0.06	0.04	**XV**	1665.6

33	1971.7	b	1	1972.7	4	5	0	1	0	-	-	0.12	0.09	XVI	1681.6
34	2262.8	b	1	2263.8				2	0	0.18	0.38	19.75	9.89	XVI, XVII	
	1130.9	c	2												
	1179.9	e	2												
	1190.9	e(Na)	2												
35	1760.6	b	1	1923.6				0	1	-	-	0.06	0.06	XVII	
36	1025.3	c	2	2052.7				1	1	0.12	0.92	3.69	3.06		
37	2117.8	b	1	2118.8			1	1	0	0.55	0.76	0.26	0.10	XX, XXI	1827.7
	1076.4	f	2												
	1107.4	e	2												
38	1203.9	c	2	2409.9				2	0	0.85	0.73	-	-	XXI	
39	1906.6	b	1	1907.6				0	1	0.01	0.01	0.03	0.02		
40	1098.4	c	2	2198.7				1	1	1.29	1.84	0.17	0.23		
42	2133.8	b	1	2134.8	5	5	0	1	0	-	-	0.03	0.05	XXII	1843.7
43	1211.9	c	2	2425.8				2	0	0.34	0.63	10.14	9.63		
	1271.9	e(Na)	2												
45	2279.8	b	1	2280.8			1	1	0	2.03	3.30	-	-	XXIV	1989.7
	1188.4	e													
	1199.4	e(Na)													
	1157.4	c													
46	1285.0	c	2	2571.9				2	0	6.95	6.76	-	-		
	1344.9	e	2												
48	1293.0	c	2	2587.9	6	5	0	2	0	0.45	0.52	6.24	8.37	XXV, XXVI	2005.7
	1352.9	e(Na)	2												
49	1438.5	c	2	2879.0				3	0	0.36	0.17	10.52	14.40		
	1449.5	e(Na)	2												
	958.7	d	3												
53	2441.9	b	1	2442.9			1	1	0	1.10	1.08	0.20	0.39	XXXI, XXXII	2151.8
	1238.4	f	2												
	1269.4	e	2												
54	1366.0	c	2	2734.0				2	0	7.93	6.29	0.51	0.45		
	1425.9	e	2												
55	1511.5	c	2	3025.0				3	0	12.02	8.49	-	-		
	1522.5	c(Na)	2												
	1007.3	d	3												
56	940.3	c	2	1882.6	3	6	0	0	2	0.12	0.73	0.93	0.71	XVIII	1722.6
58	1013.3	c	2	2028.6			1	0	2	0.21	1.06	2.25	1.21	XXIII	1868.7
59	1118.9	c	2	2239.8				1	1	0.07	0.09	-	-		
65	1207.9	c	2	2417.8	5	6	0	1	1	-	-	1.05	0.92	XXVII-XXX	2046.8
63	1313.5	c	2	2628.9				2	0	-	-	4.61	5.00		
	1373.4	e	2												
64	1459.0	c	2	2920.0				3	0	0.05	0.08	3.48	2.84		
	1470.0	c(Na)	2												

	972.3	d	3												
68	1386.5	c	2	2775.0			1	2	0	0.88	0.71	0.45	0.15	**XXXIII, XXXIV**	2192.8
69	1280.9	c	2	2563.9				1	1	0.39	0.81	-	-		
71	1394.5	c	2	2791.0	6	6	0	2	0	0.27	0.35	2.97	7.25	**XXXV, XXXVI**	2208.8
	1454.5	e(Na)	2												
72	1540.0	c	2	3082.1				3	0	0.32	0.16	5.61	12.55		
	1551.0	c(Na)	2												
	1026.4	d	3												
73	1340.0	c	2	2645.9			1	1	0	0.38	0.55	0.23	-	**XXXVII, XXXVIII**	2354.9
74	1467.5	c	2	2937.0				2	0	4.06	3.77	0.35	0.67		
	1527.5	e	2												
75	1613.1	c	2	3228.1				3	0	5.56	5.72	-	0.20		
	1624.1	c(Na)	2												
	1075.1	d	3												
76	1475.6	c	2	2953.0	7	6	0	2	0	0.19	0.16	0.36	0.74	**XXXIX, XL**	2370.9
77	1621.1	c	2	3244.1				3	0	0.87	0.28	1.32	2.81		
	1632.1	c(Na)	2												
	1080.4	d	3												
78	1177.4	d	3	3535.2				4	0	0.38	0.28	0.26	0.53		
	1184.7	d(Na)	3												
80	1452.0	c	2	2808.0			1	1	0	0.20	0.23	-	-	**XLII, XLIII**	2516.9
81	1548.6	c	2	3099.1				2	0	2.85	2.79	-	-		
	1608.5	e	2												
82	1694.1	c	2	3390.2				3	0	10.60	8.92	-	-		
	1705.1	c(Na)	2												
	1129.1	d	3												
83	1839.6	c	2	3681.3				4	0	5.08	8.21	-	-		
	1226.1	d	3												
	1850.6	c(Na)	2												
	1233.4	d	3												
	1861.6	c(Na)	2												
84	1641.6	c	2	3285.1	6	7	0	3	0	-	-	0.42	0.52	**XLI**	2411.9
	1094.1	d	3												
85	1569.1	c	2	3140.1			1	2	0	0.31	0.27	-	-	**XLIV**	2557.9
87	1722.6	c	2	3447.2	7	7	0	3	0	0.21	0.17	0.31	1.13	**XLV**	2573.9
	1148.1	d	3												
89	1650.1	c	2	3302.2			1	2	0	0.97	0.99	-	-	**XLVI**	2720.0
90	1795.6	c	2	3593.3				3	0	3.59	3.97	-	-		
	1196.8	d	3												
	1806.6	c(Na)	2												
91	1941.2	c	2	3884.3				4	0	1.85	3.88	-	-		
	1293.8	d	3												
	1952.2	c(Na)	2												

	1301.1	d	3												
	1963.2	c(Na_2)	2												
94	1731.1	c	2	3464.2	8	7	1	2	0	0.42	0.29	-	-	**XLVII**	2882.0
95	1876.7	c	2	3755.3				3	0	2.78	1.91	-	-		
	1250.8	d	3												
	1887.7	c(Na)	2												
96	2022.2	c	2	4046.4				4	0	2.23	2.79	-	-		
	1347.8	d	3												
	2033.2	c(Na)	2												
	1355.1	d	3												
97	5.8	d	3	4337.5				5	0	0.40	0.77	-	-		
98	1269.8	d	3	3812.3	8	8	0	3	0	0.08	0.13	0.20	0.18	**XLVIII**	2939.1
100	1832.7	c	2	3667.3			1	2	0	0.16	0.14	-	-	**XLIX**	3085.1
101	1978.2	c	2	3958.4				3	0	1.00	0.82	-	-		
	1318.5	d	3												
	1989.2	c(Na)	2												
102	1415.5	d	3	4249.5				4	0	0.74	1.00	-	-		
	1422.8	d	3												
103	1512.5	d	3	4540.6				5	0	0.10	0.15	-	-		
106	1372.5	d	3	4120.4	9	8	1	3	0	0.54	0.24	-	-	**L**	3247.2
107	1469.6	d	3	4411.5				4	0	0.50	0.51	-	-		
	1476.9	d(Na)	3												
108	1566.5	d	3	4702.6				5	0	0.12	0.06	-	-		
110	1440.2	d	3	4323.5	9	9	1	3	0	0.20	0.31	-	-	**LI**	3450.3
111	1537.2	d	3	4614.6				4	0	0.12	0.06	-	-		
113	1494.2	d	3	4485.6	10	9	1	3	0	0.10	-	-	-	**LII**	3612.3

[a]Ions: a = $[M + HPO_4]^-$, b = $[M – H]^-$, c = $[M – 2H]^{2-}$ d = $[M – 3H]^{3-}$, e = $[M – H + HPO_4]^{2-}$, f = $[M – H + Cl]^2$

Table S14. Glycan structures

(Structures shown as CFG/OGI hybrids and listed in order of neutral glycan mass, neutral glycan structures indicated by Roman numerals (Table S13). Actual glycans detected indicated by Arabic numerals from Table 1. Composition: H = hexose, N = N-acetylhexosamine, F = fucose, N5A = Neu5Ac, S = SO_4, glycan mass shown for each structure). Related charged structures shown above each neutral glycan. Orange bars separate the table into groups of structurally related glycans.

			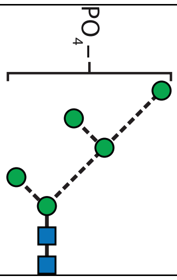		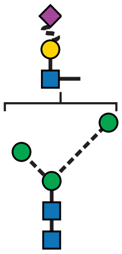
			**I, 83**		**III, 4**
			$H_5N_2P_1$		$H_4N_3N5A_1$
			1314.4		1566.5
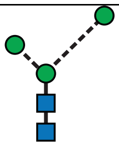	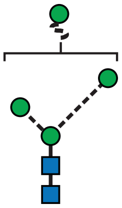	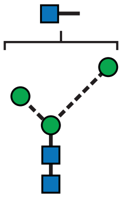	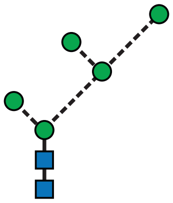	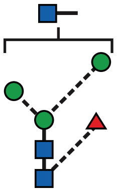	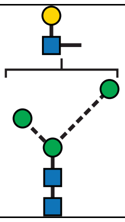
**LIII, 82**	**LXX, 114**	**LIV, 83**	**I, 1**	**II, 2**	**III, 3**
H_3N_2	H_4N_2	H_3N_3	H_5N_2	$H_3N_3F_1$	H_4N_3
910.3	1095.4	1113.3	1234.4	1259.5	1275.5
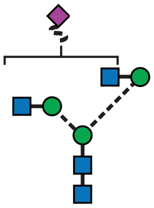	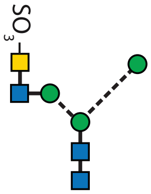	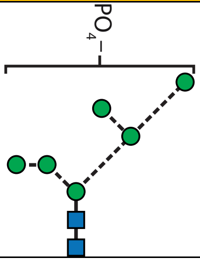	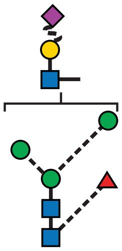		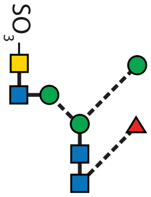
**IV, 7**	**V, 8**	**LV, 84**	**VI, 6**		**VIII, 10**
$H_3N_4N5A_1$	$H_3N_4S_1$	$H_6N_2P_1$	H_4N_3 F_1N5A_1		$H_3N_4F_1S_1$
1607.6	1396.4	1476.5	1712.6		1542.5
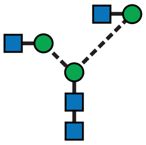	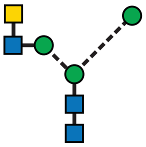	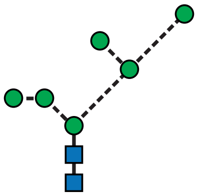	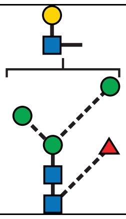	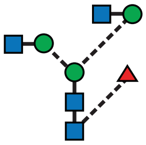	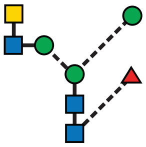
**IV, 86**	**V**	**LV**	**VI, 5**	**VII, 9**	**VIII**
H_3N_4	H_3N_4	H_6N_2	$H_4N_3F_1$	$H_3N_4F_1$	$H_3N_4F_1$
1316.5	1316.5	1396.5	1421.5	1462.5	1462.5

				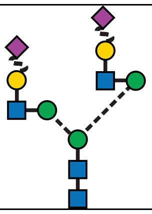	
				**XIII, 16**	
				$H_5N_4N5A_2$	
				2222.8	
		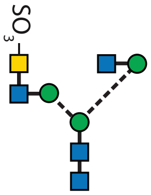		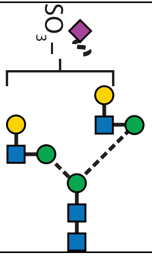	
		**XI, 22**		**XIII, 88**	
		$H_3N_5S_1$		$H_5N_4N5A_1S_1$	
		1599.5		2011.6	
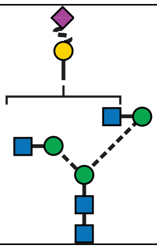	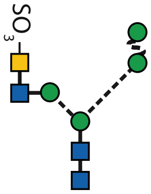	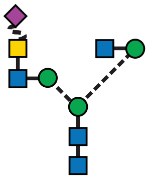	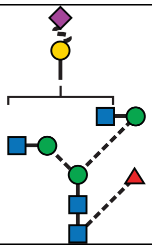	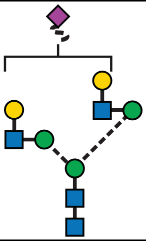	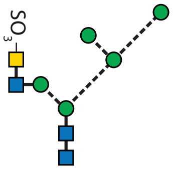
**IX, 11**	**X, 12**	**XI, 21**	**XII, 14**	**XIII, 15**	**XIV, 17**
$H_4N_4N5A_1$	$H_4N_4S_1$	$H_3N_5N5A_1$	$H_4N_4F_1N5A_1$	$H_5N_4N5A_1$	$H_5N_4S_1$
1769.6	1558.5	1810.7	1915.7	1931.7	1720.5
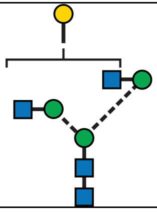	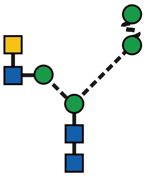	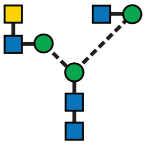	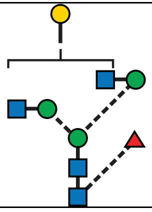	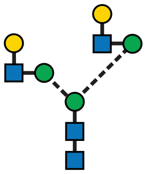	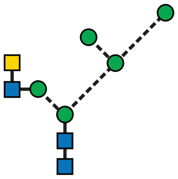
**IX**	**X, 87**	**XI, 89**	**XII, 13**	**XIII**	**XIV**
H_4N_4	H_4N_4	H_3N_5	$H_4N_4F_1$	H_5N_4	H_5N_4
1478.5	1478.5	1519.6	1624.6	1640.6	1640.6

		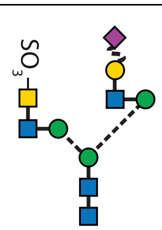		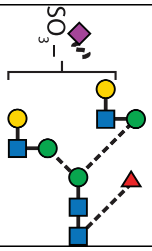	
		**XVII, 27**		**XIX, 20**	
		$H_4N_5N5A_1S_1$		$H_5N_4F_1N5A_1S_1$	
		2052.7		2157.7	
	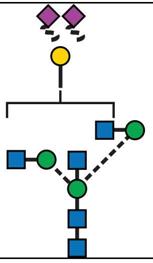	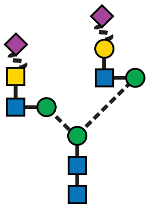	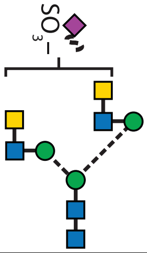	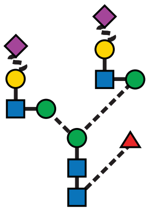	
	**XVI, 25**	**XVII, 25**	**XVIII, 97**	**XIX, 19**	
	$H_4N_5N5A_2$	$H_4N_5N5A_2$	$H_3N_6N5A_1S_1$	$H_5N_4F_1N5A_2$	
	2263.8	2263.8	2093.7	2368.8	
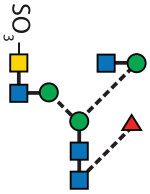	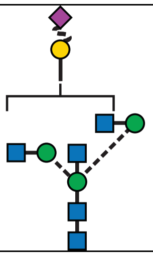	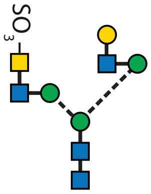	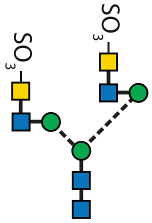	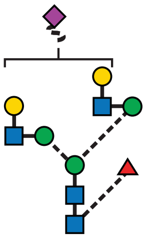	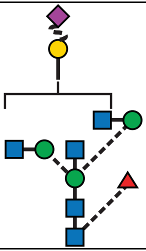
**XV, 23**	**XVI, 24**	**XVII, 26**	**XVIII, 41**	**XIX, 18**	**XX, 28**
$H_3N_5F_1S_1$	$H_4N_5N5A_1$	$H_4N_5S_1$	$H_3N_6S_2$	$H_5N_4F_1N5A_1$	$H_4N_5F_1N5A_1$
1745.6	1972.4	1923.6	1882.6	2077.7	2118.8
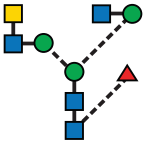	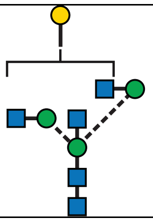	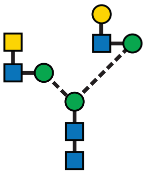	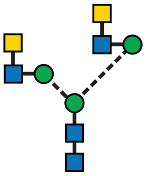	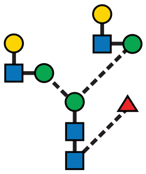	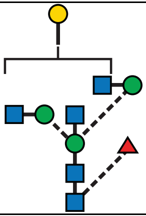
**XV**	**XVI, 90**	**XVII, 90**	**XVIII**	**XIX**	**XX**
$H_3N_5F_1$	H_4N_5	H_4N_5	H_3N_6	$H_5N_4F_1$	$H_4N_5F_1$
1665.6	1681.6	1681.6	1722.6	1786.7	1827.7

		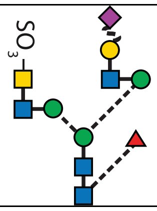			
		**XXI, 31**			
		$H_4N_5F_1N5a_1S_1$			
		2198.7			
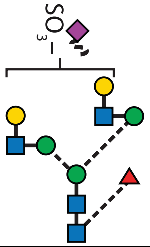		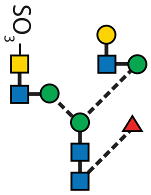	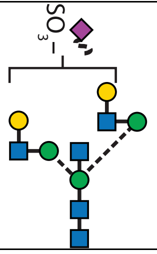		
**XIX, 20**		**XXI, 30**	**XXII, 92**		
$H_5N_4F_1N5A_1S_1$		$H_4N_5F_1S_1$	$H_5N_5N5AS_1$		
2157.7		1907.6	2214.7		
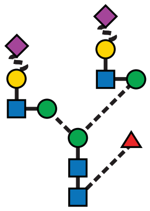		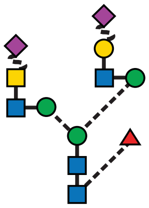	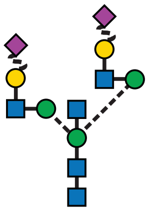	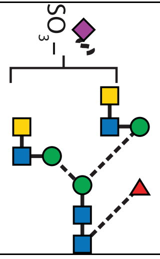	
**XIX, 19**		**XXI, 29**	**XXII, 33**	**XXIII, 43**	
$H_5N_4F_1N5A_2$		$H_4N_5F_1N5A_2$	$H_5N_5N5A_2$	$H_3N_6F_1N5A_1S_1$	
2368.8		2409.9	2425.8	2239.8	
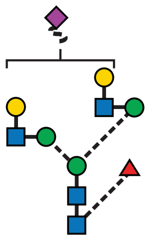	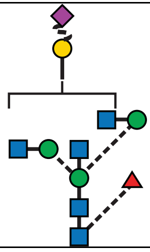	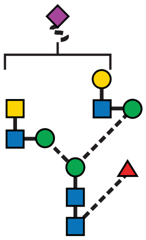	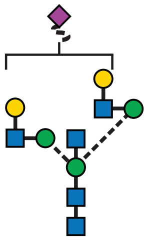	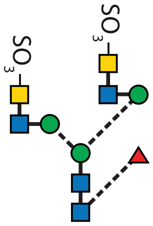	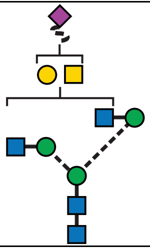
**XIX, 18**	**XX, 28**	**XXI, 28**	**XXII, 32**	**XXIII, 42**	**LVI, 85**
$H_5N_4F_1N5A_1$	$H_4N_5F_1N5A_1$	$H_4N_5F_1N5A_1$	$H_5N_5N5A_1$	$H_3N_6F_1S_2$	H_4N_6 $N5A_1$
2077.7	2118.8	2118.8	2134.8	2028.6	2175.8
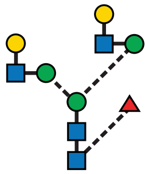	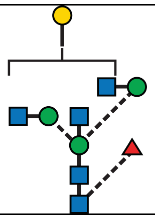	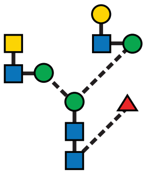	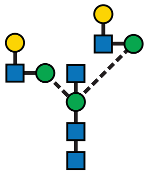	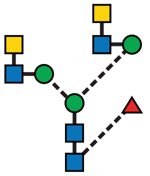	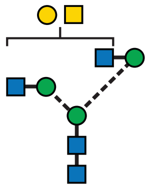
**XIX**	**XX**	**XXI**	**XXII, 91**	**XXIII**	**LVI**
$H_5N_4F_1$	$H_4N_5F_1$	$H_4N_5F_1$	H_5N_5	$H_3N_6F_1$	H_4N_6
1786.7	1827.7	1827.7	1843.7	1868.7	1884.7

		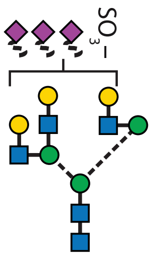	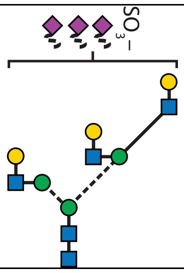		
		**XXV, 96**	**XXVI, 96**		
		$H_6N_5N5A_3S_1$	$H_6N_5N5A_3S_1$		
		2959.0	2959.0		
		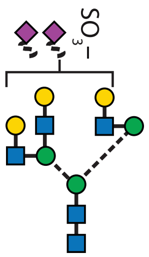	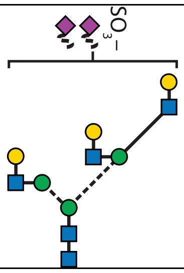		
		**XXV, 95**	**XXVI, 95**		
		$H_6N_5N5A_2S_1$	$H_6N_5N5A_2S_1$		
		2667.9	2667.9		
		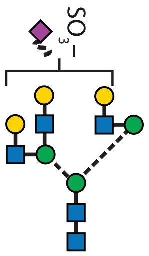	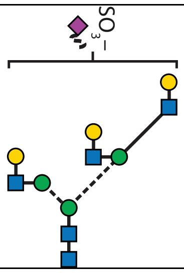		
		**XXV, 94**	**XXVI, 94**		
		$H_6N_5N5A_1S_1$	$H_6N_5N5A_1S_1$		
		2376.8	2376.8		
		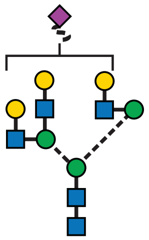	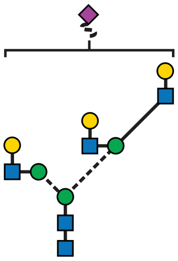	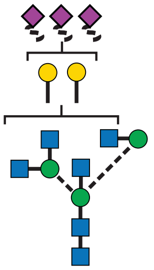	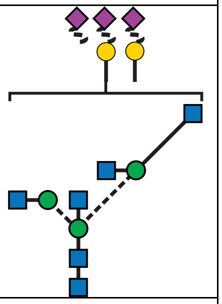
		**XXV, 93**	**XXVI, 93**	**XXVII, 46**	**XXVII, 46**
		$H_6N_5N5A_1$	$H_6N_5N5A_1$	$H_5N_6N5A_3$	$H_5N_6N5A_3$
		2296.8	2296.8	2920.0	2920.0

	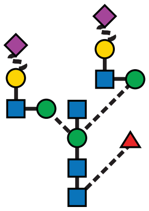	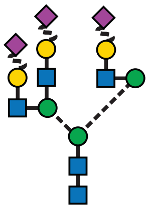	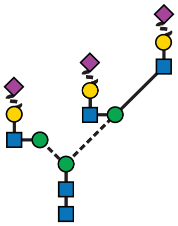	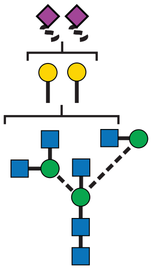	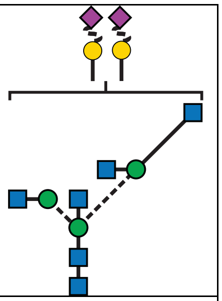
	**XXIV, 35**	**XXV, 37**	**XXVI, 37**	**XXVII, 45**	**XXVII, 45**
	$H_5N_5F_1N5A_2$	$H_6N_5N5A_3$	$H_6N_5N5A_3$	$H_5N_6N5A_2$	$H_5N_6N5A_2$
	2571.9	2879.0	2879.0	2628.9	2628.9
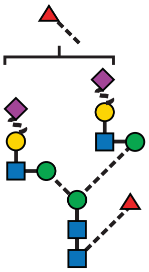	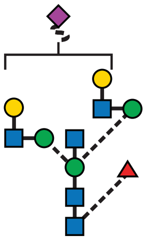	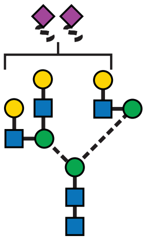	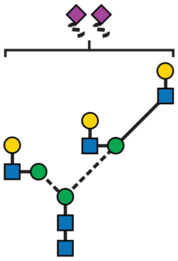	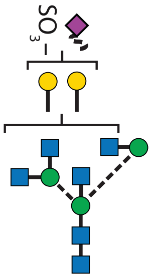	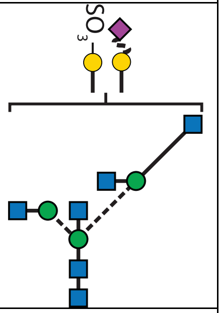
**nnn**	**XXIV, 34**	**XXV, 36**	**XXVI, 36**	**XXVII, 44**	**XXVII, 44**
$H_5N_5F_1N5A_2$	$H_5N_5F_1N5A_1$	$H_6N_5N5A_2$	$H_6N_5N5A_2$	$H_5N_6N5A_1S_1$	$H_5N_6N5A_1S_1$
*m/z*1256.5	2280.8	2587.9	2587.9	2417.8	2417.8
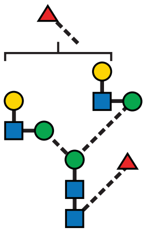	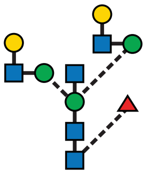	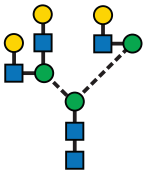	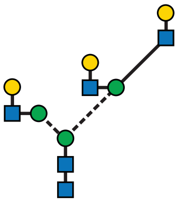	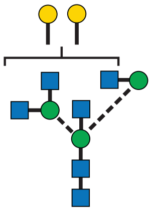	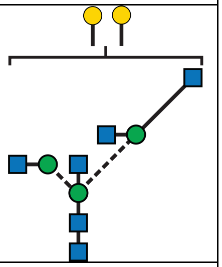
**nnn**	**XXIV**	**XXV**	**XXVI**	**XXVII**	**XXVIII**
$H_5N_4F_2$	$H_5N_5F_1$	H_6N_5	H_6N_5	H_5N_6	H_5N_6
1932.7	1989.7	2005.7	2005.7	2046.8	2046.8
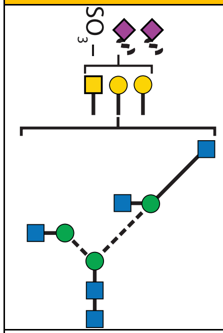	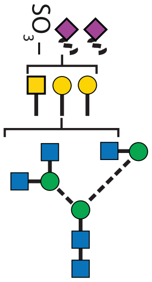				
**XXIX , 100**	**XXX, 100**				
$H_5N_6N5A_2S_1$	$H_5N_6N5A_2S_1$				
2708.9	2708.9				

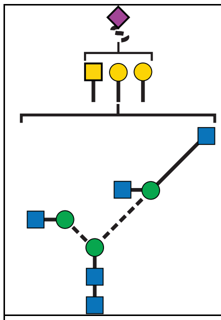	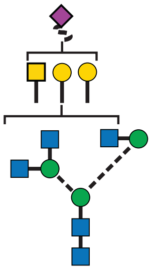				
**XXIX , 99**	**XXX, 99**				
$H_5N_6N5A_1$	$H_5N_6N5A_1$				
2337.8	2337.8				
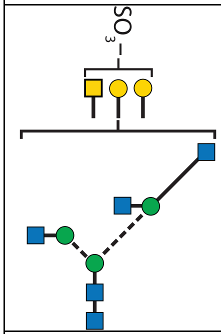	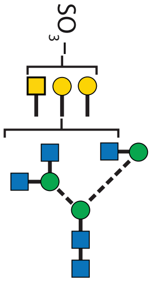				
**XXIX , 98**	**XXX, 98**				
$H_5N_6S_1$	$H_5N_6S_1$				
2126.7	2126.7				
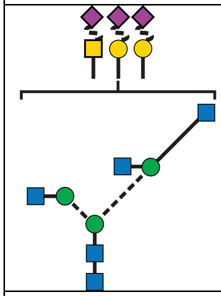	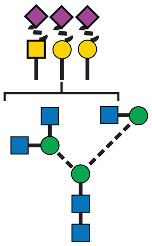	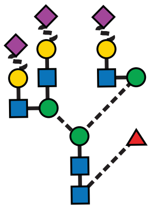	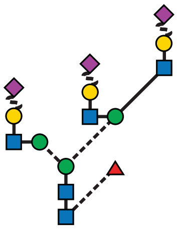		
**XXIX, 46**	**XXX, 46**	**XXXI, 40**	**XXXII, 40**		
$H_5N_6N5A_3$	$H_5N_6N5A_3$	$H_6N_5F_1N5A_3$	$H_6N_5F_1N5A_3$		
2920.0	2920.0	3025.0	3025.0		
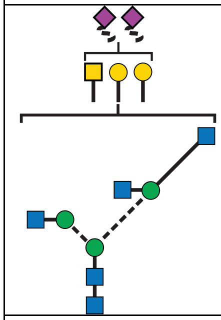	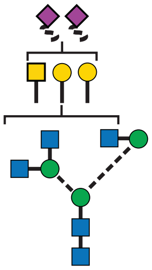	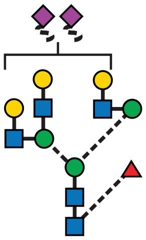	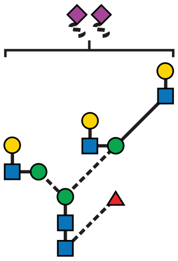		
**XXIX, 45**	**XXX, 45**	**XXXI, 39**	**XXXII, 39**		
$H_5N_6N5A_2$	$H_5N_6N5A_2$	$H_6N_5F_1N5A_2$	$H_6N_5F_1N5A_2$		
2628.9	2628.9	2734.0	2734.0		

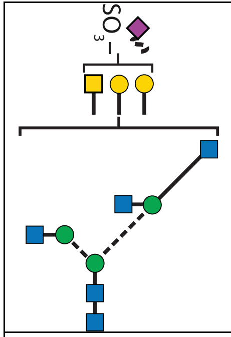	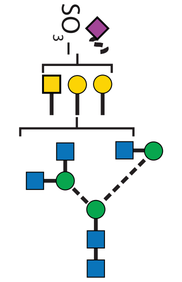	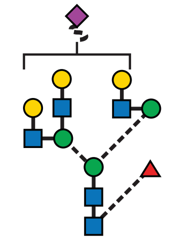	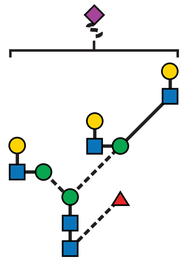	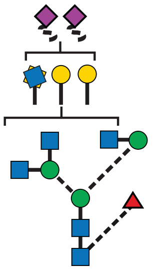	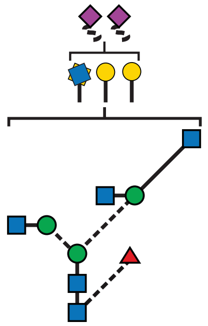
**XXIX, 44**	**XXX, 44**	**XXXI, 38**	**XXXII, 38**	**XXXIII, 47**	**XXXIV, 47**
$H_5N_6N5A_1S_1$	$H_5N_6N5A_1S_1$	$H_6N_5F_1N5A_1$	$H_6N_5F_1N5A_1$	$H_5N_6F_1N5A_2$	$H_5N_6F_1N5A_2$
2417.8	2417.8	2442.9	2442.9	2775.0	2775.0
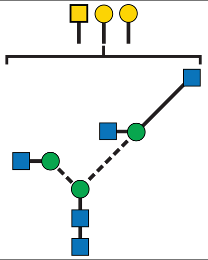	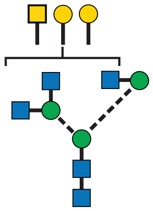	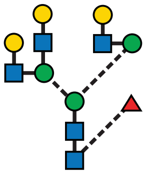	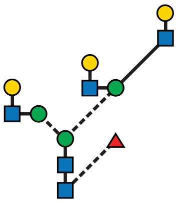	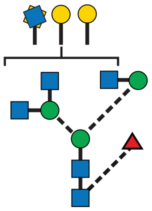	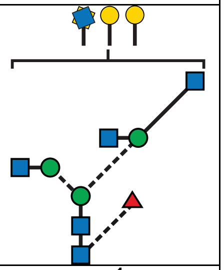
**XXIX**	**XXX**	**XXXI**	**XXXII**	**XXXIII[1], 101**	**XXXIV[1], 101**
H_5N_6	H_5N_6	$H_6N_5F_1$	$H_6N_5F_1$	H_5N_6 F_1	H_5N_6 F_1
2046.8	2046.8	2151.8	2151.8	2192.8	2192.8

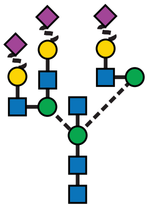				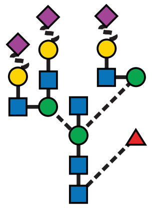	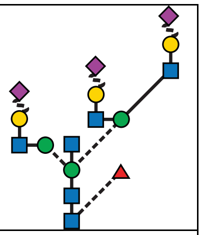
**XXXV, 50**				**XXXVII, 53**	**XXXVIII, 53**
$H_6N_6N5A_3$				$H_6N_6F_1N5A_3$	$H_6N_6F_1N5A_3$
3082.1				3228.1	3228.1
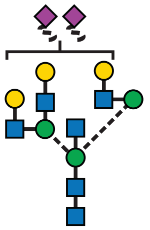				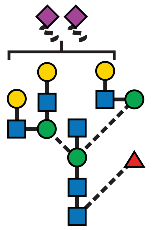	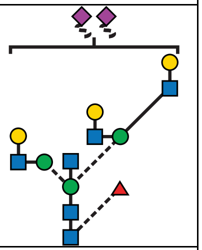
**XXXV, 49**				**XXXVII, 52**	**XXXVIII, 52**
$H_6N_6N5A_2$				$H_6N_6F_1N5A_2$	$H_6N_6F_1N5A_2$
2791.0				2937.0	2937.0
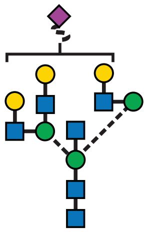		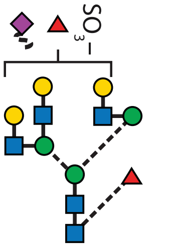	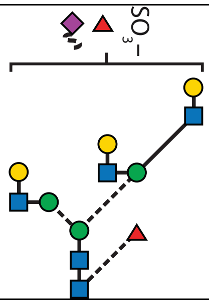	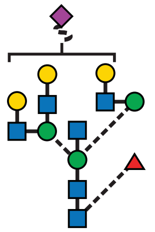	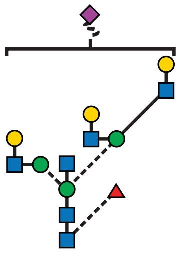
**XXXV, 102**		nnn	nnn+1	**XXXVII, 51**	**XXXVIII, 51**
$H_6N_6N5A_1$		$H_6N_5F_2N5A_1S_1$	$H_6N_5F_2N5A_1S_1$	$H_6N_6F_1N5A_1$	$H_6N_6F_1N5A_1$
2499.9		*m/z*1439.0	*m/z*1439.0	2645.9	2645.9
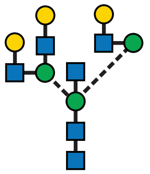	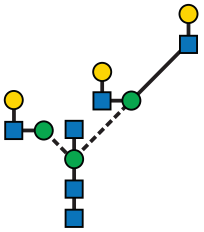	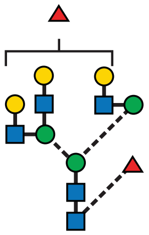	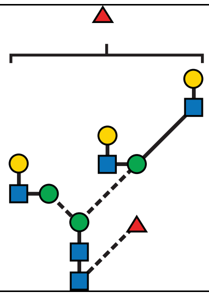	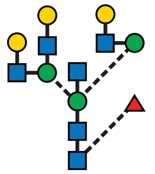	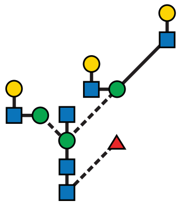
**XXXV**	**XXXVI**	nnn	nnn+I	**XXXVII**	**XXXVIII**
H_6N_6	H_6N_6	$H_6N_5F_2$	$H_6N_5F_2$	$H_6N_6F_1$	$H_6N_6F_1$
2208.8	2208.8	2297.8	2297.8	2354.9	2354.9

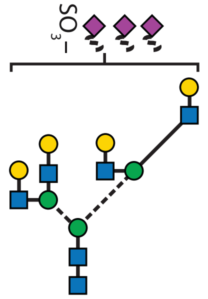	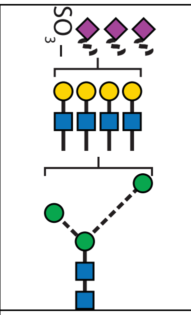		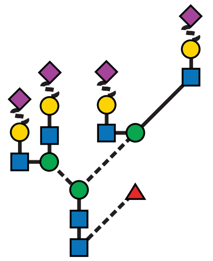	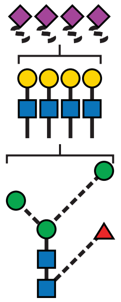	
**XXXIX, 103**	**XL, 103**		**XLII, 60**	**XLIII, 60**	
$H_7N_6N5A_3S_1$	$H_7N_6N5A_3S_1$		$H_7N_6F_1N5A_4$	$H_7N_6F_1N5A_4$	
3324.1	3324.1		3681.3	3681.3	
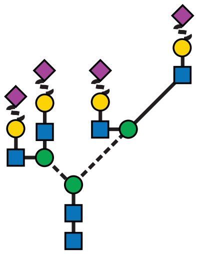	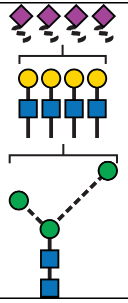		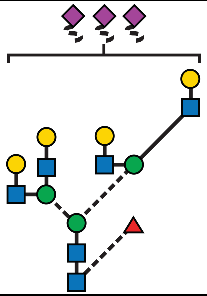	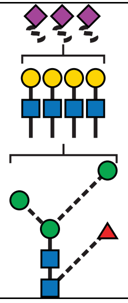	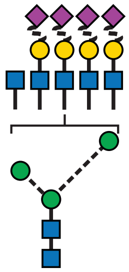
**XXXIX, 56**	**XL, 56**		**XLII, 59**	**XLIII, 59**	**XLV, 106**
$H_7N_6N5A_4$	$H_7N_6N5A_4$		$H_7N_6F_1N5A_3$	$H_7N_6F_1N5A_3$	$H_7N_7N5A_4$
3535.2	3535.2		3390.2	3390.2	3738.3
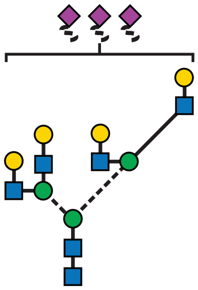	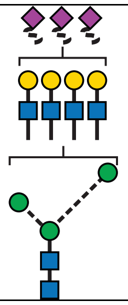		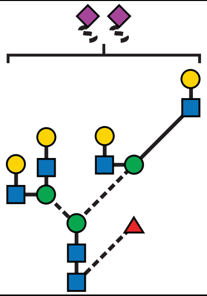	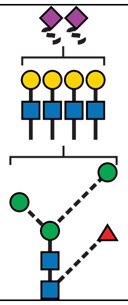	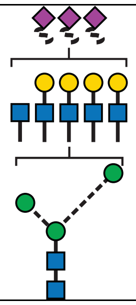
**XXXIX, 55**	**XL, 55**		**XLII, 58**	**XLIII, 58**	**XLV, 63**
$H_7N_6N5A_3$	$H_7N_6N5A_3$		$H_7N_6F_1N5A_2$	$H_7N_6F_1N5A_2$	$H_7N_7N5A_3$
3244.1	3244.1		3099.1	3099.1	3447.2
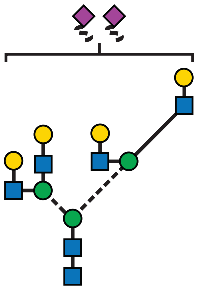	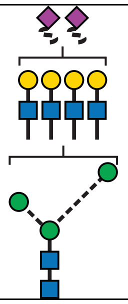	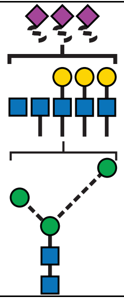	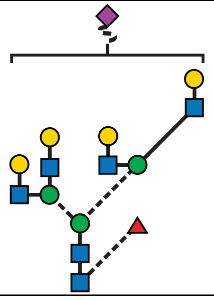	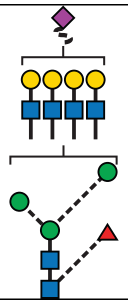	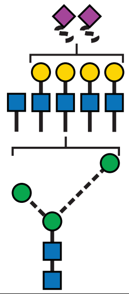
**XXXIX, 54**	**XL, 54**	**XLI, 61**	**XLII, 57**	**XLIII, 57**	**XLV, 105**
$H_7N_6N5A_2$	$H_7N_6N5A_2$	$H_6N_7N5A_3$	$H_7N_6F_1N5A_1$	$H_7N_6F_1N5A_1$	$H_7N_7N5A_2$
2953.0	2953.0	3285.1	2808.0	2808.1	3156.1

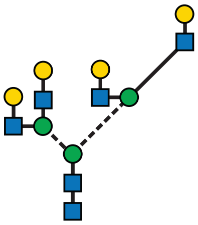	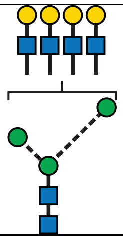	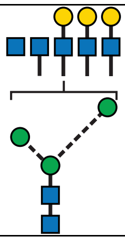	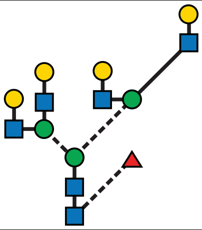	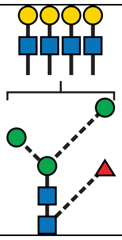	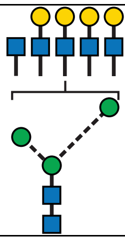
**XXXIX**	**XL**	**XLI**	**XLII**	**XLIII**	**XLV**
H_7N_6	H_7N_6	H_6N_7	$H_7N_6F_1$	$H_7N_6F_1$	H_7N_7
2370.9	2370.9	2411.9	2516.9	2516.9	2573.9

				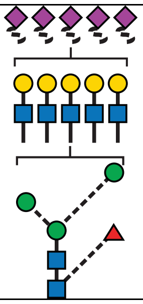	
				**XLVII, 70**	
				$H_8N_7F_1N5A_5$	
				4337.5	
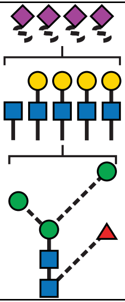				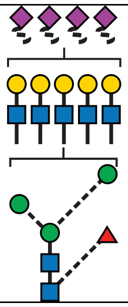	
**XLVI, 66**				**XLVII, 69**	
$H_7N_7F_1N5A_4$				$H_8N_7F_1N5A_4$	
3884.3				4046.4	
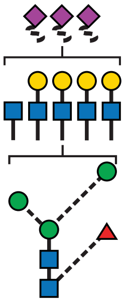	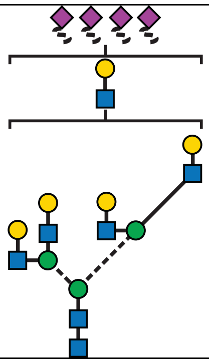	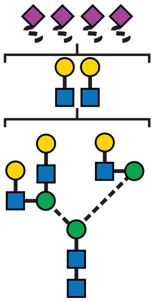	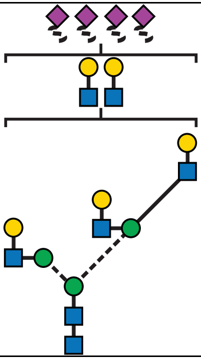	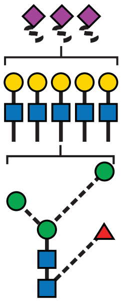	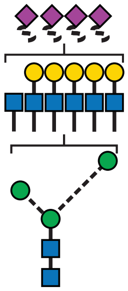
**XLVI, 65**	**LVII, 108**	**LIX, 108**	**LX, 108**	**XLVII, 68**	**XLVIII, 109**
$H_7N_7F_1N5A_3$	$H_8N_7N5A_4$	$H_8N_7N5A_4$	$H_8N_7N5A_4$	$H_8N_7F_1N5A_3$	$H_8N_8N5A_4$
3593.3	3900.4	3900.4	3900.4	3755.3	4103.4
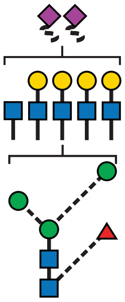	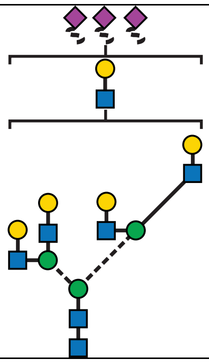	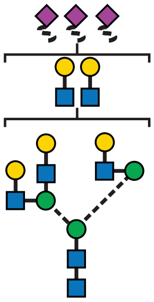	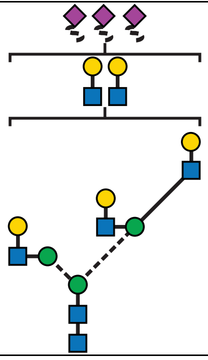	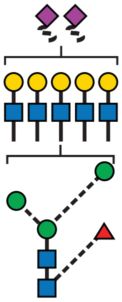	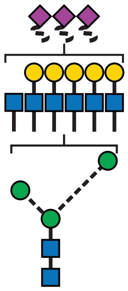
**XLVI, 64**	**LVIII, 107**	**LIX, 107**	**LX, 107**	**XLVII, 67**	**XLVIII, 71**
$H_7N_7F_1N5A_2$	$H_8N_7N5A_3$	$H_8N_7N5A_3$	$H_8N_7N5A_3$	$H_8N_7F_1N5A_2$	$H_8N_8N5A_3$

3302.2	3609.3	3609.3	3609.3	3464.2	3812.3
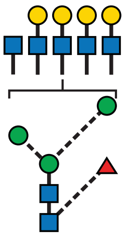	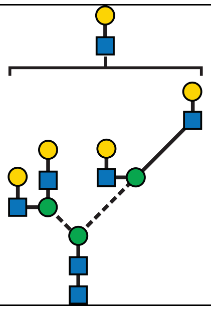	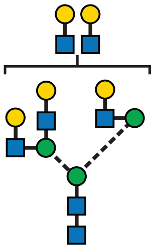	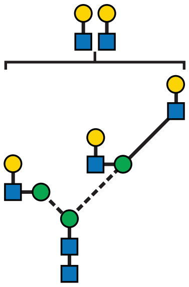	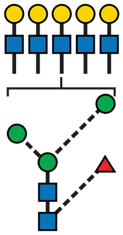	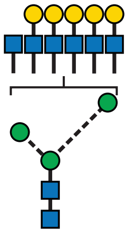
**XLVI**	**LVIII**	**LIX**	**LX**	**XLVII**	**XLVIII**
$H_7N_7F_1$	H_8N_7	H_8N_7	H_8N_7	$H_8N_7F_1$	H_8N_8
2720.0	2736.0	2736.0	2736.0	2882.0	2939.1

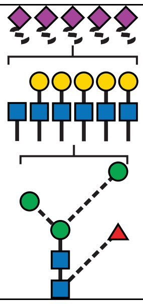					
**XLIX, 75**					
$H_8N_8F_1N5A_5$					
4540.6					
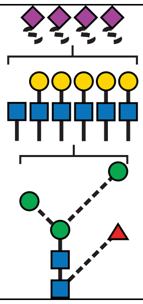				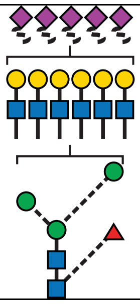	
**XLIX, 74**				**L, 78**	
$H_8N_8F_1N5A_4$				$H_9N_8F_1N5A_5$	
4249.5				4702.6	
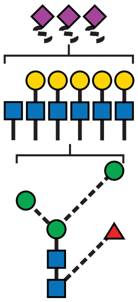	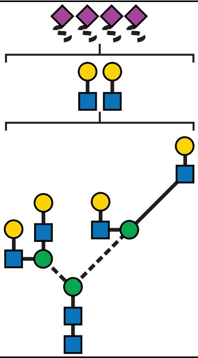	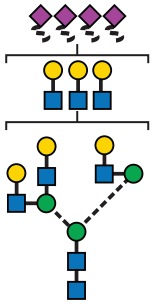	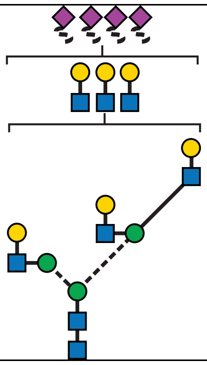	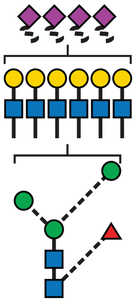	
**XLIX, 73**	**LXI, 111**	**LXII, 111**	**LXIII, 111**	**L, 77**	
$H_8N_8F_1N5A_3$	$H_9N_8N5A_4$	$H_9N_8N5A_4$	$H_9N_8N5A_4$	$H_9N_8F_1N5A_4$	
3958.4	4265.5	4265.5	4265.5	4411.5	
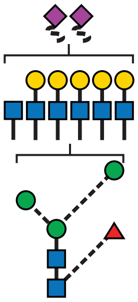	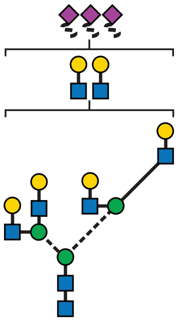	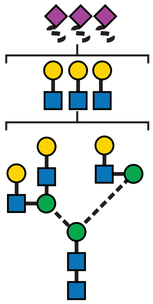	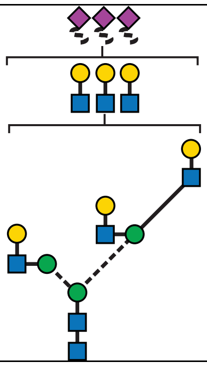	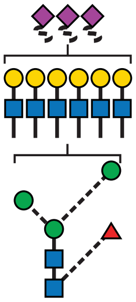	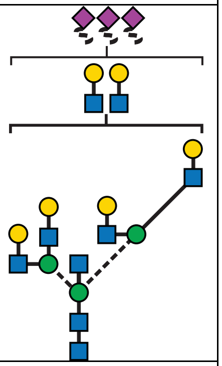
**XLIX, 72**	**LXI, 110**	**LXII, 110**	**LXIII, 110**	**L, 76**	**LXIV, 112**
$H_8N_8F_1N5A_2$	$H_9N_8N5A_3$	$H_9N_8N5A_3$	$H_9N_8N5A_3$	$H_9N_8F_1N5A_3$	$H_9N_9\ N5A_3$
3667.3	3974.4	3974.4	3974.4	4120.4	4177.5

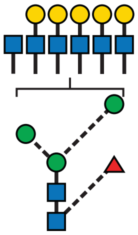	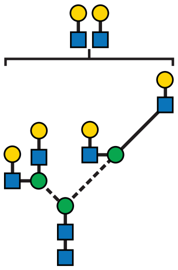	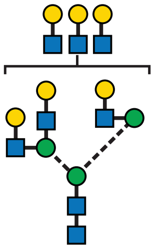	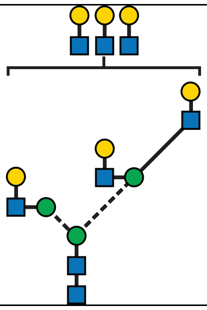	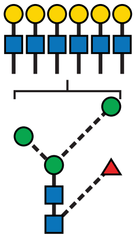	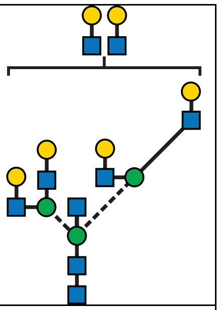
**XLIX**	**LXI**	**LXII**	**LXIII**	**L**	**LXIV**
$H_8N_8F_1$	H_9N_8	H_9N_8	H_9N_8	$H_9N_8F_1$	H_9N_9
3085.1	3101.1	3101.1	3101.1	3247.2	3304.2

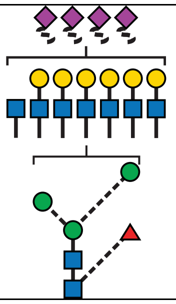					
**LI, 80**					
$H_9N_9F_1N5A_4$					
4614.6					
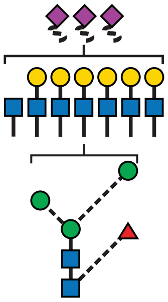	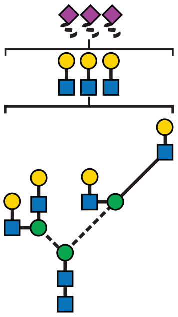	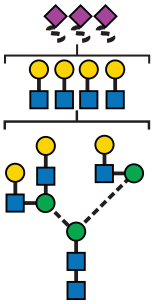	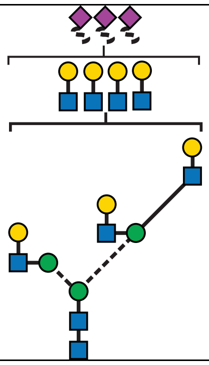	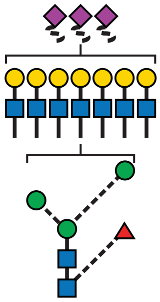	
**LI, 79**	**LXVII, 113**	**LXVIII, 113**	**LXIX, 113**	**LII, 81**	
$H_9N_9F_1N5A_3$	$H_{10}N_9N5A_3$	$H_{10}N_9N5A_3$	$H_{10}N_9N5A_3$	$H_{10}N_9F_1N5A_3$	
4323.5	4339.5	4339.5	4339.5	4485.6	
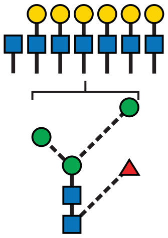	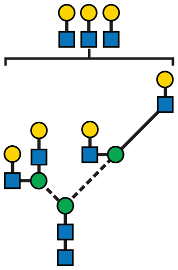	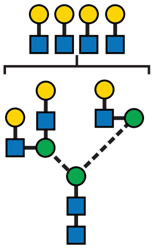	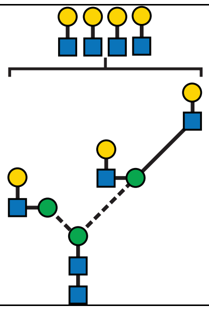	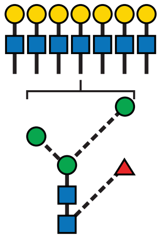	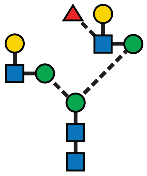
**LI**	**LXVII**	**LXVIII**	**LXIX**	**LII**	**LXXI**
$H_9N_9F_1$	$H_{10}N_9$	$H_{10}N_9$	$H_{10}N_9$	$H_{10}N_9F_1$	$H_5N_4F_1$
3450.3	3466.3	3466.3	3466.3	3612.3	
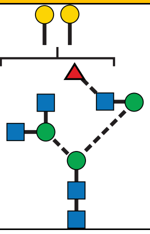	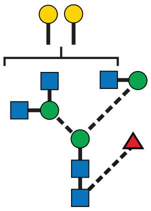	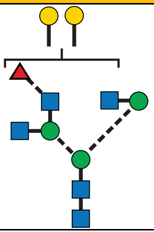	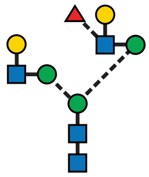	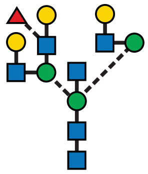	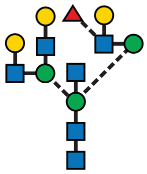
**LXXII**	**LXXIII**	**LXXIV**	**LXXV**	**LXXVI**	**LXXVII**
$H_5N_6F_1$	$H_5N_6F_1$	$H_5N_6F_1$	$H_4N_5F_1$	$H_6N_5F_1$	$H_6N_5F_1$

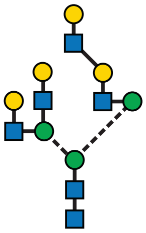	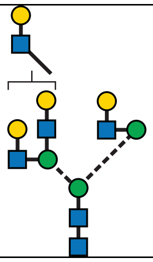	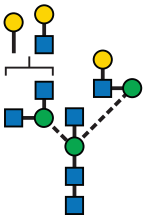	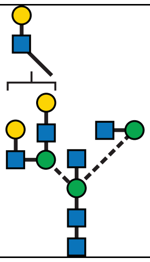	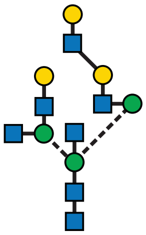	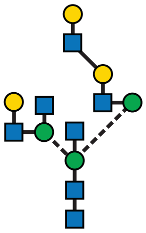
**LXXVIII**	**LXXIX**	**LXXX**	**LXXXI**	**LXXXII**	**LXXXIII**
H_7N_6	H_7N_6	H_6N_7	H_6N_7	H_6N_7	H_6N_7
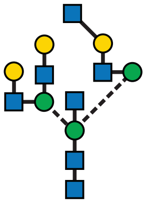	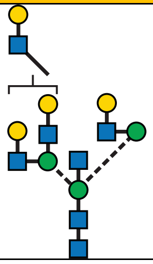	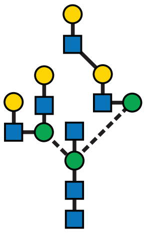			
**LXXXIV**	**LXXXV**	**LXXXVI**			
H_6N_7	H_7N_7	H_7N_7			

[1] 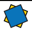 = ambiguous, either bisecting GlcNAc or antenna GalNAc

FSH Glycoform αAsn^{52} glycan analysis

Analysis of αAsn^{52} oligosaccharides from selected FSH glycoform fractions 9, 12, 21, 22, 30 and 32 from the Superdex 75 chromatogram in Fig. 4 by nano-ESI-ion mobility mass spectrometry is shown below in Figs. S5-S8. Glycan ions are listed in associated Excel Tables S15-S17, respectively. Glycan abundance ata are summarized in the accompanying publication in Figs. 6 and 7, as well as in Table S18.

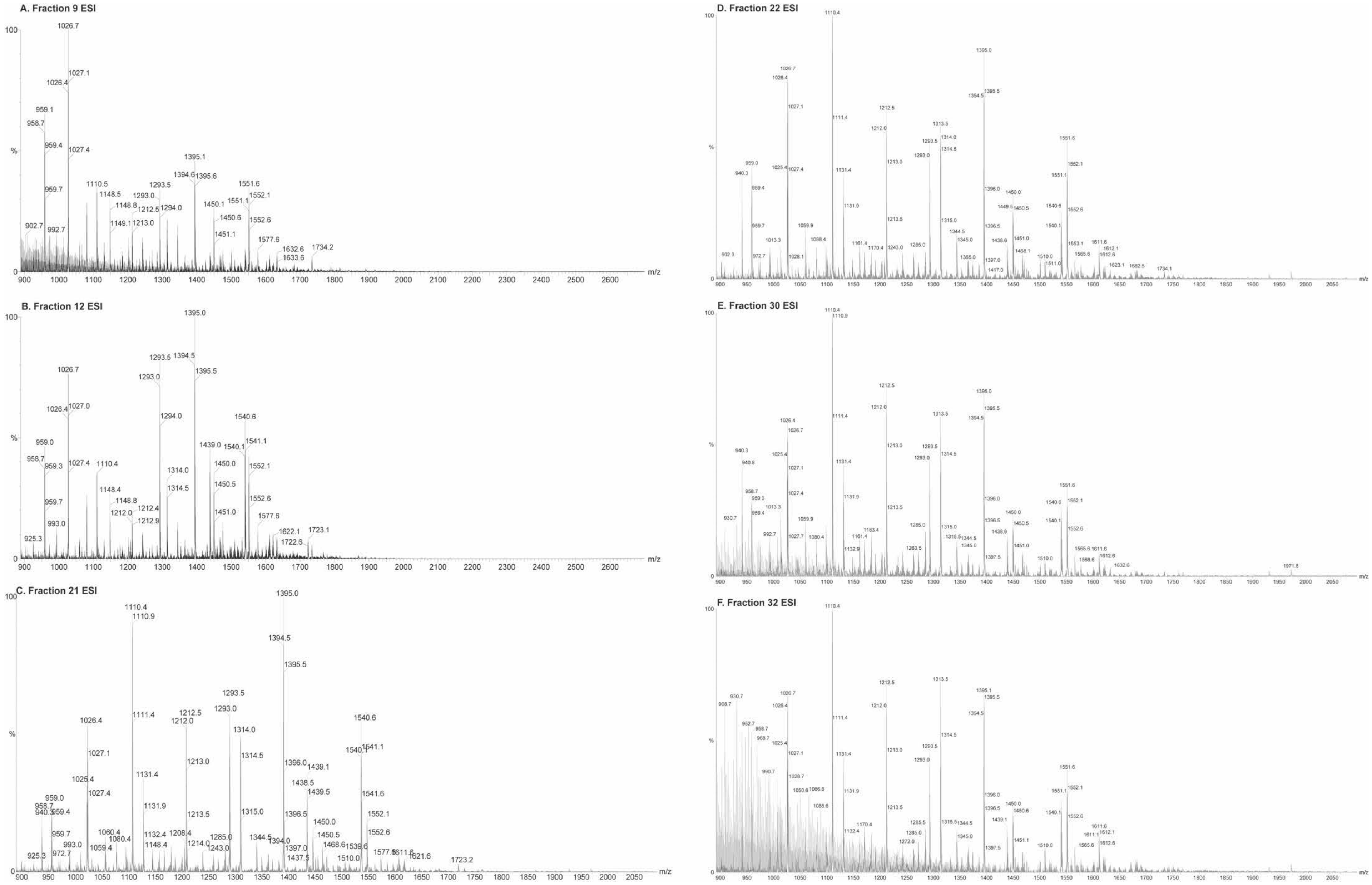

Figure S5. Mass spectrometry of hFSHα Asn[52] glycans. Oligosaccharides were selectively released from dissociated subunits in Superdex 75 column fractions 9, 12, 21, 30 and 32 by PNGaseF digestion, as shown in Fig. 5. Nano-ESI-MS was used to provide an overview of the ions derived from each glycan sample, as indicated. Singly, doubly, and triply charged glycan ions are documented in Figs. S6-S8, below.

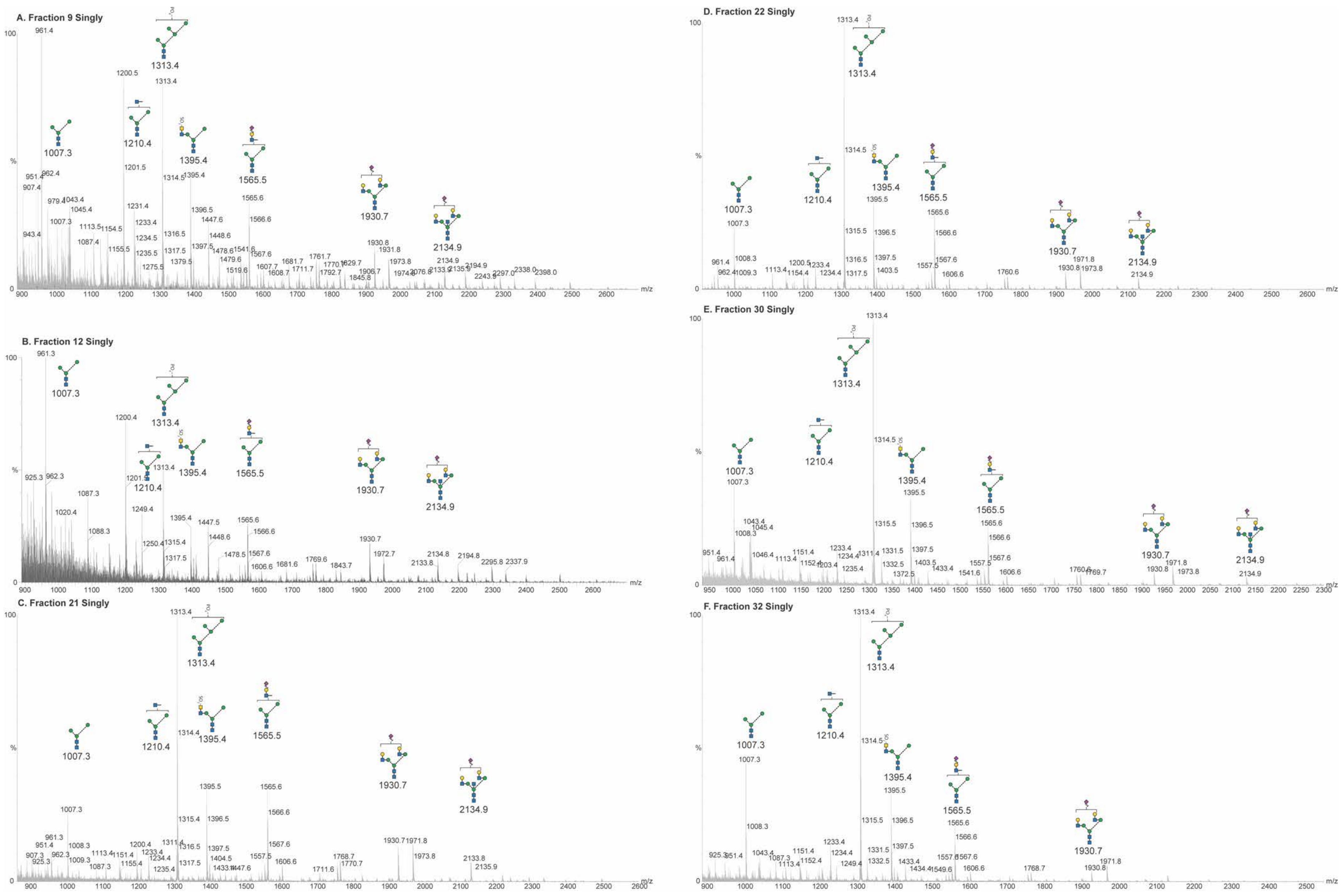

Figure S6. Mass spectrometry of singly charged hFSHα Asn[52] glycans. Oligosaccharides were selectively released from dissociated subunits by PNGaseF digestion, as shown in Fig. 5. Nano-ESI-ion mobility-MS was used to characterize each glycan sample. Singly charged glycans, as indicated. Results are tabulated in supplement Excel Table S15.

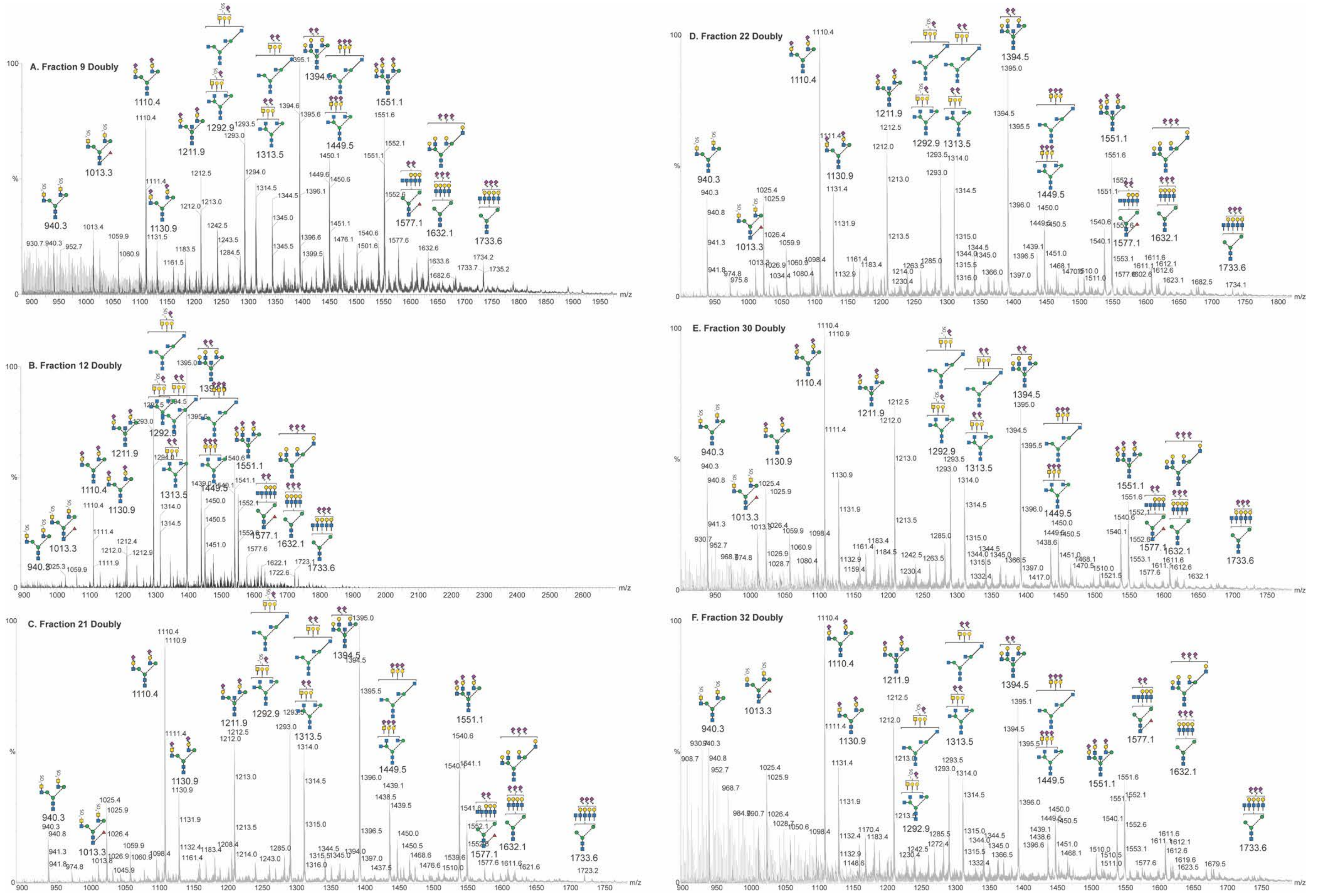

Figure S7. MS analysis of doubly charged hFSH glycoform αAsn[52] oligosaccharide populations. Doubly charged glycans from FSH glycoform purification column fractions, as indicated. Results are tabulated in Excel Table S16.

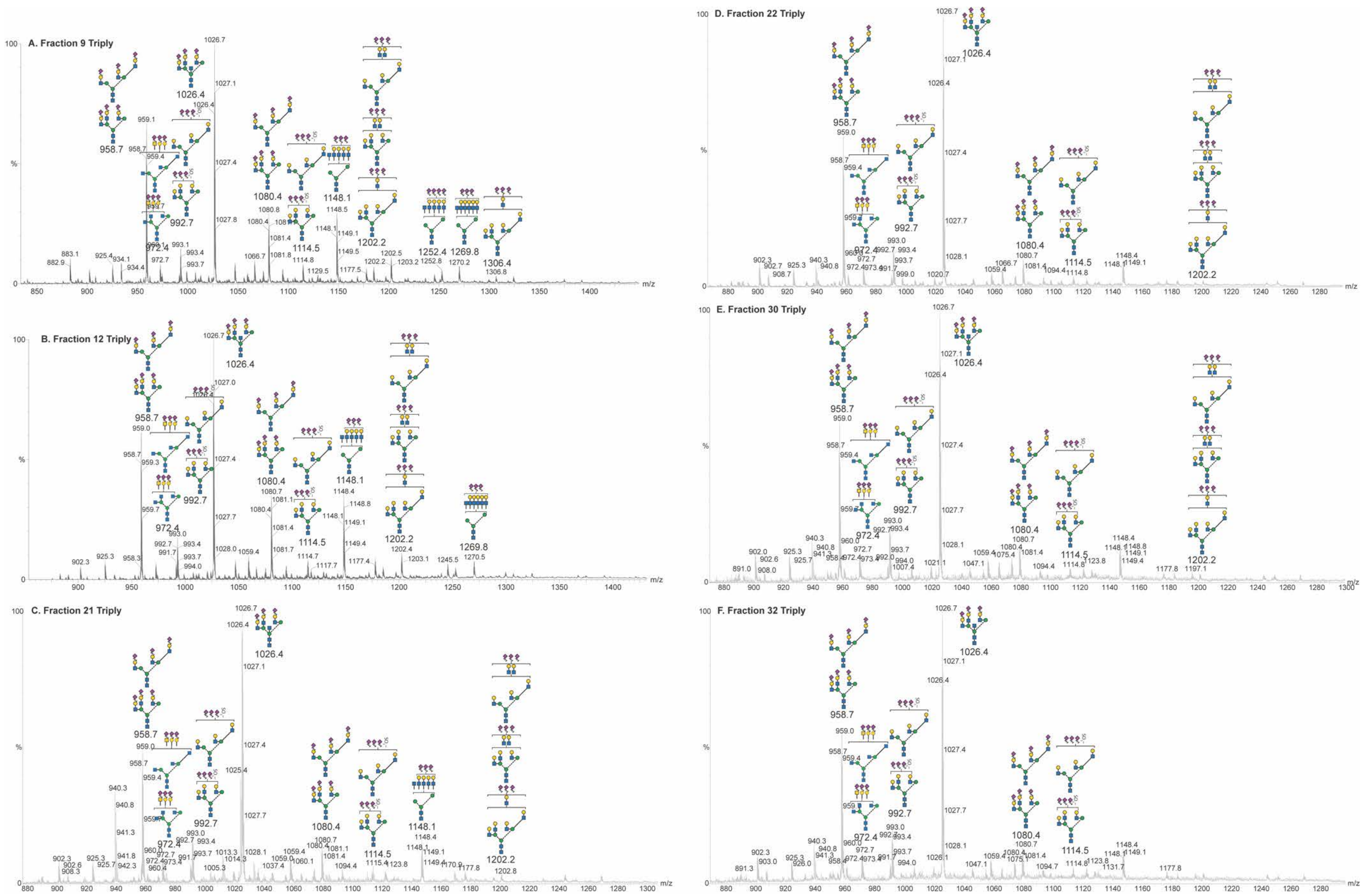

Figure S8. MS analysis of triply charged hFSH glycoform αAsn[52] oligosaccharide populations. Triply charged glycans from FSH glycoform purification column fractions, as indicated. Results are tabulated in Excel Table S17.

Table S18. FSH glycoform αAsn[52] glycans expressed as % of total glycans

Glycan[a]	*m/z*	Composition						Superdex 75 Fraction (Fig. 3A)						Neutral glycan	
		Hex	HexNAc	dHex	Neu5Ac	SO_3H	H_2PO_4	9	12	21	22	30	32	Number[b]	Mass
1	910.3	3	2	0	0	0	0	0.08	0.05	0.16	0.31	0.83	1.07	**LIII**	910.3
2	1234.4	5	2	0	0	0	0	0.02	0.01	0.01	0.02	0.23	0.25	**I**	1234.4
3	1314.4						1	0.24	0.08	0.68	1.55	2.31	2.81		
4	1476.5	6	2	0	0	0	1	0.02	0.01	0.03	0.05	0.09	0.10	**LIV**	1396.5
5	1113.3	3	3	0	0	0	0	0.00	0.00	0.02	0.03	0.11	0.12	**LV**	1113.3
9	1275.4	4	3	0	0	0	0	0.00	0.00	0.02	0.04	0.08	0.08	**III**	1275.5
8	1566.6				1	0	0	0.12	0.05	0.26	0.49	0.58	0.65		
9	1712.6			1	1	0	0	0.03	0.01	0.03	0.05	0.08	0.07	**VI**	1421.5
11	1316.5	3	4	0	0	0	0	0.00	0.00	0.02	0.04	0.09	0.11	**IV, V**	1316.5
13	1396.4					1	0	0.15	0.04	0.24	0.54	0.83	1.00		
15	1542.5			1	0	1	0	0.05	0.01	0.02	0.05	0.10	0.10	**VIII**	1462.5
16	1478.5	4	4	0	0	0	0	0.00	0.01	0.01	0.02	0.03	0.04	**IX**	1478.5
18	1558.5					1	0	0.05	0.02	0.06	0.12	0.18	0.22		
19	1769.6				1	0	0	0.03	0.01	0.08	0.12	0.14	0.14	**XII**	1624.6
20	1849.6					1	0	0.01	0.00	0.01	0.02	0.03	0.02		
21	1931.7	5	4	0	1	0	0	0.07	0.04	0.11	0.15	0.14	0.14	**XIII**	1640.6
24	2011.6					1	0	0.01	0.01	0.01	0.02	0.02	0.01		
30	2222.8				2	0	0	4.07	3.62	9.74	9.41	9.70	9.40		
34	2077.7			1	1	0	0	0.02	0.01	0.01	0.02	0.02	0.03	**XIX**	1786.7
35	2368.8				2	0	0	0.97	0.61	1.10	1.05	1.84	1.63		
37	1519.6	3	5	0	0	0	0	0.00	0.00	0.00	0.00	0.00	0.00	**XI**	1519.6
30	1599.5					1	0	0.00	0.00	0.03	0.05	0.08	0.08		
29	1810.7				1	0	0	0.00	0.00	0.02	0.03	0.03	0.04		
32	1681.6	4	5	0	0	0	0	0.00	0.00	0.01	0.02	0.02	0.03	**XVI, XVII**	1681.6
35	1761.6					1	0	0.05	0.02	0.06	0.11	0.14	0.12		
33	1972.7				1	0	0	0.06	0.02	0.68	0.71	0.93	1.07		
36	2052.7					1	0	1.03	0.56	2.96	3.53	3.77	3.87		
34	2263.8				2	0	0	1.42	0.89	3.80	3.91	4.53	4.49		
39	1907.6			1	0	1	0	0.02	0.01	0.02	0.03	0.05	0.05	**XXI**	1827.7
40	2198.7				1	1	0	0.70	0.32	1.10	0.84	1.99	1.76		
41	1843.7	5	5	0	0	0	0	0.02	0.01	0.05	0.09	0.10	0.10	**XXII**	1843.7
42	2134.7				1	0	0	0.02	0.02	0.07	0.11	0.10	0.10		
44	2214.7					1	0	0.19	0.15	0.59	0.58	0.80	0.84		
43	2425.9				2	0	0	2.77	2.08	6.19	6.07	7.06	6.21		
45	2280.8			1	1	0	0	0.01	0.00	0.01	0.01	0.01	0.01	**XXIV**	

46	2571.9				2	0	0	0.51	0.31	0.58	0.56	0.98	0.82		1989.7
47	2296.8	6	5	0	1	0	0	0.02	0.02	0.02	0.02	0.02	0.02	**XXV, XXVI**	2005.7
50	2376.8					1	0	0.02	0.01	0.01	0.01	0.01	0.01		
48	2587.9				2	0	0	4.11	8.39	6.96	5.02	5.07	4.55		
51	2667.9					1	0	2.34	1.71	1.44	1.87	1.74	1.77		
49	2879.0				3	0	0	12.93	13.82	9.05	9.41	7.75	8.08		
52	2959.0					1	0	1.81	1.41	1.09	1.25	1.16	1.13		
54	2734.0			1	2	0	0	0.89	0.97	0.89	0.82	1.06	0.97	**XXXI, XXXII**	2151.8
55	3025.1				3	0	0	0.65	0.27	0.23	0.22	0.33	0.42		
56	1882.5	3	6	0	0	2	0	1.01	0.27	1.85	3.11	4.08	4.36	**XVIII**	1722.6
57	2093.7				1	1	0	0.24	0.11	0.72	0.47	1.29	0.58		
58	2028.6			1	0	2	0	1.44	0.35	0.84	1.20	2.36	2.08	**XXIII**	1868.7
60	2175.8	4	6	0	1	0	0	0.00	0.00	0.01	0.01	0.01	0.01	**LVI**	1884.7
61	2126.7	5	6	0	0	1	0	0.01	0.01	0.01	0.01	0.01	0.01	**XXIX,XXX**	2046.8
62	2337.8				1	0	0	0.01	0.01	0.02	0.02	0.02	0.01		
65	2417.8					1	0	0.52	0.92	1.39	0.94	1.18	1.18		
63	2628.9				2	0	0	2.98	3.62	5.65	5.10	4.26	4.18		
66	2708.9					1	0	0.83	0.45	0.48	0.67	0.57	0.64		
64	2920.0				3	0	0	2.37	1.97	1.58	1.97	1.72	1.92		
67	2192.8			1	0	0	0	0.01	0.01	0.01	0.01	0.01	0.01	**XXXIII, XXXIV**	2192.8
68	2775.0				2	0	0	0.29	0.26	0.30	0.27	0.29	0.30		
70	2499.9	6	6	0	1	0	0	0.01	0.01	0.01	0.01	0.01	0.01	**XXXV, XXXVI**	2208.8
71	2791.0				2	0	0	5.74	10.55	10.99	8.41	7.31	6.88		
72	3082.1				3	0	0	18.56	19.43	16.19	16.22	11.92	12.87		
73	2646.0			1	1	0	0	0.00	0.00	0.00	0.00	0.00	0.00	**XXXVII, XXXVIII**	2354.9
74	2937.1				2	0	0	1.07	1.22	1.29	1.13	1.21	1.13		
76	2953.0	7	6	0	2	0	0	1.35	1.75	0.83	0.60	0.66	0.68	**XXXIX, XL**	2370.9
77	3244.1				3	0	0	4.95	4.94	1.62	1.35	1.11	1.04		
79	3324.1					1	0	1.14	0.73	0.28	0.33	0.17	0.17		
78	3535.3				4	0	0	3.05	2.23	0.64	0.55	0.26	0.21		
	3681.3			1	4	0	0	0.11	0.03	0.00	0.00	0.00	0.00	**LVII**	2516.9
84	3285.2	6	7	0	3	0	0	0.87	0.55	0.18	0.15	0.15	0.20	**XLI**	2411.9
														**XLIV**	2557.9
86	3156.1	7	7	0	2	0	0	1.36	1.67	0.81	1.49	0.65	0.76	**XLV**	2573.9
87	3447.2				3	0	0	4.84	4.92	1.70	1.52	1.37	1.20		
88	3738.3				4	0	0	2.29	1.40	0.33	0.36	0.21	0.16		
92	3609.3	8	7	0	3	0	0	1.44	0.94	0.16	0.17	0.13	0.11	**LVIII, LIX, LX**	2736.0
93	3900.4				4	0	0	0.54	0.38	0.00	0.00	0.00	0.00		
98	3812.4	8	8	0	3	0	0	0.95	0.64	0.17	0.13	0.09	0.09	**XLVIII**	2939.1
99	4103.4				4	0	0	0.31	0.19	0.00	0.00	0.00	0.00		
104	3974.4	9	8	0	3	0	0	0.36	0.18	0.00	0.00	0.00	0.00		

105	4265.5				4	0	0	0.11	0.03	0.00	0.00	0.00	0.00	**LXI, LXII, LXIII**	3101.1
109	4177.5	9	9	0	3	0	0	0.18	0.10	0.00	0.00	0.00	0.00	**LXIV, LXV, LXVI**	3304.7
112	4339.5	10	9	0	3	0	0	0.08	0.02	0.00	0.00	0.00	0.00	**LXVII, LXVIII, LXIX**	3466.3

[a]Glycan numbers refer to those structures encountered in the initial hFSH analysis and results of glycoform αAsn52 glycans as listed in Table 1.

[b]Neutral glycans indicated by Roman numerals as in Table S13.

FSH αAsn[52] oligosaccharides released from a second human pituitary FSH preparation were characterized by mass spectrometry following desialylation by mild acetic acid hydrolysis. Fragmentation of the neutral compounds is shown below (Fig. S9) with the structure or structures supported by fragmentation shown as an inset. Interestingly, all the glycans in the tetra-antennary oligosaccharide mass range were found to be tri-antennary with lactosamine repeats providing the additional mass (Figs. 9N-P).

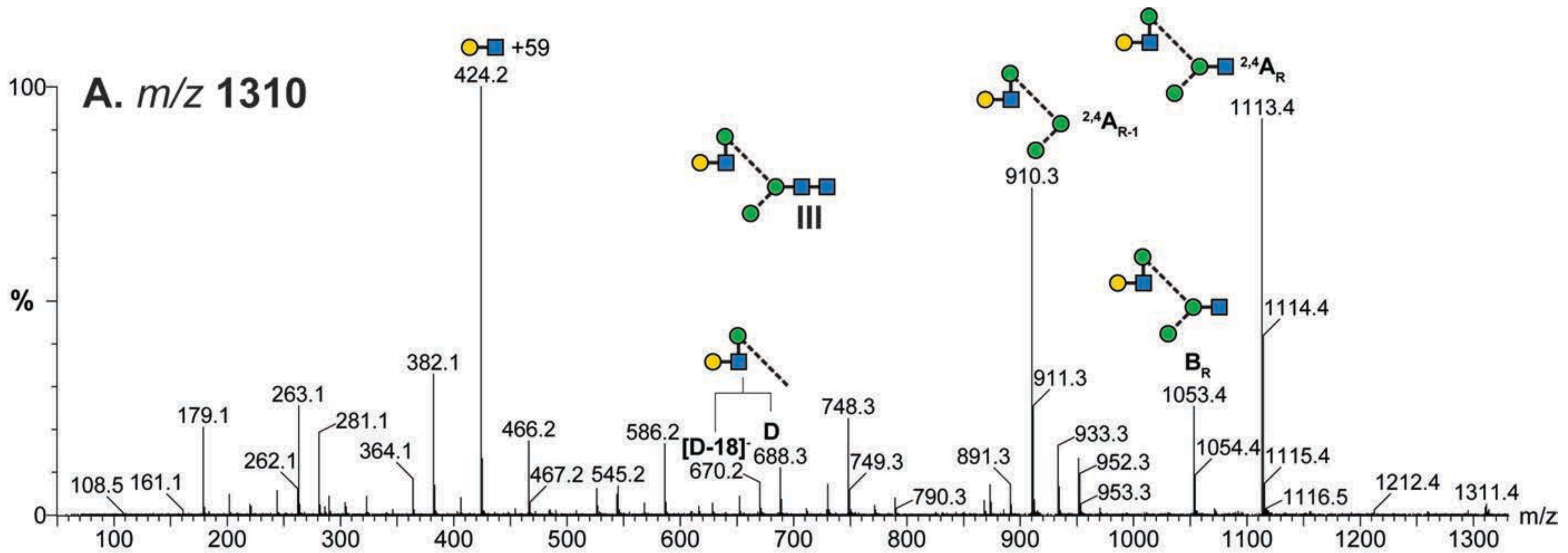

Figure S9A. The negative ion MS/MS spectrum of *m/z* 1310 ($Hex_4HexNAc_3$). Fragment nomenclature followed that of Domon and Costello [4] as modified by Harvey et al. [5; 6]. The spectrum revealed $^{2,4}A_R$, B_R and $^{2,4}A_{R-1}$ ions at *m/z* 1113, 1053 and 910, respectively, consistent with β1-4-linked GlcNAc-GlcNAc core. *m/z* 424 = Gal-GlcNAc+59. *m/z* 688 and 670 = D and [D-18]- ions from 6-antenna. Weak *m/z* 526, no 508. Therefore, substitution in 6-antenna, structure **III** (inset the Roman numerals indicate neutral glycans in Tables S13 or S17).

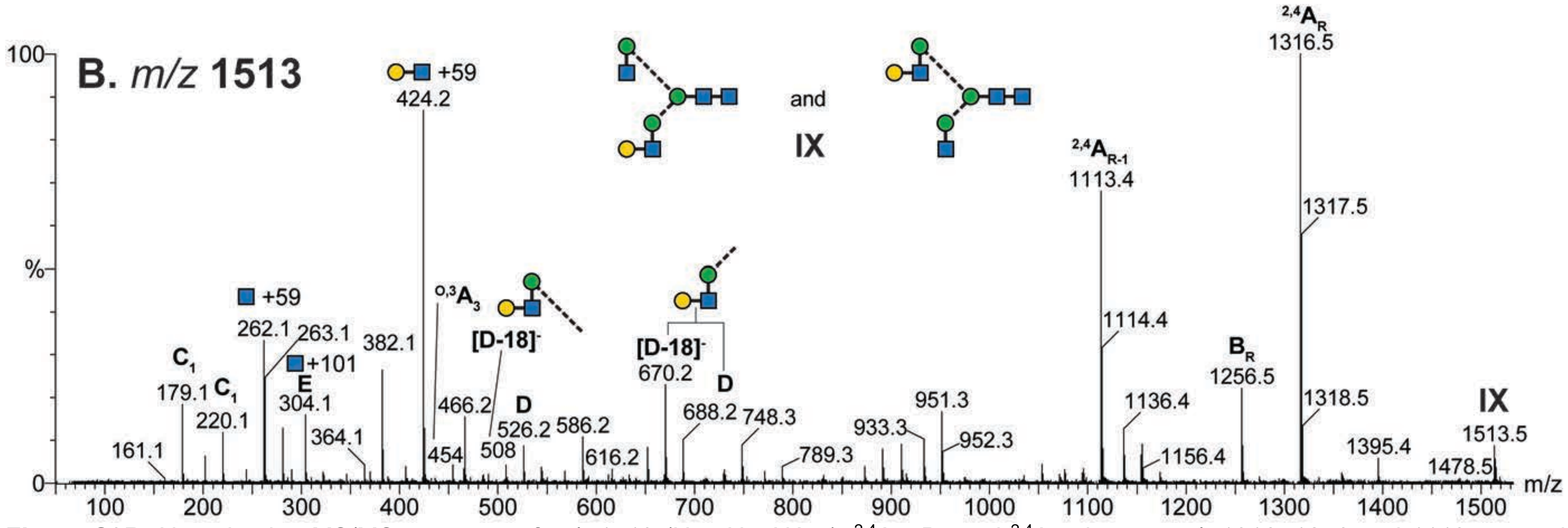

Figure S9B. Negative ion MS/MS spectrum of *m/z* 1513 ($Hex_4HexNAc_4$). $^{2,4}A_R$, B_R and $^{2,4}A_{R-1}$ ions at *m/z* 1316, 1256 and 1113 respectively (1-4-linked GlcNAc-GlcNAc core). C_1 ions at *m/z* 179 and *m/z* 220 showing both hexose and HexNAc at nonreducing terminus. *m/z* 424 = Hex-HexNAc+59. *m/z* 262 = HexNAc+59. D, $[D\text{-}18]^-$ and $^{O,3}A_3$ ions at *m/z* 526, 508 and 454 respectively showing GlcNAc in 6-antenna, also *m/z* 304 = E ion (GlcNAc+101). Therefore, fourth hexose (galactose) must be in the 3-antenna. Second set of more abundant D and [D-18]- ions at *m/z* 688 and 670 showing presence of the isomeric compound with galactose in the 6-antenna. Therefore 2 structures, both alternatives of structure **IX** (inset).

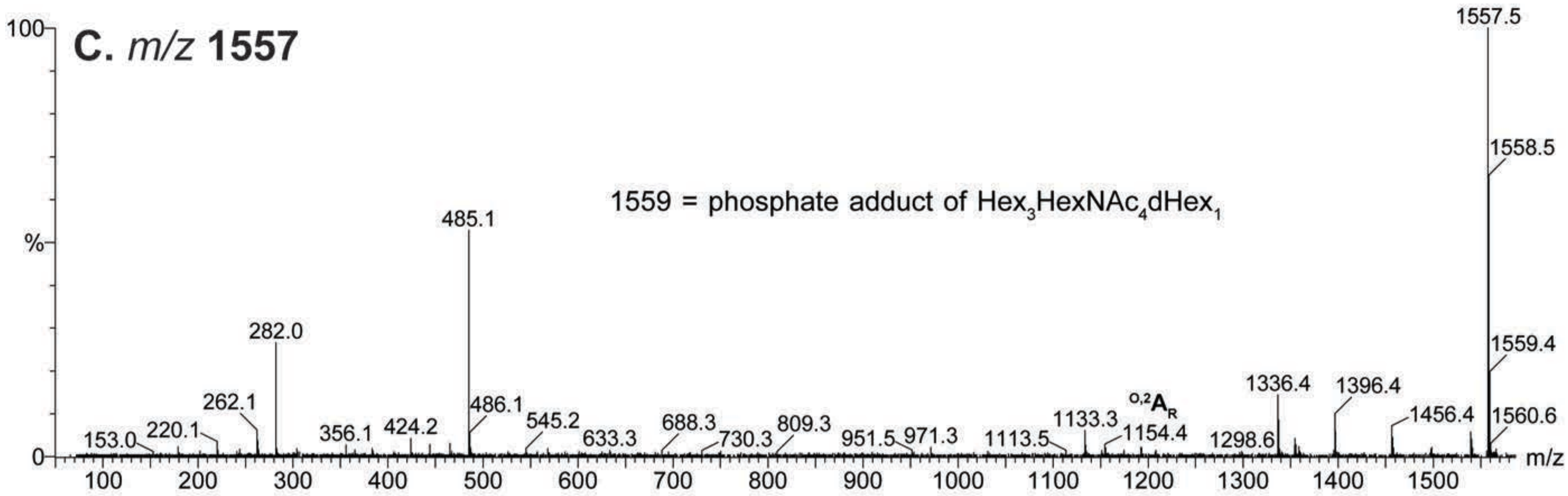

Figure S9C. Negative ion MS/MS spectrum of *m/z* 1557 ($Hex_3HexNAc_4dHex_1$). Very weak spectrum. *m/z* 1559 = phosphate adduct of $Hex_3HexNAc_4dHex_1$. This spectrum contains a weak $^{O,2}A_R$ ion at *m/z* 1154 showing fucose on core GlcNAc. No structure indicated.

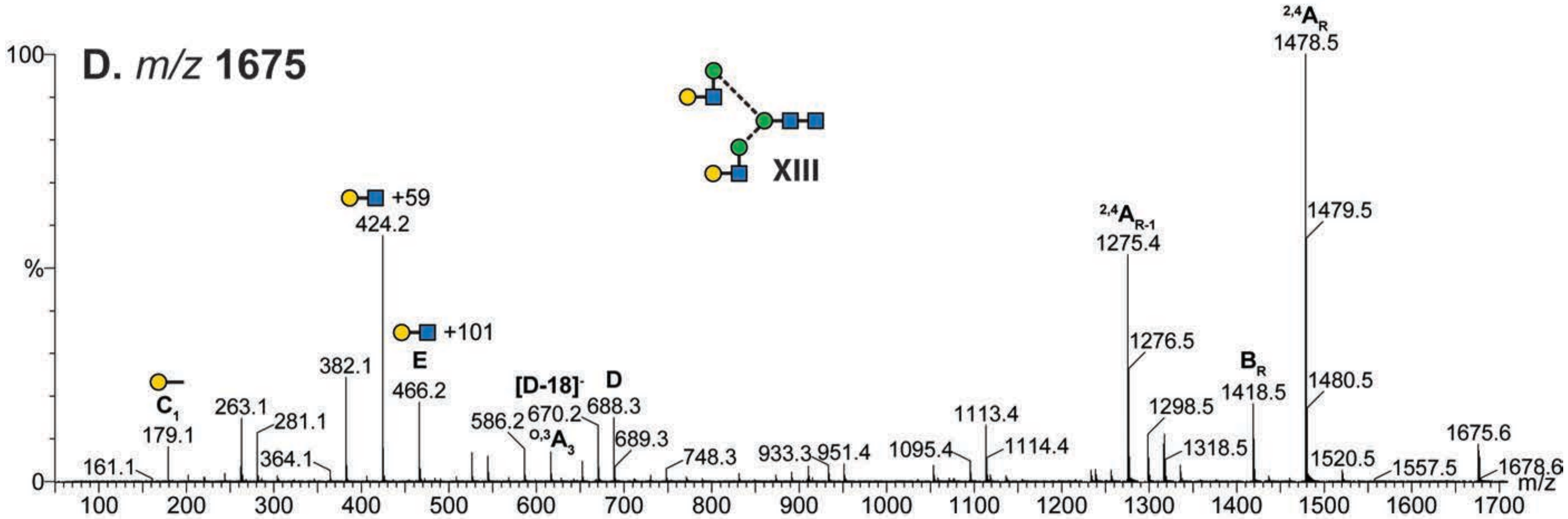

Figure S9D. Negative ion MS/MS spectrum of *m/z* 1675 ($Hex_5HexNAc_4$). $^{2,4}A_R$, B_R and $^{2,4}A_{R-1}$ ions at *m/z* 1478, 1418 and 1275 respectively (1-4-linked GlcNAc-GlcNAc core). C_1 ion at *m/z* 179 showing hexose at non-reducing terminus (No *m/z* 220, therefore, no non-reducing-terminal GlcNAc). *m/z* 424 = Gal-GlcNAc+59. D, [D-18]- and $^{O,3}A_3$ ions at *m/z* 688, 670 and 616 showing Gal-GlcNAc in 6-antenna. *m/z* 466 = E ion (Gal-GlcNAc+101) from either antennae. Therefore, structure **XIII** (inset).

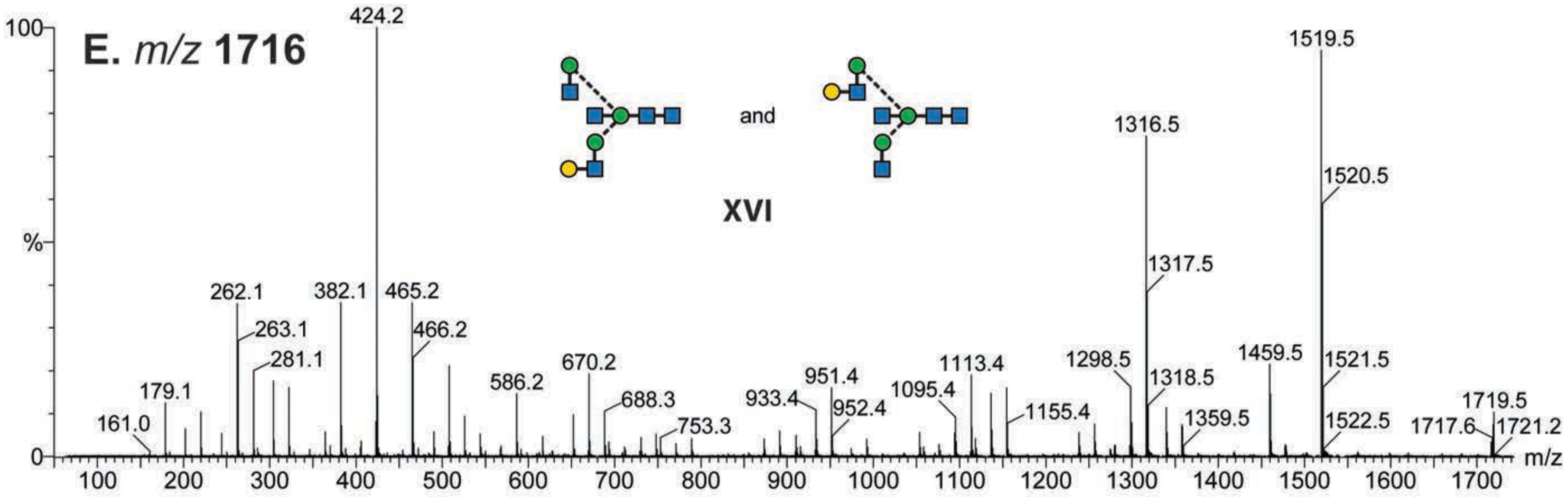

Figure S9E. Negative ion MS/MS spectrum of *m/z* 1716 ($Hex_4HexNAc_5$). Bisected biantennary missing one galactose from 3- and 6-antenna. Both variants of structure **XVI** (inset).

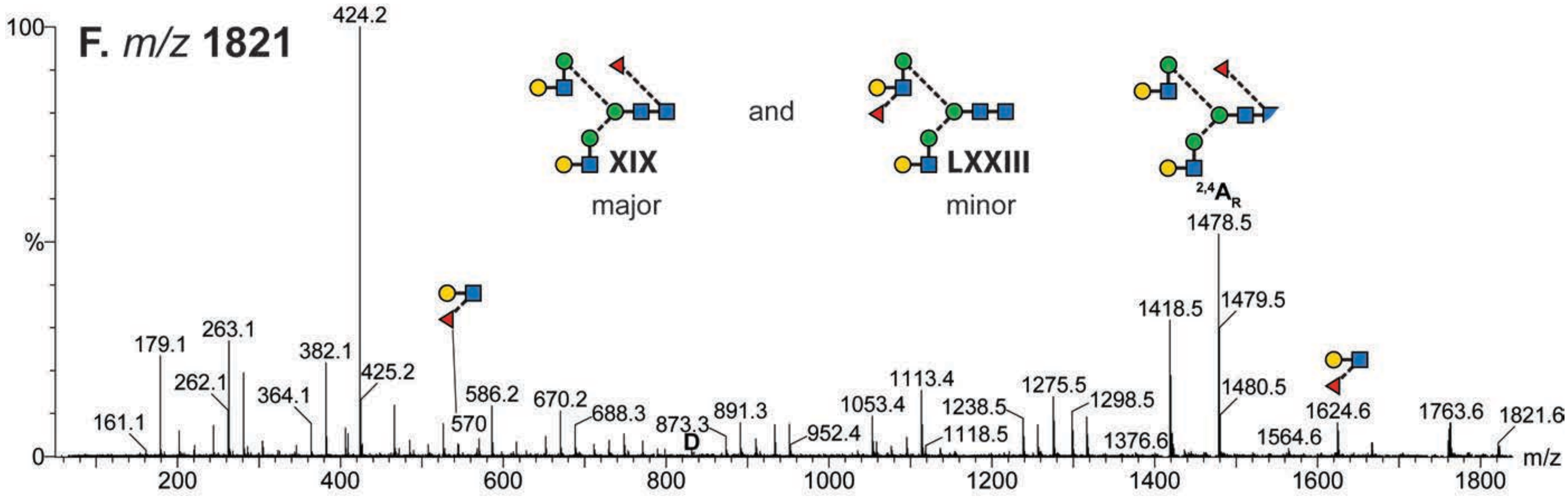

Figure S9F. Negative ion MS/MS spectrum of *m/z* 1821 ($Hex_5HexNAc_4dHex_1$). Mainly core fucosylated ($^{2,4}A_R$ ion at *m/z* 1478) biantennary with very small amount of antenna fucosylation (*m/z* 1624, 570) at least on 6-antenna (D ion at *m/z* 834). Structures **XIX** and **LXXIII** (inset).

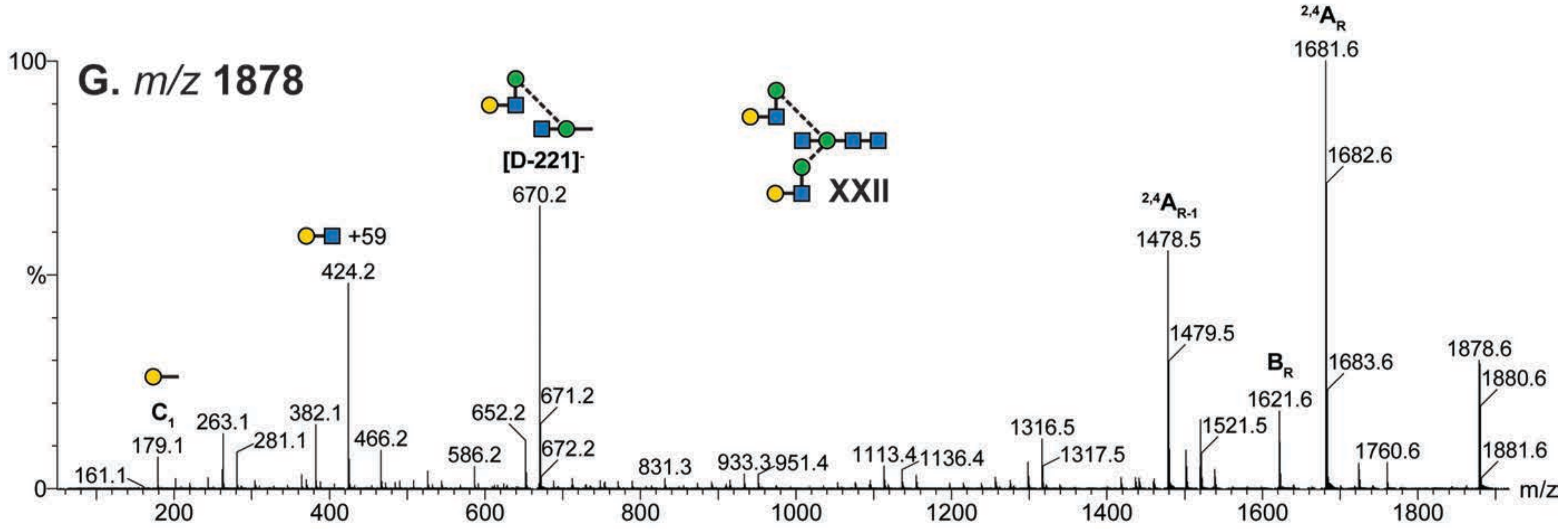

Figure S9G. Negative ion MS/MS spectrum of *m/z* 1878 ($Hex_5HexNAc_5$). $^{2,4}A_R$, B_R and $^{2,4}A_{R-1}$ ions at *m/z* 1681, 1621 and 1478 respectively (1-4-linked GlcNAc-GlcNAc core). C_1 ion at *m/z* 179 showing hexose at non-reducing terminus. *m/z* 424 = Hex-HexNAc. No D ion but abundant [D-221]- ion at *m/z* 670 showing presence of a bisect and Gal-GlcNAc in 6-antenna. Therefore structure **XXII** (inset).

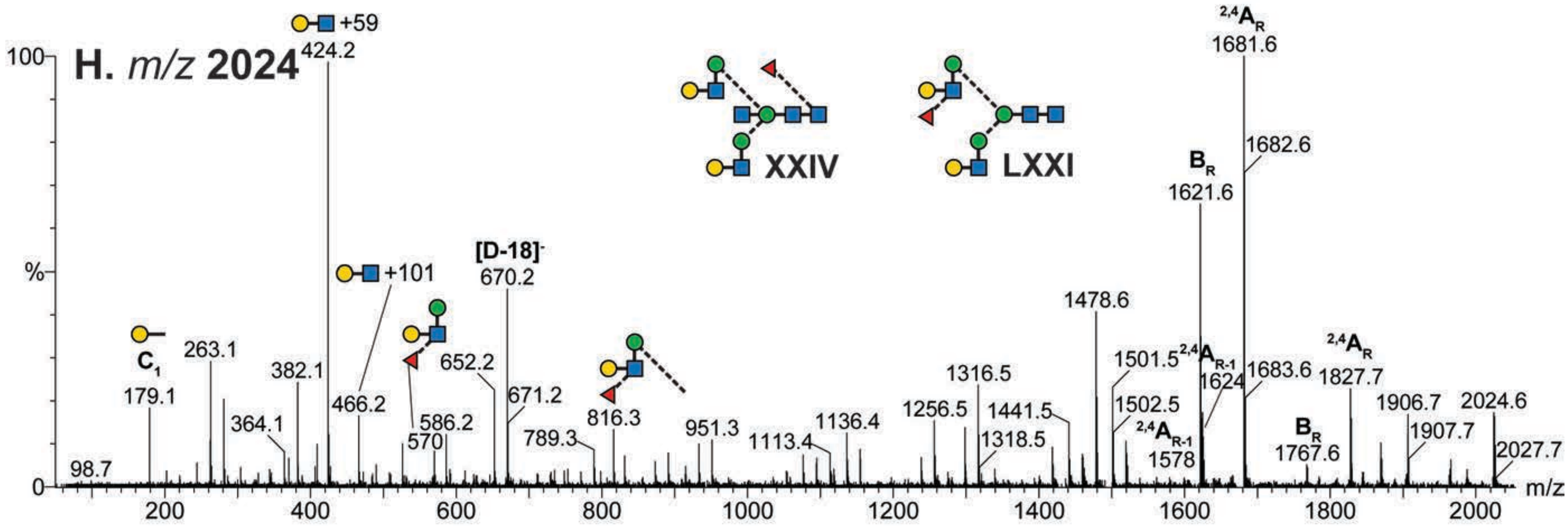

Figure S9H. Negative ion MS/MS spectrum of *m/z* 2024 ($Hex_5HexNAc_5dHex_1$). Two groups of $^{2,4}A_R$, B_R and $^{2,4}A_{R-1}$ ions at *m/z* 1827, 1767 and 1624 and at *m/z* 1681, 1621 and 1578, respectively, showing that fucose is attached to 6-position of reducing-terminal GlcNAc (2nd set of ions) or in an antenna. C_1 ion at *m/z* 179 showing hexose at non-reducing terminus (No *m/z* 220, therefore no non-reducing-terminal GlcNAc). *m/z* 424 = Gal-GlcNAc+59 and at *m/z* 570 (Fucose in an antenna). No D ion but abundant [D-18]- ion at *m/z* 670 showing bisect and Glc-GlcNAc in 6-antenna. *m/z* 466 = E ion (Gal-GalNAc+101). Weak ions at *m/z* 816 = *m/z* 670 + 146 showing fucose in the 6-antenna. Therefore, structure **XXIV** or **LXXI** (inset).

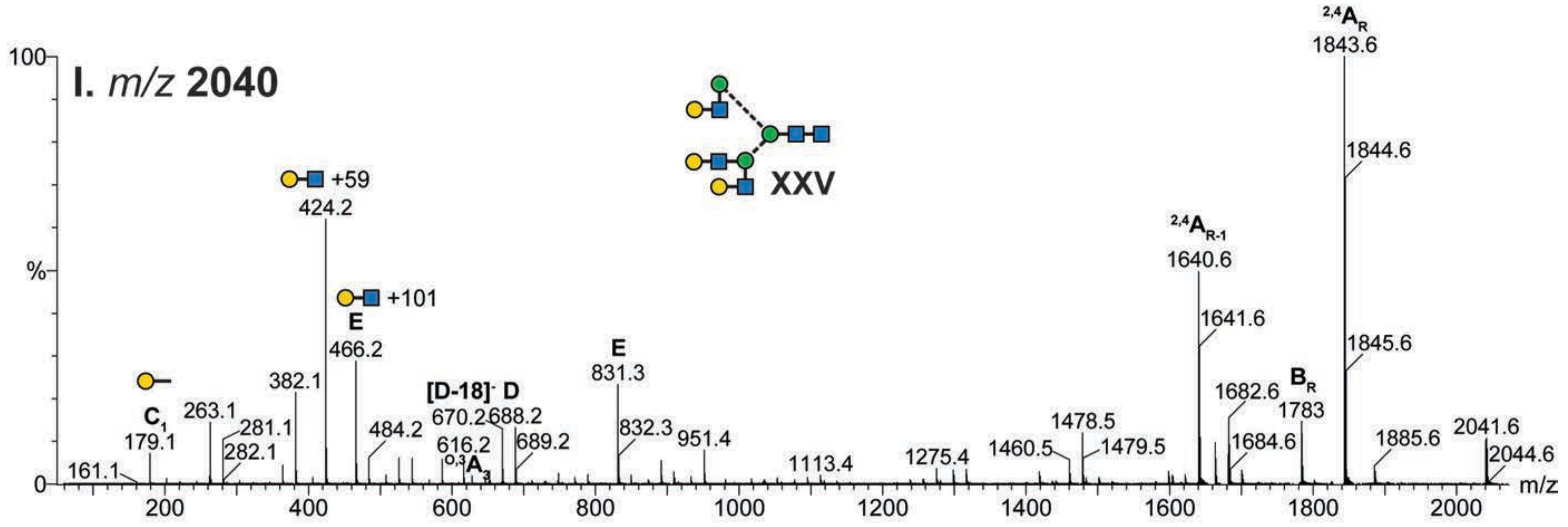

Figure S9I. Negative ion MS/MS spectrum of *m/z* 2040 ($Hex_6HexNAc_5$). $^{2,4}A_R$, B_R and $^{2,4}A_{R-1}$ ions at *m/z* 1843, 1783 and 1640 respectively (1-4-linked GlcNAc-GlcNAc core). C_1 ion at *m/z* 179 showing hexose at non-reducing terminus (No *m/z* 220, therefore, no non-reducing-terminal GlcNAc). *m/z* 424 = Gal-GlcNAc+59. D, [D-18]- and $^{O,3}A_3$ ions at *m/z* 688, 670 and 616 showing Gal-GlcNAc in 6-antenna. *m/z* 466 = E ion (Gal-GlcNAc+101) from 6- antennae, *m/z* 831 = E ion from 3-antenna = *m/z* 466 + 365 (Gal-GlcNAc). Therefore, structure **XXV** (inset).

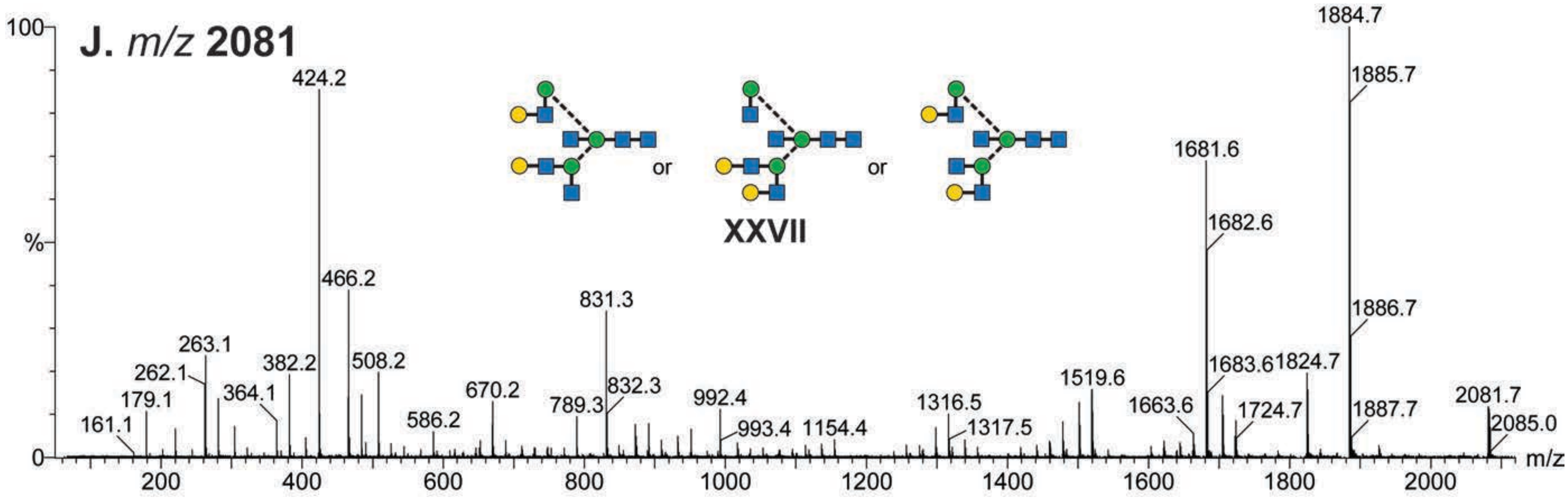

Figure S9J. Negative ion MS/MS spectrum of *m/z* 2081 ($Hex_5HexNAc_6$). Spectrum of bisected triantennary glycans with one galactose missing from any antenna. Therefore, structure **XXVII** (inset).

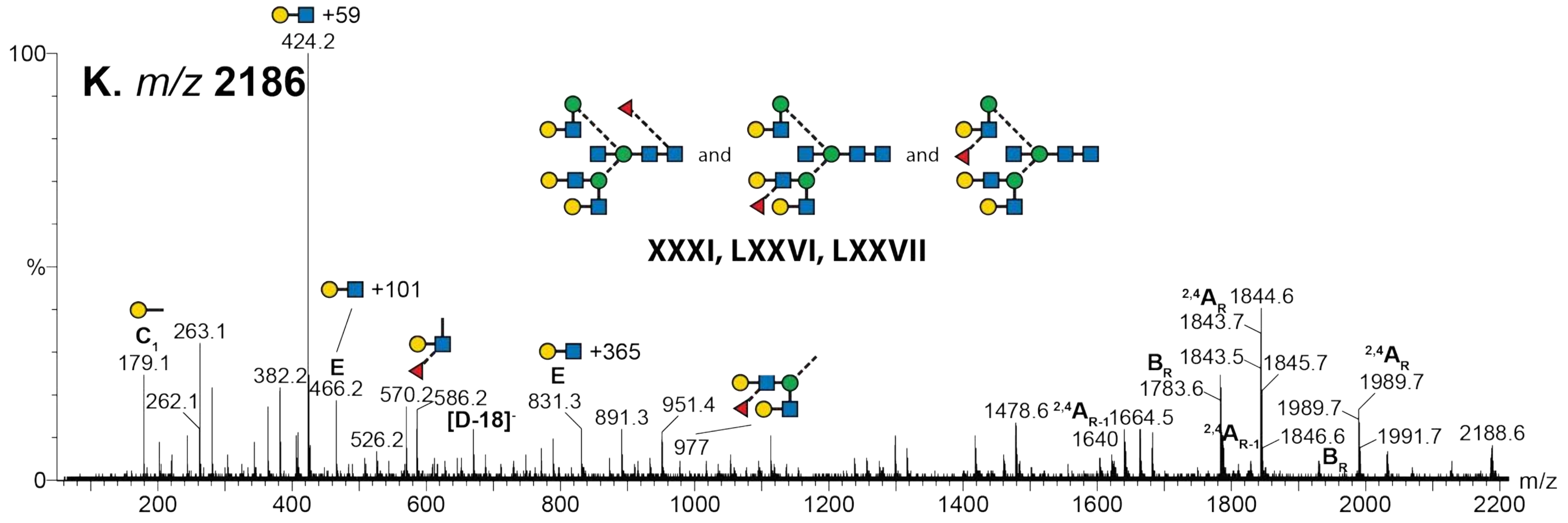

Figure S9K. Negative ion MS/MS spectrum of *m/z* 2186 ($Hex_6HexNAc_5dHex_1$). Two groups of $^{2,4}A_R$, B_R and $^{2,4}A_{R-1}$ ions at *m/z* 1989, 1929 and 1786 and at *m/z* 1843, 1783 and 1640, respectively, showing that fucose is attached to 6-position of reducing-terminal GlcNAc (2nd set of ions) or in an antenna. C_1 ion at *m/z* 179 showing hexose at non-reducing terminus (No *m/z* 220, therefore no non-reducing-terminal GlcNAc). *m/z* 424 = Gal-GlcNAc+59 and at *m/z* 570 (Fucose in an antenna). No D ion but abundant [D-18]- ion at *m/z* 670 showing bisect and Gal-GlcNAc in 6-antenna. *m/z* 466 = E ion (Gal-GlcNAc+101) from 6-antennae, *m/z* 831 = E ion from 3-antenna = *m/z* 466 + 365 (Gal-GlcNAc). Weak ions at *m/z* 977 = *m/z* 831 + 146 showing fucose in the 3-antenna and 688+146 = 834 showing fucose in either antenna. Therefore, structure **XXXI** (inset).

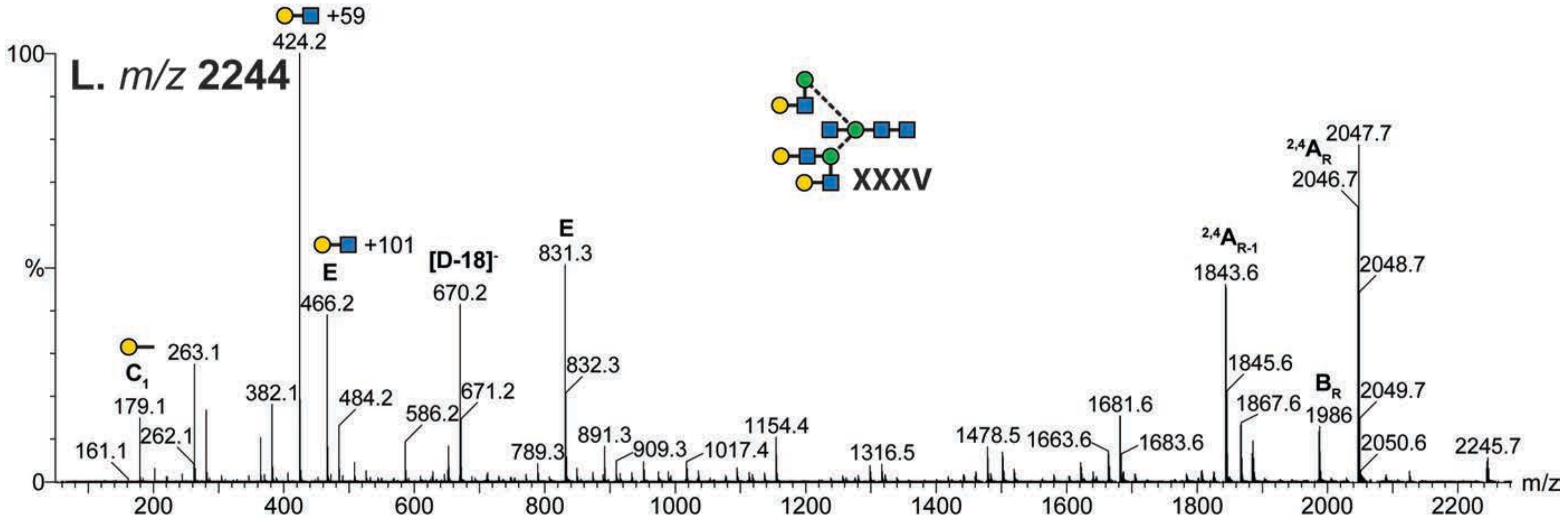

Figure S9L. Negative ion MS/MS spectrum of *m/z* 2244 ($Hex_6HexNAc_6$). $^{2,4}A_R$, B_R and $^{2,4}A_{R-1}$ ions at *m/z* 2046, 1986 and 1843 respectively (1-4-linked GlcNAc-GlcNAc core). C_1 ion at *m/z* 179 showing hexose at non-reducing terminus (No *m/z* 220, therefore no non-reducing-terminal GlcNAc). *m/z* 424 = Gal-GlcNAc+59. No D ion but abundant [D-18]- ion at *m/z* 670 showing bisect and Gal-GlcNAc in 6-antenna. *m/z* 466 = E ion (Gal-GlcNAc+101) from 6- antennae, *m/z* 831 = E ion from 3-antenna = *m/z* 466 + 365 (Gal-GlcNAc). Therefore, structure, **XXXV** (inset).

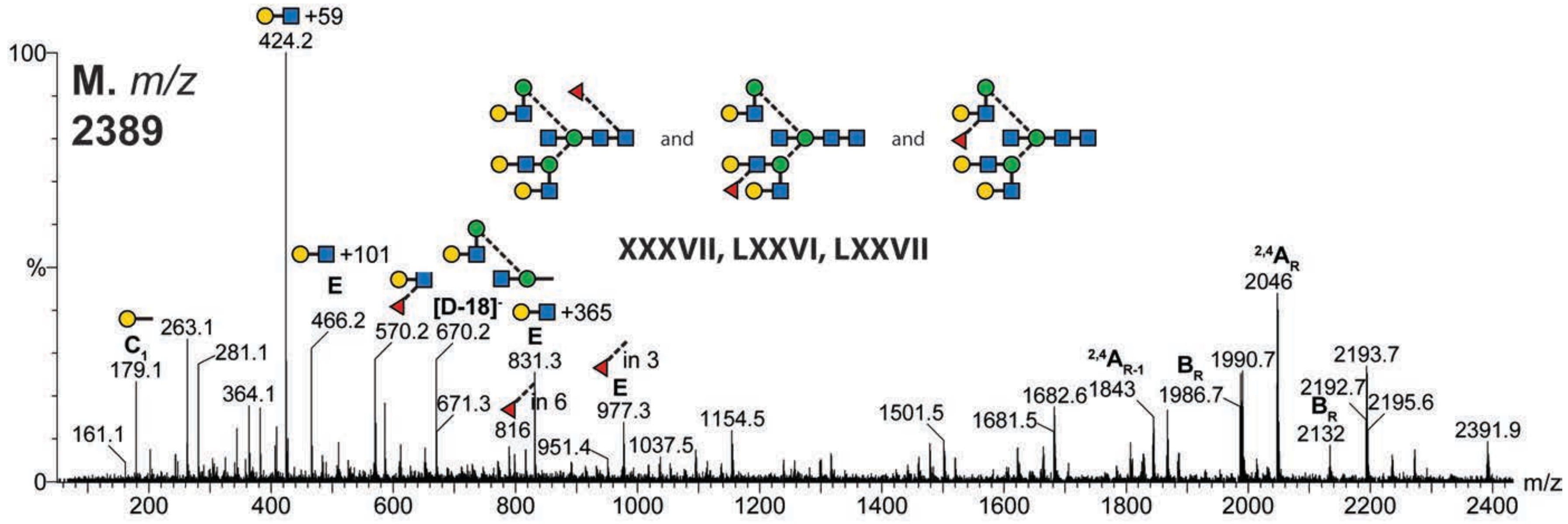

Figure S9M. Negative ion MS/MS spectrum of *m/z* 2391 ($Hex_6HexNAc_6dHex_1$). Group of peaks - focused on isotope. Two groups of $^{2,4}A_R$, B_R and $^{2,4}A_{R-1}$ ions at *m/z* 2192, 2132 and 9893 and at *m/z* 2046, 1986 and 1843 respectively showing that fucose is attached to 6-position of reducing-terminal GlcNAc (2nd set of ions) or in an antenna. C_1 ion at *m/z* 179 showing hexose at non-reducing terminus (No *m/z* 220, therefore, no non-reducing terminal GlcNAc). *m/z* 424 = Gal-GlcNAc+59 and at *m/z* 570 (Fucose in an antenna). No D ion but abundant [D-18]- ion at *m/z* 670 showing bisect and Gal-GlcNAc in 6-antenna. *m/z* 466 = E ion (Gal-GalNAc+101) from 6- antennae, *m/z* 831 = E ion from 3-antenna = *m/z* 466 + 365 (Gal-GlcNAc). *m/z* 977 = *m/z* 831 + 146 showing fucose in the 3-antenna. Weak ion at *m/z* 816 (m/z 670 + 146) showing fucose in the 6-antenna also. Therefore, structures **XXXV, LXXVI, LXXVII** (inset).

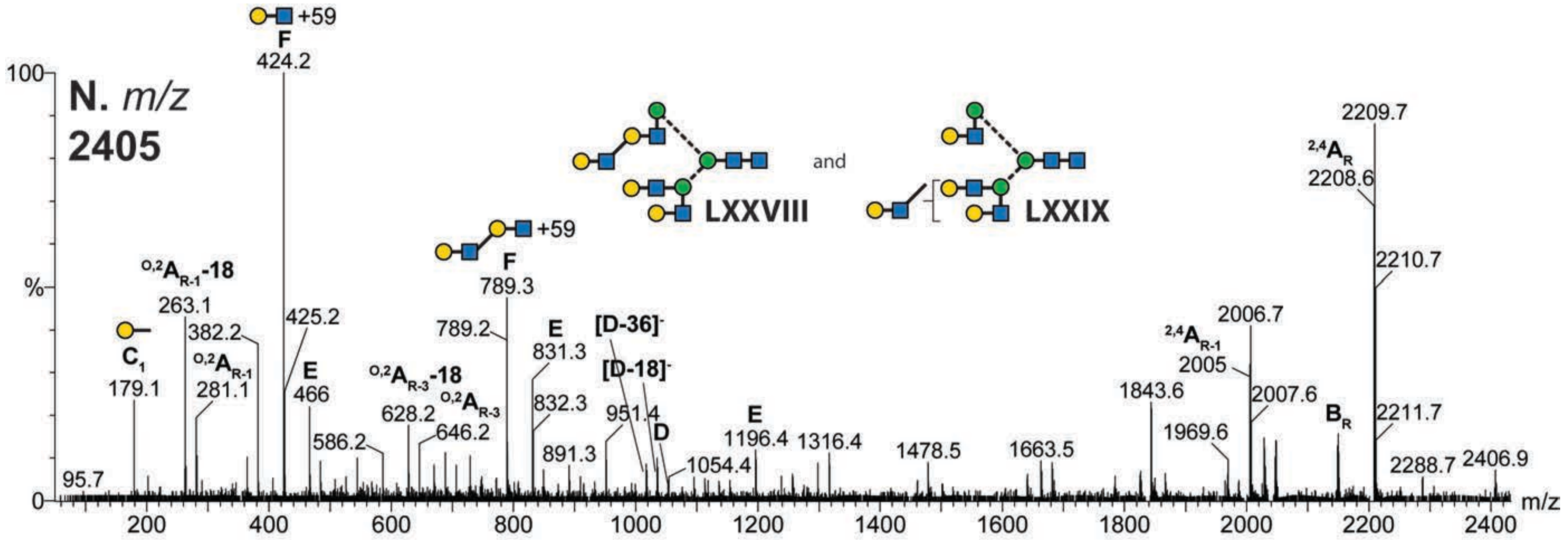

Figure S9N. Negative ion MS/MS spectrum of *m/z* 2406 ($Hex_7HexNAc_6$). Mixture. $^{2,4}A_R$, B_R and $^{2,4}A_{R-1}$ ions at *m/z* 2208, 2148 and 2005 respectively (1-4-linked GlcNAc-GlcNAc core). C_1 ion at *m/z* 179 showing hexose at non-reducing terminus (No *m/z* 220, therefore no non-reducing-terminal GlcNAc). F ion at *m/z* 424 = Gal-GlcNAc+59. Second F ion at *m/z* 789 = 424 + 365 (Gal-GlcNAc-Gal-GlcNAc+59). D, [D-18]- and [D-36]- ions at *m/z* 1053, 1035 and 1017 respectively showing Glc2-GlcNAc2 in 6-antenna. *m/z* 281 = O,2AR-1 (Gal+119) and *m/z* 263 = this ion - 18. The ions at *m/z* 646 and 628 are the corresponding $^{O,2}A_{R-3}$ and $^{O,2}A_{R-3}$ - 18 ions from the N-acetyl-lactosamine extensions. *m/z* 466 = E ion (Gal-GalNAc+101) from 6- antennae, *m/z* 831 = E ion from 3-antenna = *m/z* 466 + 365 (Gal-GlcNAc). *m/z* 1196 = *m/z* 831 + 367, therefore N-acetyl-lactosamine extension also in the 3-antenna. The sample contained no tetra-antennary glycans with N-acetyllactosamine extensions, therefore, these compounds appeared to be tri-antennary glycans with N-acetyl-lactosamine extensions on either the 3- or 6-antennae. Therefore, structures **LXXXI, LXXXII, LXXXIII, LXXXIV, LXXXV** (inset).

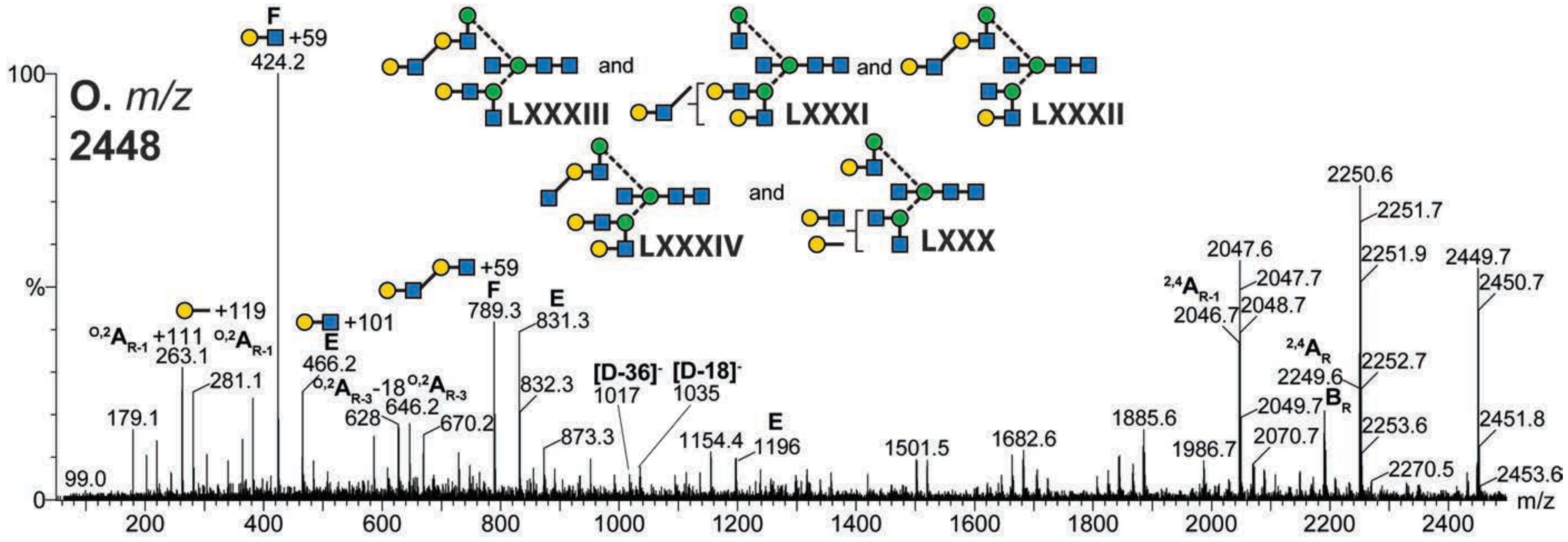

Figure S9O. Negative ion MS/MS spectrum *m/z* 2448 ($Hex_6HexNAc_7$). Mixture. $^{2,4}A_R$, B_R and $^{2,4}A_{R-1}$ ions at *m/z* 2249, 2189 and 2046 respectively (1-4-linked GlcNAc-GlcNAc core). C_1 ion at both *m/z* 179 and 220 showing hexose and HexNAc at nonreducing terminus. F ion at *m/z* 424 = Gal-GlcNAc+59. Second F ion at *m/z* 789 = 424 + 365 (Gal-GlcNAc-Gal-GlcNAc+59). No D ion but abundant [D-18]- and [D-36]- ions at *m/z* 1035 and 1017 respectively showing bisect and Glc2-GlcNAc2 in 6-antenna. *m/z* 281 = $^{O,2}A_{R-1}$ (Gal+119) and *m/z* 263 = this ion - 18. The ions at *m/z* 646 and 628 are the corresponding $^{O,2}A_{R-3}$ and $^{O,2}A_{R-3}$ - 18 ions from the N-acetyl-lactosamine extensions. *m/z* 466 = E ion (Gal-GalNAc+101) from 6- antennae, *m/z* 831 = E ion from 3-antenna = *m/z* 466 + 365 (Gal-GlcNAc). *m/z* 1196 = *m/z* 831 + 367, therefore N-acetyl-lactosamine extension also in the 3-antenna. All of the diagnostic ions had a corresponding ion 1962 mass units below. The sample contained no tetra-antennary glycans with N-acetyl-lactosamine extensions, therefore, these compounds appeared to be tri-antennary glycans with N-acetyl-lactosamine extensions on either the 3- or 6-antennae. Therefore, this spectrum is of a mixture of bisected tri-antennary compounds with N-acetyl-lactosamine extensions lacking one galactose residue from one of all antennae. Therefore, structures **LXXX, LXXXI, LXXXII, LXXXIII,** and **LXXXIV** (inset).

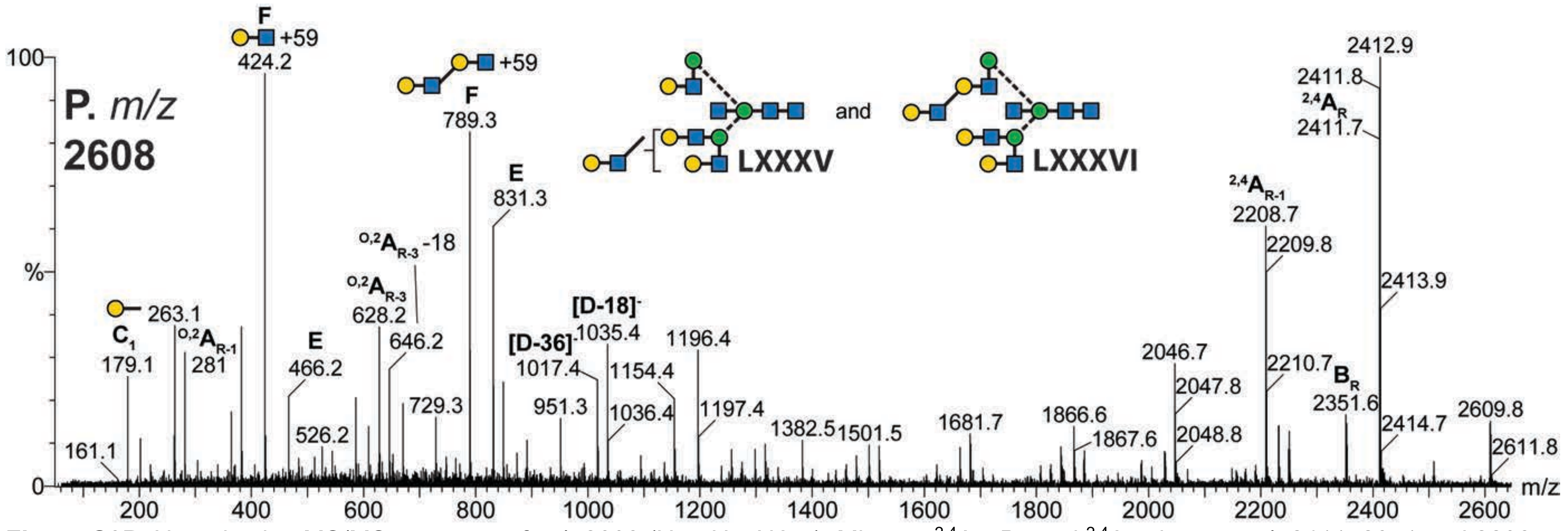

Figure S9P. Negative ion MS/MS spectrum of *m/z* 2608 ($Hex_7HexNAc_7$). Mixture. $^{2,4}A_R$, B_R and $^{2,4}A_{R-1}$ ions at *m/z* 2411, 2351 and 2208 respectively (1-4-linked GlcNAc-GlcNAc core). C_1 ion at *m/z* 179 showing hexose at non-reducing terminus (No *m/z* 220, therefore no non-reducing-terminal GlcNAc). F ion at *m/z* 424 = Gal-GlcNAc+59. Second F ion at *m/z* 789 = 424 + 365 (Gal-GlcNAc-Gal-GlcNAc+59). No D ion but abundant [D-18]- and [D-36]- ions at *m/z* 1035 and 1017 respectively showing bisect and Glc2-GlcNAc2 in 6-antenna. *m/z* 281 = $^{O,2}A_{R-1}$ (Gal+119) and *m/z* 263 = this ion - 18. The ions at *m/z* 646 and 628 are the corresponding $^{O,2}A_{R-3}$ and $^{O,2}A_{R-3}$ - 18 ions from the N-acetyl-lactosamine extensions. *m/z* 466 = E ion (Gal-GalNAc+101) from 6-antennae, *m/z* 831 = E ion from 3- antenna = *m/z* 466 + 365 (Gal-GlcNAc). *m/z* 1196 = *m/z* 831 + 367, therefore N-acetyllactosamine extension also in the 3-antenna. The sample contained no tetra-antennary glycans with N-acetyl-lactosamine extensions, therefore these compounds appeared to be bisected tri-antennary glycans with N-acetyl-lactosamine extensions on either the 3- or 6-antennae. Therefore, structures **LXXXV** and **LXXXVI** (inset).

Sialic Acid linkage characterization

Intact oligosaccharides were modified with 4-(4,6-dimethoxy-1,3,5-trazin-2-yl)-4-methyl-morpholinium chloride (DMT-MM) and analyzed by MALDI-MS to stabilize Neu5Ac and define linkages to the underlying oligosaccharide. The spectrum is shown in Fig. 8 and the glycan ions identified are listed below in Table S19. The bold ***m/z*** values represent the most abundant variants. Neu5Ac variation patterns are illustrated in Fig. 9.

Table S19. Mass spectrometry analysis of FSHα Asn[52] oligosaccharide Neu5Ac linkage

MALDI (*m/z* $[M+Na]^{+}$)		ESI (*m/z* $[M+Cl]^{-}$)												
**Found**	**Calc.**	**Found**	**Calc.**	**Mass**	**Hex**	**HexNAc**	**dHex**	**Proposed structure[a]**	**X[b]**	**No.[c]**	**Found[d]**	**Calc**	**Mass**	**Glycan**
933.3	933.3	945.3	945.3		3	2	0	**LIII**		-	-	-		
1095.4	1095.4	-	1107.3		4	2	0	**LXX**		-	-	-		
1136.4	1136.4	1148.4	1148.4		3	3	0	**LIV**						
1257.4	1257.4	?	1269.4	1234.4	5	2	0	**I**						
1282.5	1282.4	1294.4	1294.4	1259.5	3	3	1	**II**						
1298.5	1298.5	1310.4	1310.4	1275.5	4	3	0	**III**	X	 1^{3}<br>1^{6}	 **1571.6**<br>1603.6	 1571.6<br>1603.6	1275.5<br>1566.5	
1339.5	1339.5	1351.4	1351.4	1316.5	3	4	0	**IV**	X					
1444.5	1444.5	1456.5	1456.5	1421.5	4	3	1	**VI**		1^{3}<br>1^{6}	1717.7<br>1749.7	1717.6<br>1749.6		
1460.5	1460.5	1472.5	1472.5		5	3	0	**LXXII**		?<br>?	?<br>?	1733.6<br>1765.6		
1486.6	1486.5	1497.5	1497.5	1462.5	3	4	1	**VII**	X					
1501.6	1501.5	1513.5	1513.5	1478.5	4	4	0	**IX**	X	1^{3}<br>1^{6}	**1774.7**<br>1806.7	1774.6<br>1806.6		
1542.5	1542.6	1554.6	1554.5		3	5	0	**XI**						
1647.6	1647.6	1659.6	1659.6	1624.6	4	4	1	**XII**		1^{3}<br>1^{6}	1920.7<br>1952.7	1920.7<br>1952.6		
1663.6	1663.6	1675.6	1675.6	1640.6	5	4	0	**XIII**	X	1^{3}<br>1^{6}<br>$2^{3,3}$<br>$2^{3,6}$<br>$2^{6,6}$	**1936.7**<br>1968.7<br>2209.8<br>**2241.8**<br>2273.8	1936.7<br>1968.7<br>2209.8<br>2241.8<br>2273.8		
1704.6	1704.6	1716.6	1716.6	1681.6	4	5	0	**XVI**	X	1^{3}<br>1^{6}	**1977.7**<br>2009.7	1977.7<br>2009.7		
1809.6	1809.6	1821.7	1821.6	1786.7	5	4	1	**XIXa,b**	X	1^{3}<br>1^{6}<br>$2^{3,3}$<br>$2^{3,6}$<br>$2^{6,6}$	**2082.7**<br>2114.7<br>-<br>2387.8<br>?	2082.8<br>2114.8<br>2355.9<br>2387.9<br>2419.9		
1825.6	1825.6	-	1837.6		6	4	0			1^{3}	**2098.7**	2098.8		

										1^{6}	2130.8	2130.8		
1850.6	1850.7	1862.6	1862.6	1827.7	4	5	1	**XX**		1^{3}<br>1^{6}	**2123.7**<br>2155.8	2123.8<br>2155.8		
1866.7	1866.7	1878.6	1878.6	1843.7	5	5	0	**XXII**		1^{3}<br>1^{6}<br>$2^{3,3}$<br>$2^{3,6}$<br>$2^{6,6}$	**2139.8**<br>2171.8<br>2412.9<br>**2444.9**<br>2476.9	2139.8<br>2171.8<br>2412.9<br>2444.9<br>2476.9		
1907.6	1907.7	1919.7	1919.6		4	6	0			-	-	-		
2012.7	2012.7	2024.7	2024.7	1989.7	5	5	1	**XXIV**		1^{3}<br>1^{6}<br>$2^{3,3}$<br>$2^{3,6}$<br>$2^{6,6}$	2285.8<br>2317.8<br>2558.9<br>2590.9<br>?	2285.8<br>2317.8<br>2558.9<br>2590.9<br>2622.9		
2028.7	2028.7	2040.7	2040.7	2005.7	6	5	0	**XXV**	X	1^{3}<br>1^{6}<br>$2^{3,3}$<br>$2^{3,6}$<br>$2^{6,6}$<br>$3^{3,3,3}$<br>$3^{3,3,6}$<br>$3^{3,6,6}$<br>$3^{6,6,6}$	**2301.8**<br>2333.8<br>2574.9<br>**2606.9**<br>2638.9<br>2848.1<br>**2880.1**<br>2912.1<br>-	2301.8<br>2333.8<br>2574.9<br>2606.9<br>2638.9<br>2848.0<br>2880.0<br>2912.0<br>2944.0		
2069.7	2069.7	2081.7	2081.7	2151.8	5	6	0	**XXVII**	X	1^{3}<br>1^{6}<br>$2^{3,3}$<br>$2^{3,6}$<br>$2^{6,6}$	**2342.9**<br>2374.9<br>2616.0<br>**2648.0**<br>2680.0	2342.9<br>2374.9<br>2616.0<br>2648.0<br>2680.0		
2174.7	2174.8	2186.7	2186.8	2151.8	6	5	1	**XXXI**	X	1^{3}<br>1^{6}<br>$2^{3,3}$<br>$2^{3,6}$<br>$2^{6,6}$<br>$3^{3,3,3}$<br>$3^{3,3,6}$<br>$3^{3,6,6}$<br>$3^{6,6,6}$	2447.9<br>2479.9<br>2721.0<br>**2753.0**<br>2785.0<br>-<br>3026.1<br>**3058.1**<br>-	2447.9<br>2479.9<br>2721.0<br>2753.0<br>2785.0<br>2994.0<br>3026.1<br>3058.1<br>3090.1		
2215.8	2215.8	2227.7	2227.8	2192.8	5	6	1	**XXXIII**		1^{3}<br>1^{6}<br>$2^{3,3}$	2488.9<br>**2520.9**<br>2762.0	2488.9<br>2520.9<br>2762.0		

										$2^{3,6}$<br>$2^{6,6}$	2794.0<br>2827.1	2794.0<br>2827.1		
2231.8	2231.8	2244.7	2243.8	2208.8	6	6	0	**XXXV**	X	1^{3}<br>1^{6}<br>$2^{3,3}$<br>$2^{3,6}$<br>$2^{6,6}$<br>$3^{3,3,3}$<br>$3^{3,3,6}$<br>$3^{3,6,6}$<br>$3^{6,6,6}$	**2504.9**<br>2536.9<br>**2778.0**<br>2810.0<br>2842.1<br>3053.2<br>3085.2<br>3117.3<br>-	2504.9<br>2536.9<br>2778.0<br>2810.0<br>2842.1<br>3052.9<br>3084.9<br>3116.9<br>3148.9		
2377.6	2377.6	2389.8	2389.9	2354.9	6	6	1	**XXXII**	X	1^{3}<br>1^{6}<br>$2^{3,3}$<br>$2^{3,6}$<br>$2^{6,6}$<br>$3^{3,3,3}$<br>$3^{3,3,6}$<br>$3^{3,6,6}$<br>$3^{6,6,6}$	2651.0<br>**2682.9**<br>2924.1<br>**2956.1**<br>?<br>?<br>?<br>?<br>?	2651.0<br>2682.9<br>2924.1<br>2956.1<br>2988.1<br>3197.2<br>3229.2<br>3261.2<br>2393.2		
2393.8	2393.8	2405.7	2405.8	2370.9	7	6	0	**XL**	X	1^{3}<br>1^{6}<br>$2^{3,3}$<br>$2^{3,6}$<br>$2^{6,6}$	2667.0<br>2699.0<br>2940.1<br>2972.1<br>-	2667.0<br>2699.0<br>2940.1<br>2972.1<br>3004.1		
2434.8	2434.9	-	2445.9		6	7	0	**XLI**	X	1^{3}<br>1^{6}<br>$2^{3,3}$<br>$2^{3,6}$<br>$2^{6,6}$	-<br>-<br>-<br>?<br>?	2708.0<br>2740.0<br>2981.1<br>3013.1<br>3045.1		
2523.8	2523.9	-	2535.9		6	6	2			1^{3}<br>1^{6}<br>$2^{3,3}$<br>$2^{3,6}$<br>$2^{6,6}$	-<br>-<br>3086.2<br>3118.2<br>-	2813.0<br>2845.0<br>3086.1<br>3118.1<br>3150.1		
2539.8	2539.9	-	2551.9	2516.9	7	6	1	**XLIII**						
2596.8	2596.9	2608.8	2608.8	2573.9	7	7	0	**XLV**	X	1^{3}<br>1^{6}<br>$2^{3,3}$<br>$2^{3,6}$	2870.0<br>2902.0<br>?<br>?	2870.0<br>2902.0<br>3143.1<br>3175.1		

										$2^{6,6}$	?	3207.1		
2742.9	2743.0	-	2755.0	2720.0	7	7	1	**XLVI**		1^{3}<br>1^{6}	?<br>-	3016.1<br>3048.1		
2758.9	2759.0	-	2771.0		8	7	0							

[a]Roman numerals correspond to neutral glycans defined in Tables S13 or S18.
[b]X = fragmentation
[c]Number of Neu5Ac residues in the glycan. The superscript indicates the linkage, 3 = α2-3, 6 = α2-6.
[d]Bold = most abundant

Bibliography

[1] X. Jiang, H. Liu, X. Chen, P.H. Chen, D. Fischer, V. Sriraman, H.N. Yu, S. Arkinstall, and X. He, Structure of follicle-stimulating hormone in complex with the entire ectodomain of its receptor. Proc. Natl. Acad. Sci. 109 (2012) 12491-12496.

[2] X. Jiang, J.A. Dias, and X. He, Structural biology of glycoprotein hormones and their receptors: insights to signaling. Mol. Cell. Endocrinolgy 108 (2013) 7172-7176.

[3] X. Jiang, D. Fischer, X. Chen, S.D. McKenna, H. Liu, V. Sriraman, H.N. Yu, A. Goutopoulos, S. Arkinstall, and X. He, Evidence for follicle-stimulating hormone receptor as a functional trimer. J. Biol. Chem. 289 (2014) 14273-14282.

[4] B. Domon, and C.E. Costello, A systematic nomenclature for carbohydrate fragmentations in FAB-MS/MS spectra of glycoconjugates. Glycoconj. J. 5 (1988) 397-405.

[5] D.J. Harvey, Fragmentation of negative ions from carbohydrates: part 2. Fragmentation of high-mannose N-linked glycans. J. Am. Soc. Mass Spectrom. 16 (2005) 631-646.

[6] D.J. Harvey, R.L. Martin, K.A. Jackson, and C.W. Sutton, Fragmentation of N-linked glycans with a matrix-assisted laser desorption/ionization ion trap time-of-flight mass spectrometer. Rapid Commun. Mass Spectrom. 18 (2004) 2997-3007.
